# Screening for depression in children and adolescents in primary care or non-mental health settings: a systematic review update

**DOI:** 10.1186/s13643-023-02447-3

**Published:** 2024-01-31

**Authors:** Andrew Beck, Nicole Dryburgh, Alexandria Bennett, Nicole Shaver, Leila Esmaeilisaraji, Becky Skidmore, Scott Patten, Heather Bragg, Ian Colman, Gary S. Goldfield, Stuart Gordon Nicholls, Kathleen Pajer, Robert Meeder, Priya Vasa, Beverley J. Shea, Melissa Brouwers, Julian Little, David Moher

**Affiliations:** 1 Knowledge Synthesis and Application Unit, School of Epidemiology and Public Health, Faculty of Medicine, University of Ottawa, Ottawa, Ontario Canada; 2 Knowledge Synthesis Group, Clinical Epidemiology Program, The Ottawa Hospital Research Institute, Ottawa, Ontario Canada; 3 Department of Psychology, Faculty of Science, McGill University, Montreal, Canada; 4 Independent Information Specialist, Ottawa, Ontario Canada; 5 Department of Community Health Services and Department of Psychiatry, University of Calgary, Calgary, Alberta Canada; 6 Children’s Hospital of Eastern Ontario, Out-Patient Mental Health, Ottawa, Ontario Canada; 7 School of Epidemiology and Public Health, Faculty of Medicine, University of Ottawa, Ottawa, Ontario Canada; 8 Department of Pediatrics, Children’s Hospital of Eastern Ontario Research Institute, University of Ottawa, Ottawa, Ontario Canada; 9 Ottawa Hospital Research Institute, Ottawa, Ontario Canada; 10 Department of Psychiatry, uOttawa Faculty of Medicine Ottawa, Children’s Hospital of Eastern Ontario, Ottawa, Ontario Canada; 11 Department of Pediatrics, Orillia Soldiers Memorial Hospital, Orillia, Ontario Canada; 12 Department of Family and Community Medicine, St. Michael’s Hospital, University of Toronto, Toronto, Ontario Canada

**Keywords:** Depression, Screening, Systematic review, Child, Children, Adolescent, Youth

## Abstract

**Background:**

The transition from childhood to adolescence is associated with an increase in rates of some psychiatric disorders, including major depressive disorder, a debilitating mood disorder. The aim of this systematic review is to update the evidence on the benefits and harms of screening for depression in primary care and non-mental health clinic settings among children and adolescents.

**Methods:**

This review is an update of a previous systematic review, for which the last search was conducted in 2017. We searched Ovid MEDLINE® ALL, Embase Classic+Embase, PsycINFO, Cochrane Central Register of Controlled Trials, and CINAHL on November 4, 2019, and updated on February 19, 2021. If no randomized controlled trials were found, we planned to conduct an additional search for non-randomized trials with a comparator group. For non-randomized trials, we applied a non-randomized controlled trial filter and searched the same databases except for Cochrane Central Register of Controlled Trials from January 2015 to February 2021. We also conducted a targeted search of the gray literature for unpublished documents. Title and abstract, and full-text screening were completed independently by pairs of reviewers.

**Results:**

In this review update, we were unable to find any randomized controlled studies that satisfied our eligibility criteria and evaluated the potential benefits and harms of screening for depression in children and adolescents. Additionally, a search for non-randomized trials yielded no studies that met the inclusion criteria.

**Conclusions:**

The findings of this review indicate a lack of available evidence regarding the potential benefits and harms of screening for depression in children and adolescents. This absence of evidence emphasizes the necessity for well-conducted clinical trials to evaluate the effectiveness of depression screening among children and adolescents in primary care and non-mental health clinic settings.

**Systematic review registration:**

PROSPERO CRD42020150373.

**Supplementary Information:**

The online version contains supplementary material available at 10.1186/s13643-023-02447-3.

## Introduction

Major depressive disorder (MDD) is a prevalent mood disorder that can significantly impact an individual’s quality of life due to negative emotions, thoughts, and behaviors. The disorder causes impairment in social, occupational, and educational functioning and is linked to an increased risk of suicide and death [1, 2]. As individuals move from childhood to adolescence, there is a rise in the incidence of depression, which strongly tracks into adulthood making early detection paramount for timely intervention and prevention [3]. Physical, psychological, and emotional changes typical of this developmental period may increase an individual’s sensitivity and reactivity to stress exposure, which can eventually lead to depression [4, 5]. As with the adult population, diagnoses of depressive episodes (a period characterized by the symptoms of MDD) in children and adolescents are established by one of the two commonly used diagnostic classification systems for psychiatric diagnoses: the Diagnostic and Statistical Manual of Mental Disorders, Fifth Edition (DSM-5) [6], or the International Classification of Diseases, 11th Revision (ICD-11) [7]. Each diagnostic system provides a minimum number of criteria that must be met over a 2-week period for symptoms to be diagnosed as a depressive episode. In addition, the DSM-5 includes further criteria to specifically define MDD for children and youth [6]. Symptoms of irritability can be considered in place of depressed mood, and a failure to meet expected weight gain can be considered instead of weight loss (see Additional file 1).

Based on the 2014 Ontario Child Health Study, the 6-month prevalence of possible major depressive episodes (MDE) was 1.1% for children (4 to 11 years old) and 5.2% or 7.5% for adolescents (12 to 17 years old) based on parent or adolescent report, respectively [8]. In pooled estimates from the Canadian Community Health Survey, a series of cross-sectional surveys from 2000 to 2014, 5.5% of 12 to 19 year olds reported experiencing MDE-like episodes in the past year, with little change in prevalence from 2000 to 2014 [9]. Rates were higher among females than males and for those aged 15 to 19 years compared to those aged 12 to 14 years (10.1% females vs. 4.1% males and 4.1% females vs. 0.6% males, respectively) [9]. Similar findings are supported by other literature [10–12].

The burden of depression is high among children and adolescents. Persistent depressive disorders (i.e., MDE, dysthymia) are a leading cause of years lost to disability among both 10- to 14-year-old and 15- to 19-year-old age groups [13, 14]. Poor long-term social outcomes are also a consequence of depression in adolescence. Those with depression are at an increased risk of leaving secondary school early, unemployment, adolescent pregnancy, and early parenthood [15]. As well, they have a lower likelihood of entering post-secondary education [15]. Depression with onset in childhood and adolescence can continue into adulthood, posing a burden on individuals, families, and communities [15–18]. A 2018 systematic review found that adolescents who suffer from depression have around 2.5 [95% CI 1.97, 3.93] times the odds of developing depression in adulthood compared with adolescents without depression [16]. Additionally, those who suffer from depression in adolescence are at an increased risk for suicidal ideation, attempts, and completion in adulthood [19–21].

There are several risk factors associated with depression in children and adolescents. As shown above, females are at a higher risk, particularly in later adolescence, with the difference between sexes decreasing later in adulthood [12, 22]. A family history of depression and exposure to adverse events such as illness or death of a family member, or physical or sexual abuse, are also common risk factors [23, 24]. Parental behaviors associated with an increased risk include persistent negative behaviors toward the child or adolescent (e.g., neglect, criticism, punishment, and conflict), lack of autonomy given to the child or adolescent, emotional coldness, inconsistent parental discipline, and parental over-involvement [25]. Other influential factors include aspects related to the school environment such as bullying, low connectedness with peers and teachers [26, 27], poor academic achievement [28], and community environment factors such as safety, marginalized race or ethnicity and prevalence of discrimination [29]. Lifestyle factors include substance use (e.g., alcohol, cannabis, other illicit drugs), poor sleep, unhealthy diet, inactivity, excessive screen time and social media use, and weight problems [30].

The goal of a screening program for depression is to identify symptomatic disease that would not otherwise be reported (e.g., by spontaneous patient self-report, parent/caregiver report or clinical inquiry). If effective, screening for depression could lead to interventions that improve future health outcomes in those who otherwise would not have been identified [31]. However, it has been suggested that the population health effects of universal screening for depression in primary care may be low due to a failure in the health care system structures, such as adequately providing and delivering treatment [32]. Cosgrove et al. noted that without evidence on the benefits and harms of a screening program for depression, there are several components to a screening program that need to be evaluated [33]. First, unlike other disorders, depression does not have a detectable asymptomatic early stage and many patients remit after an initial episode. Screening tools, such as the Patient Health Questionnaire for Adolescents (PHQ-A), rely on identifying symptoms of depression itself and therefore can only be effective at early detection if the use of the tool prompts consideration of whether symptoms of depression are present. Second, there is currently little evidence that adding screening questionnaires to primary care reduces depressive symptoms [34, 35]. Lastly, optimal treatment for screen-detected depression is not clear [36]. Many are treated with antidepressant medications; however, the majority of antidepressant medications have not been shown to be as effective in adolescents as in adults and may be even less likely to be effective for the mild cases likely overrepresented in patients identified through screening questionnaires [37].

### Rationale

In 2005, the Canadian Task Force on Preventive Healthcare (Task Force) made a recommendation statement regarding screening for depression in children and adolescents in primary healthcare settings. However, the Task Force found insufficient evidence to recommend for or against screening [38].

Since then, three guidelines [39–41] and two systematic reviews [42, 43] have been published on this topic, but the evidence provided has been limited. These publications failed to include randomized controlled trials that separate the potential impacts of screening and treatment.

To update the Task Force guideline recommendations, a decision made by the Working Group, a recent review by Roseman and colleagues [43] was selected to use as a foundation for a systematic review update. We have made modifications to the Roseman and colleagues review to address patients at an elevated risk of depression, consider other relevant outcomes, and use an expanded search approach. This updated review will provide a current assessment of the evidence for the Task Force guideline recommendations.

### Objective

**Fig. 1:**
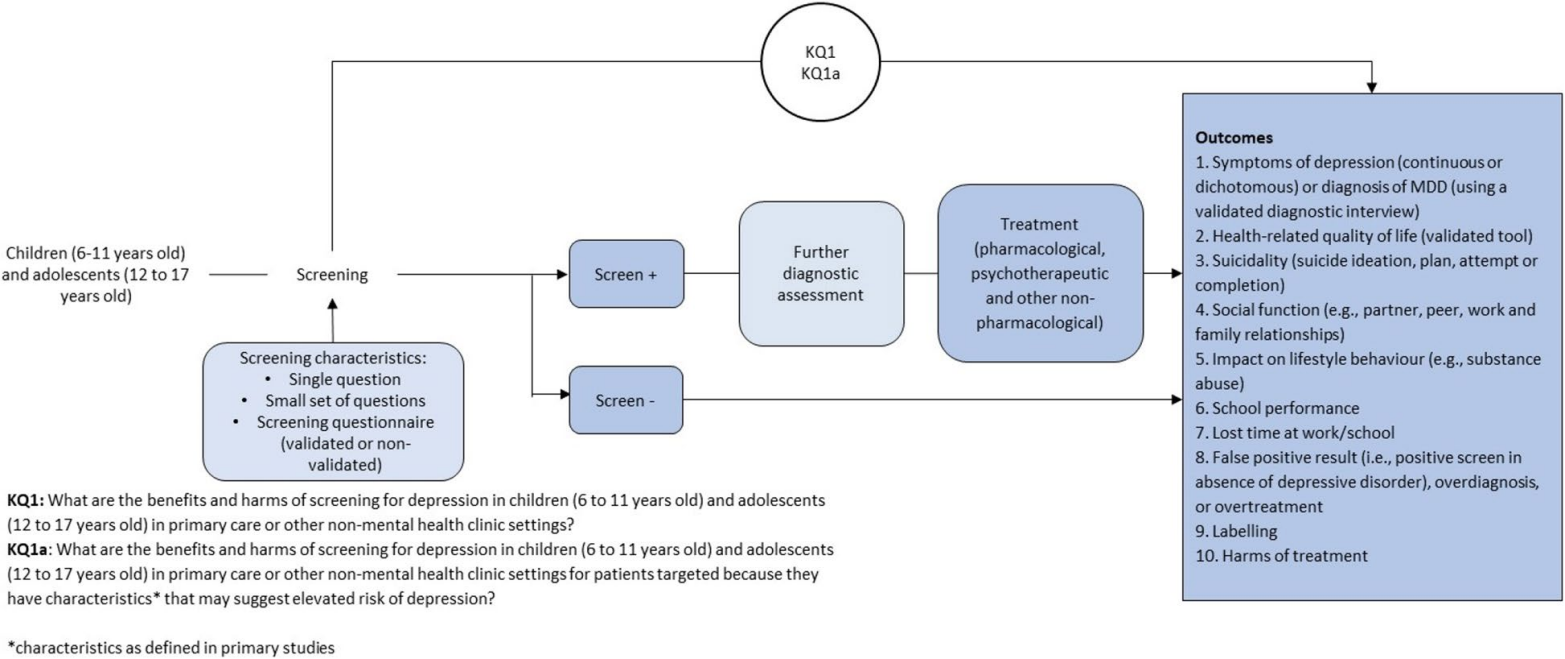
Analytic framework

The aim of this systematic review is to evaluate the benefits and potential harms of depression screening among children and adolescents in both primary care and non-mental health clinic settings. The results of this review will be used to guide the Task Force in developing their guideline recommendations. To achieve this objective, an analytic framework has been designed to address the key questions (KQ) for assessing the benefits and harms of depression screening (as shown in Fig. 1). The KQs used to guide this systematic review are outlined in Table 1.

**Table 1:** Key questions to inform an update of recommendations by the Task Force on screening for depression among children and adolescents

Key questions (KQs)	
**KQ1**	What are the benefits and harms of screening for depression in children (6 to 11 years old) and adolescents (12 to 17 years old) in primary care or other non-mental health clinic settings?
**KQ1a**	What are the benefits and harms of screening for depression in children (6 to 11 years old) and adolescents (12 to 17 years old) in primary care or other non-mental health clinic settings for patients targeted because they have characteristics that may suggest elevated risk of depression?

## Methods

### Protocol and registration

A protocol for the review was published [44] and registered with the PROSPERO database (CRD42020150373) and was made available on the Open Science Framework (https://osf.io/h5nbp/). Details on how eligibility criteria and outcomes were determined can be found in the protocol [44]. Any materials used in the review can be found on the Open Science Framework. The conduct of this review was guided by the Cochrane Handbook [45] and reported in accordance with the PRISMA 2020 guidance [46] (Additional file 2). The AMSTAR 2 tool was used for additional quality control to critically appraise systematic reviews [47].

The Depression Screening Working Group, comprised of Task Force members, collaborated with external clinical experts, the Ottawa Evidence Review and Synthesis Centre (ERSC), and the Science Team from the Global Health and Guidelines Division at the Public Health Agency of Canada to establish and finalize the key questions (KQs) and study eligibility criteria. The ERSC at the University of Ottawa Knowledge Synthesis and Application Unit conducted the review, while the Depression Screening Task Force Working Group, external clinical experts, Science Team, other Task Force members, and stakeholders were not involved in the conduct of the review. This manuscript has been approved by the Task Force and reviewed by external peer reviewers and stakeholders (see Additional file 9). There were no amendments made to the original protocol.

### Eligibility criteria

The inclusion and exclusion criteria for KQ1 and KQ1a are presented in Table 2.

**Table 2:** KQ1 and KQ1a eligibility criteria (benefits and harms of screening)

	**Inclusion**	**Exclusion**
**Population**	KQ1: Patients who are up to and including 17 years of age. KQ1a: Patients who are up to and including 17 years of age selected for screening because they have characteristics that may suggest elevated risk of depression​. For both KQs, caregivers may respond to screening questions on behalf of children. Onset of adolescence will be considered as being age 12. *Characteristics as defined in primary studies.*	>20% of the study sample are adults (18 years and older), have a recent history of depression, have a current diagnosis, or are receiving treatment for depression or other mental disorders (unless results are provided separately from the sample of interest). Members of the study sample are seeking services due to symptoms of mental disorders. Members of the study sample are receiving assessment or care in psychiatric or mental health settings. Members of the study sample are currently pregnant or have given birth in the past year.
**Intervention**	Screening tools that use a single question, a small set of questions, or a screening questionnaire (validated or non-validated) with a pre-defined cut-off score to identify patients who may have depression, but who have not previously reported their symptoms to healthcare providers or who have otherwise not been identified as possibly depressed by healthcare providers. *Patients and/or their guardians have the ability to answer screening tool questions.*	Screening tools that, in addition to screening, include depression care referral or treatment options not available to patients identified as depressed in the non-screening trial arm.
**Comparator**	No depression screening. *Patients in comparator arms may be administered depression symptom questionnaires for the purpose of baseline or outcome assessments if scores are not provided to the patients or healthcare providers prior to start of intervention.*	N/A
**Outcomes**	Critical 1. Symptoms of depression (measured continuously or dichotomously) or diagnosis of MDD (using a validated diagnostic interview) 2. Health-related quality of life (validated tool) 3. Suicidality (suicide ideation, plan, attempt, or completion) 4. Social function (e.g., partner, peer, work, and family relationships) 5. Impact on lifestyle behaviour (e.g., substance abuse) Important 1. School performance 2. Lost time at work/school 3. False-positive result (i.e., positive screen in absence of depressive disorder), overdiagnosis, or overtreatment 4. Labelling 5. Harms of treatment	N/A
**Setting**	Primary care or non-mental health clinic settings, such as medical specialist clinics, schools or recreational/community settings, and online settings (e.g., online depression screening), where screening is administered by a health practitioner.	Studies conducted in mental health, or psychiatric settings. Studies in non-mental health clinic settings where screening is administered by a non-health practitioner.
**Study design**	Randomized controlled trials (RCTs), including cluster-randomized trials.^a^ If no or only a single RCT is available, then controlled studies without random assignment.^b^	RCTs where patient eligibility is determined, and patients are enrolled after randomization. Interrupted times series, single cohort studies, case-control studies, cross-sectional studies, case series, case reports, and other publication types (editorials, commentaries, notes, letter, opinions).
**Publication language**	English or French.	Languages other than English and French.
**Dates of publication**	January 2017 to February 2021 (RCTs). January 2015 to February 2021 (non-RCTs).	

^a^Eligible RCTs needed to meet the following criteria: Wherein patient eligibility was determined and then patients were enrolled prior to randomization (i.e., to screening or to no screening); similar resources for depression management and treatment must have been available both to patients in the screening arm of the trial and to patients in the non-screening arm of the trial who were identified as depressed via other methods (e.g., unaided clinician diagnosis, patient report) [48]

^b^Eligible non-randomized controlled studies needed to meet the following criteria: Similar resources for depression management and treatment must have been available both to patients in the screening arm of the trial and to patients in the non-screening arm of the trial who were identified as depressed via other methods (e.g., unaided clinician diagnosis, patient report) [48]

#### Population

To ensure consistency with the prior review and guideline, the population of interest for both KQs included participants up to and including 17 years of age. Within this population, children were defined as those 6 to 11 years of age and adolescents as 12 to 17 years of age. The age cutoff of 17 years was chosen because the Task Force has previously addressed the adult population (18 years of age and older) in a separate review and guideline [49].

For KQ1a, we focused on participants who were selected for screening due to characteristics that may suggest an increased risk of depression, as reported in the primary studies. We excluded studies where more than 20% of the sample consisted of adults (18 years and older), individuals with a recent history of depression, current diagnosis of depression, or receiving treatment for depression or other mental disorders, unless results were reported separately from the sample of interest. Furthermore, we excluded studies involving populations seeking services due to symptoms of mental disorders, receiving assessment or care in psychiatric or mental health settings, or those who were currently pregnant or had given birth in the past year. The Task Force had previously reviewed the pregnant and postpartum population in another review and guideline [49, 50].

#### Intervention

To be considered eligible for inclusion, studies had to have used a depression screening tool that consisted of a single question, a small set of questions, or a screening questionnaire (validated or non-validated) with a pre-defined cutoff score to identify patients who may be at risk of depression. Participants or their guardians could have answered the screening tool. Moreover, to avoid the potential confounding effect of prior diagnosis or treatment, only participants who had not previously reported their symptoms to a healthcare provider or been identified as possibly depressed by healthcare providers were included.

We excluded studies that used screening tools, but also included depression care referral or treatment options that were not available to participants identified as depressed in the non-screening trial arm. This was to ensure that any observed effects of the screening tool could be attributed to the screening process itself rather than the availability of additional care or resources.

#### Comparator

We included studies where the comparator group did not undergo depression screening. However, in cases where depression symptom questionnaires were administered to participants in the comparator group for the purpose of baseline or outcome assessments, these were included if scores were not provided to the patients or healthcare providers prior to start of intervention.

#### Outcomes

To determine the importance of outcomes for decision-making, the Working Group members reviewed and rated them based on consensus, with input from external clinical experts. The outcomes were assessed using the GRADE methodology, which classified them as critical (rated 7 to 9 out of 9), important (rated 4 to 6 out of 9), or of limited importance (rated 1 to 3 out of 9) for making guideline recommendations [51]. For the systematic review, only critical and important outcomes were included.

The outcomes were also subject to review by stakeholders and patient representatives as part of the Task Force’s patient engagement activities, which were facilitated by the Knowledge Translation Program at St. Michael’s Hospital in Toronto, Ontario [52]. This ensured that patient perspectives were considered when assessing the importance of the outcomes.

The Working Group rated several outcomes as critical, including symptoms of depression or diagnosis of MDD, health-related quality of life, suicidality (including suicide ideation, plan, attempt, or completion), social functioning (e.g., partner, peer, work, and family relationships), and impact on lifestyle behaviour (e.g., substance abuse). These outcomes were considered essential for making clinical decisions.

Additionally, several outcomes were rated as important, including school performance, lost time at work/school, false-positive results (i.e., positive screen in absence of depressive disorder), overdiagnosis, overtreatment, labeling, and harms of treatment. While not considered critical, these outcomes were still deemed significant for making informed clinical decisions.

#### Study design

We prioritized the inclusion of randomized controlled trials (RCTs), including cluster RCTs. Non-randomized studies were considered if no RCTs were available or only one RCT was found and did not provide sufficient evidence to inform a recommendation.

To ensure that studies met the criteria for inclusion as a depression screening study, we applied the following criteria [48, 53]: First, the patient population must have been clearly defined and randomized before administering the screening tool (applied only to RCTs). Second, patients diagnosed with depression or those who were already receiving treatment for depression were excluded, as the purpose of screening is to identify undetected cases. Third, similar resources for depression management and treatment must have been available to patients in the screening arm of the trial and to patients in the non-screening arm of the trial who were identified as depressed via other methods (e.g., unaided clinician diagnosis, patient report).

#### Publication language and date

We included articles written in English or French. Articles published in languages other than English or French were excluded studies from our review. For RCTs, we included articles published from 2017 onwards. This was in line with the last search date used in the Roseman et al. review [43]. For non-randomized studies, we included studies from 2015 onwards. This was because the 2016 USPSTF review on depression screening in children and adolescents did not identify any non-randomized studies assessing the effects of screening, and their last search date was February 2015 [42]. By including non-randomized studies published from 2015 onwards, we aimed to identify any additional evidence that had been published since the USPSTF review.

### Information sources and search strategy

The search strategies were developed by an experienced medical information specialist in consultation with the ERSC. The MEDLINE strategy was peer-reviewed by another senior information specialist prior to execution using the PRESS Checklist (Additional file 3) [54].

For the RCT search, we searched Ovid MEDLINE® ALL, Embase Classic+Embase, APA PsycINFO, and EBM Reviews - Cochrane Central Register of Controlled Trials on the Ovid platform (where we used the multifile option and the internal deduplication tool). We also searched CINAHL on Ebsco. All searches were performed on 4 November 2019 and updated on 19 February 2021. Strategies utilized a combination of controlled vocabulary (e.g., “Depressive Disorder”, “Mass Screening”, “Adolescent”) and keywords (e.g., “depression”, “screening”, “child”). We used an amended version of the 2008 Cochrane Highly Sensitive Search Strategy, sensitivity- and precision-maximizing version, to identify RCTs. Vocabulary and syntax were adjusted across databases. All searches were limited to the update period 2017 to the present. When possible, animal-only and opinion pieces were removed from the results. Specific details regarding the strategies appear in Additional file 4.

For the non-randomized study search, we searched the same databases except for Cochrane Central Register of Controlled Trials. We applied a non-randomized controlled trial filter, limited the update period from 2015 to the present, and when possible, removed animal-only and opinion pieces where possible. All searches were performed on 27 September 2020 and updated on 14 February 2021. The 2021 records were updated on February 22. Specific details regarding the strategies appear in Additional file 4.

We searched gray literature sources for unpublished documents using the Canadian Agency for Drugs and Technologies in Health (CADTH) Gray Matters checklist [55]. In addition, we also searched websites of relevant organizations as suggested by the Task Force and clinical experts. The list of websites searched is available in Additional file 5. The literature search was supplemented by reviewing references of relevant secondary evidence reports that were retrieved (e.g., evidence-based clinical practice guidelines, systematic reviews, and meta-analyses). To be considered a systematic review the following criteria was required [56]: (1) At least one database was searched, (2) authors reported selection criteria, (3) risk of bias (or intended analogous) of included studies was reported, and (4) authors reported a list and synthesis of included studies. Further, working group members and clinical experts were contacted for potentially missing studies.

### Study selection

The citations retrieved from the searches were uploaded into an online systematic review management software package, DistillerSR© [57], and duplicates from across the databases were removed. Pilot tests of the screening forms were completed by two reviewers prior to title and abstract screening (random sample of 50 citations) and full-text article review (random sample of 25 articles). Any conflicts among the reviewers were resolved through discussion before starting each screening level.

The title and abstract screening were performed independently by two reviewers using the liberal accelerated method [58]. One reviewer screened citations and the second reviewer verified the first reviewer’s excluded citations. Citations were screened in random order and completed concurrently to reduce the likelihood that a reviewer would know a citation had already been considered by another reviewer. Discrepancies among reviewers were not discussed at this stage and citations with conflicting answers advanced to the full-text article review.

Full-text article review involved the same two reviewers who independently and in duplicate reviewed the full-text articles of potentially relevant studies. Conflicts were resolved by consensus or by consulting with a senior team member. The list of excluded studies and reasons for exclusion was documented and is available in Additional files 6 and 7.

### Data extraction, risk of bias assessments, synthesis, and certainty of the evidence

As described in our study protocol [44], we planned to extract data from the included studies, perform risk of bias assessments, conduct analyses, and assess the certainty of the evidence if any studies met our inclusion criteria. However, despite our comprehensive search strategy, we were unable to identify any eligible studies on depression screening in children and adolescents. As a result, we were unable to perform these stages of the review.

## Results

**Fig. 2:**
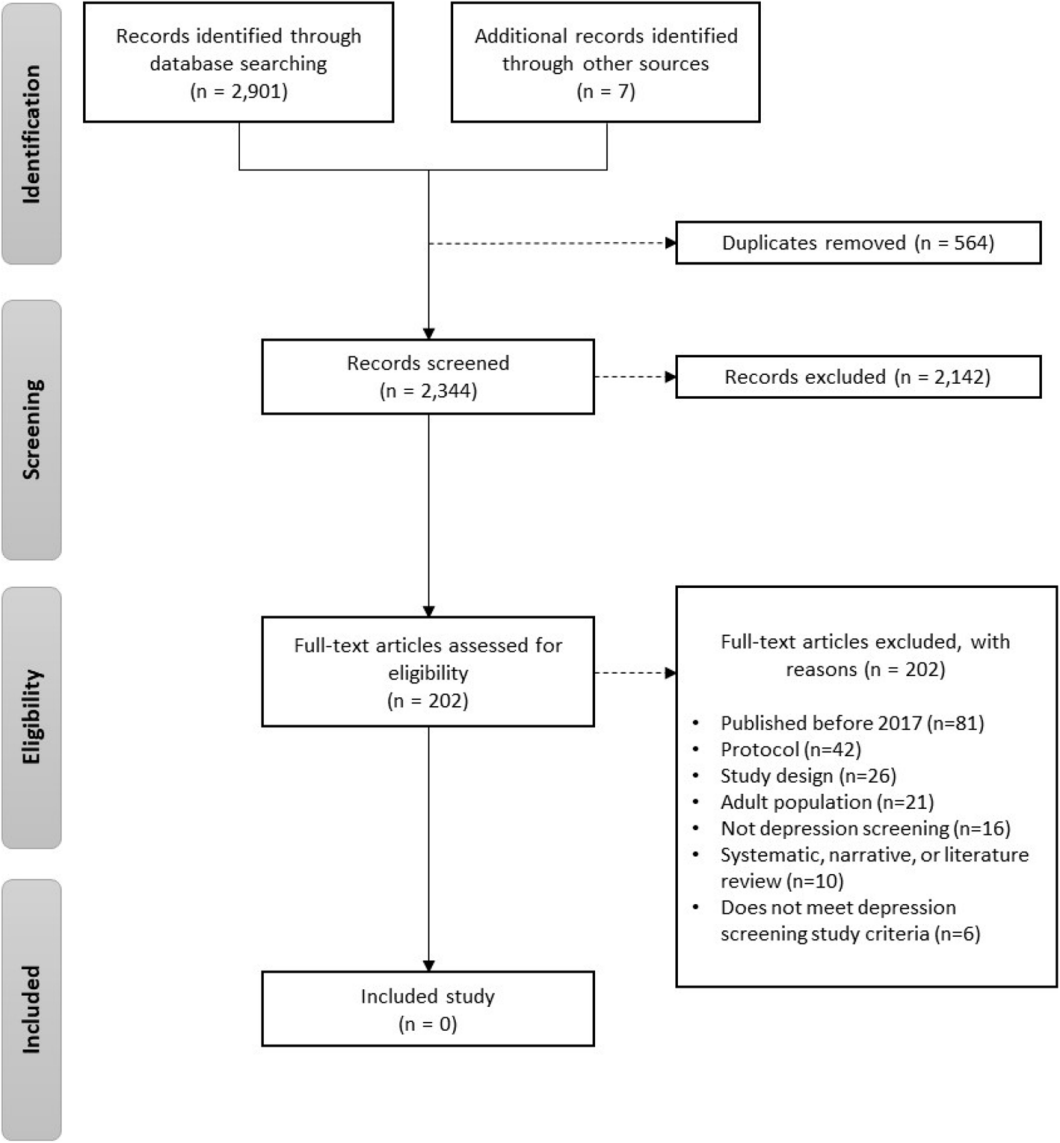
PRISMA flow diagram (RCT)

Our initial search strategies for RCTs resulted in 2901 citations with an additional seven records found from gray literature searching. After de-duplication, 2344 titles and abstracts were screened, and 2142 citations were excluded for not meeting the eligibility criteria. A total of 202 full-text articles were retrieved for full-text review. All of them were excluded. No RCTs of depression screening met the inclusion criteria, and therefore, no articles were included in this review. We did identify four ongoing studies (Additional file 8). The study selection flow chart is shown in Fig. 2.

**Fig. 3:**
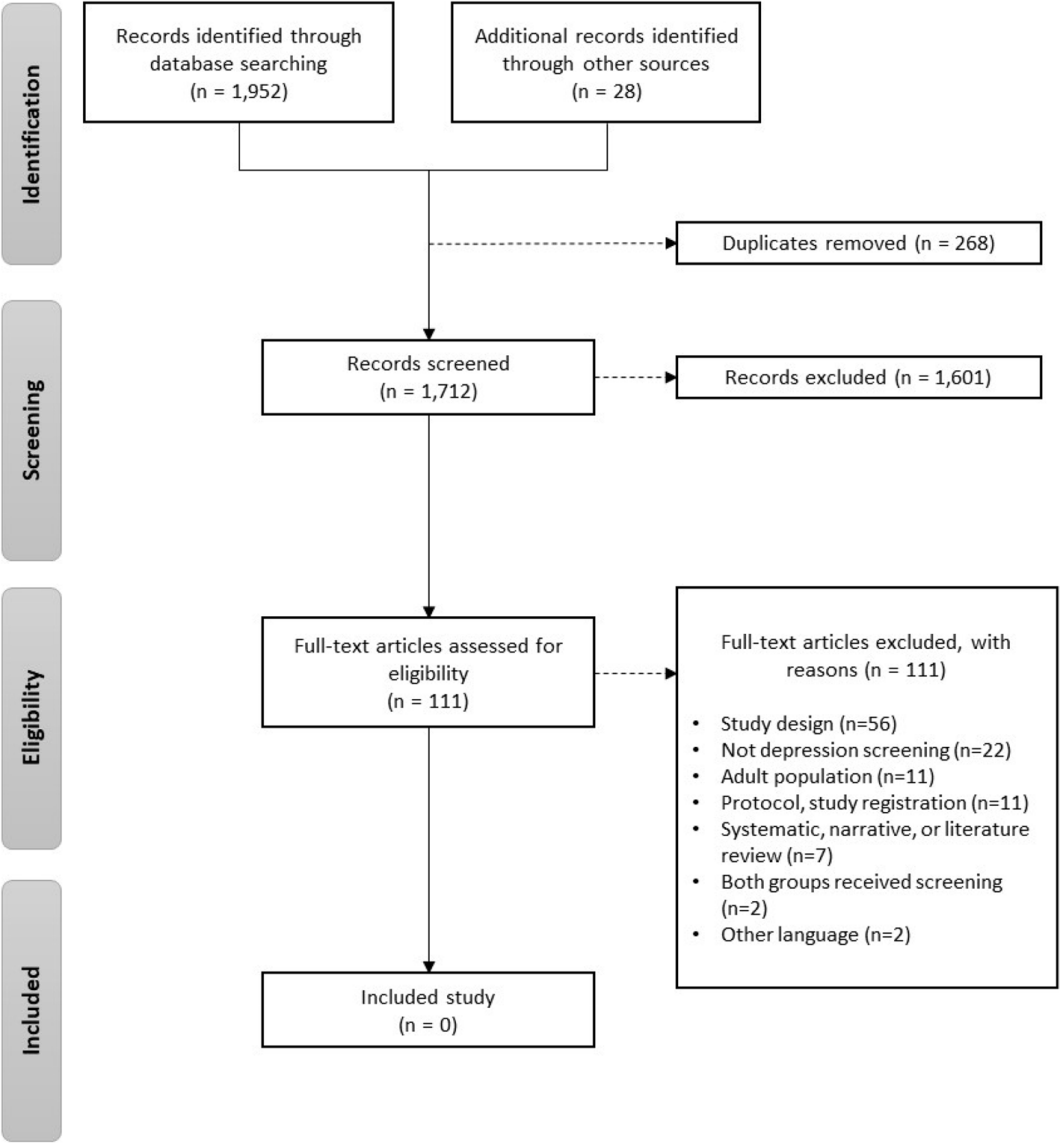
PRISMA flow diagram (non-randomized controlled studies)

Since we found no RCTs, we conducted additional planned searches for non-randomized studies with a control group. Our searches retrieved 1952 citations with an additional 28 records found from gray literature searching. After de-duplication, 1712 titles and abstracts were screened, and 1601 citations were excluded. A total of 111 full-text articles were retrieved for full-text review, and none of them met the inclusion criteria. The study selection flow chart is shown in Fig. 3.

This empty review did not identify any studies that met inclusion criteria, and some studies partially met the criteria but were ultimately excluded (see Table 3). Eight studies satisfied some elements of the inclusion criteria but were ultimately excluded (Table 3). Among RCTs, six were excluded at the full-text screening stage because they did not meet the criterion of a depression screening study [59–64]. Three of these trials provided limited information on the study population characteristics, making it unclear whether participants met the population exclusion criteria (i.e., recent history of depression, current diagnosis, or receiving treatment for depression or other mental disorders) [59, 60, 63]. Two RCTs were excluded because both groups received depression screening or assessment [62, 63], while two other RCTs were excluded because they included individuals who already had symptoms or a diagnosis of depression [61, 64]. Among non-randomized studies, two were excluded because they did not meet the criteria of a depression screening study due to both the intervention and control groups receiving depression screening [65, 66]. Despite these exclusions, the reviews emphasize the need for well-designed studies that can provide evidence of the benefits and harms of depression screening in children and adolescents.

**Table 3:** Study characteristics of excluded studies that satisfied part of the inclusion criteria

**Author year, country**	**Study details**	**Population characteristics**	**Intervention and comparator**	**Outcomes**	**Reason for exclusion**
**Randomized trials**					
Guo 2017 USA [59]	Cluster RCT to examine the effects of universal depression screening	Elementary school setting involving seventh and eighth grade Asian American and Latino students	**Intervention:** Universal depression screening using the Patient Health Questionnaire for Adolescents (PHQ-A) **Control:** No mental health screening	Referral, acceptance, and receipt of care	No primary outcomes related to mental health reported. The study evaluated school-based mental health service referrals, caregiver consent for services, and treatment initiation.
Mahoney 2017 USA [64]	Multisite RCT to understand the internal barriers of implementing a targeted preventive intervention, CATCH-IT	Adolescents between the ages of 13 and 18	**Intervention:** CATCH-IT, a 14-module Internet-based depression prevention intervention that involves mental health screening and depression prevention treatment. **Control:** General health education	Internal barriers to successful implementation using REACH framework (proportion of target audience exposed to intervention)	Focused on barriers to implementing a preventive intervention and included those with either a past major depressive disorder diagnosis or a CES-D score of 8 to 17.
Mirzaie 2019 Afghanistan [63]	Sought to validate the Maria Kovacs Children’s Depression Inventory to assess depression.	High school students in Afghanistan (grades 7 to 9)	**Intervention:** Maria Kovacs children’s depression inventory **Control:** Beck’s depression Inventory	Validity, reliability, sensitivity, specificity, and positive and negative predictive values	Both groups were offered screening tools and limited information was provided on the included students.
Rinke 2019 USA [60]	Stepped-wedge cluster RCT of quality improvement collaborative (QIC)	Pediatric primary care clinics including health care providers trained in quality improvement and diagnoses of adolescent depression	**Intervention:** Quality improvement collaborative **Control:** No attempt for quality improvement	Frequency of recognition and diagnosis of adolescent depression	Information on participant characteristics limited, therefore, unclear whether study included participants with characteristics that were part of our exclusion criteria. The study did not report a pre-defined cut-off score to identify patients who may have depression.
Sterling 2018 USA [61]	Pragmatic cluster randomized implementation and effectiveness trial on delivering Screening and Brief Intervention and Referral to Treatment (SBIRT)	Adolescents (age 12 to 18) who screened positive in a general pediatric primary care clinic	**1**^**st**^** arm:** Pediatrician-only, in which pediatricians were trained to delivery SBIRT **2**^**nd**^** arm:** embedded behavioural clinician (BC), in which pediatricians refer eligible adolescents to a BC who administered SBIRT **Control:** Usual care	Substance use and depression measures	Focused on identifying and delivering early intervention and treatment services to individuals at risk of developing substance use disorders and those who have already developed these disorders. Included adolescents who endorsed substance use or depression symptoms or were eligible for further assessments.
Thabrew 2019 New Zealand [62]	RCT to compare the performance and acceptability of YouthCHAT screening and HEEADSSS assessment	13-year-old high school students attending a nurse-led clinic	**Intervention:** YouthCHAT, a depression screening tool based on the PHQ-A **Control:** HEEADSSS, assessment, a psychosocial interview-based assessment to identify mental health and substance use problem.	Completion times, detection rates, and acceptability	Both groups received depression screening or assessment. There was no control group who received no depression screening.
**Non-randomized trials**					
Carrozzino 2016 Italy [65]	Clinimetric validation analysis of the Kellner Symptom Questionnaire and the Screen for Children Anxiety Related Emotional Disorders (SCARED) scales for depression and anxiety screening in adolescents	Adolescents with epilepsy, using participants without epilepsy as the control group.	**Group:** Patients with epilepsy **Control:** Patients without history of epilepsy disorder or any diagnosis of neurological disease or chronic medical illness	Validity, reliability, and frequency of recognition and diagnosis of adolescent depression	Validation study. There was no control group who received no depression screening. Did not exclude those who had a history of depression or who were already under treatment.
Harder 2019 USA [66]	Quality improvement study to evaluate the impact of a quality improvement learning collaborative on adolescent depression screening.	Medical files from seventeen pediatric serving (pediatric and family medicine) practices.	**Group:** Practices voluntarily participated in quality improvement initiative **Control:** Practices did not participate in the quality improvement initiative	Frequency of screening and documenting of initial plans of care	Not a depression screening study. No control group who did not receive depression screening. No information was reported on the adolescent population and limited information on the control group.

## Discussion

We did not find any RCTs examining the benefits and harms of screening for depression in children and adolescents since no new evidence has been published since Roseman and colleagues’ 2017 systematic review [43]. We expanded our search to include non-randomized studies and studies examining patients with an elevated risk of depression, as well as studies assessing other outcomes relevant to decision-making to update the Task Force guideline recommendations, a decision made by the Working Group. However, this resulted in no additional studies being included, ultimately leading to an empty review. These findings emphasize the urgent need for high-quality clinical trials that can provide direct evidence on the benefits and harms of depression screening among children and adolescents.

Some existing guidelines and systematic reviews have focused on the accuracy of depression screening tools, but it is important to note that accuracy alone does not necessarily justify the use of such tools. While screening tools can effectively detect depression, this indirect evidence should not be used to suggest that they are automatically beneficial or necessary in all contexts. It is important to consider the potential benefits and harms of screening in the specific population and setting in question and to carefully evaluate the implications of implementing a screening program. Unfortunately, we did not find any studies, whether RCTs or non-randomized studies, that provide evidence of the effectiveness depression screening in reducing the severity of depression symptoms or the frequency of episodes among children and adolescents. Moreover, we did not find any evidence suggesting that depression screening improves the quality of life; reduces risk of suicide ideation, attempts, or completions; enhances social functioning; or affects risk behaviors among children and adolescents.

During the full-text screening stage, a total of 202 studies were identified for the search for RCTs and 111 for the search for non-randomized studies with a control group. Unfortunately, none of these studies met all the inclusion criteria (Figs. 2 and 3). We provide a summary of the common reasons for exclusion to inform future research on depression screening in children and adolescents. Some studies were excluded because the entire sample received depression screening, leaving no control group for comparison [62–66]. For instance, Thabrew et al. [58] conducted an RCT comparing the YouthCHAT electronic screener to the HEEADSSS in-person screener but did not include a control group that received no depression screening. Other studies did not exclude those with depression [61] or provided limited information on their participant characteristics [59, 60]. As the purpose of screening is to identify cases that were previously undetected [67], the evidence for screening should be based on studies with patients who are not already diagnosed or under care for depression. Furthermore, several studies did not differentiate the effects of screening from those of a treatment intervention 59, 60]. It has been suggested that screening for depression may not have an impact on treatment, as the uptake of patients receiving treatment is not happening at a high rate [32, 59]. One paper, by Guo et al., found that while screening was associated with referrals, there was no difference in treatment initiation between the screening and unscreened groups, suggesting that screening leads to over referral [59]. More well-designed trials are needed to isolate the effects of screening compared to no screening, to better inform primary care recommendations and improve mental health outcomes.

Both the USPSTF and the Guidelines for Adolescent Depression in Primary Care (GLAD-PC) guidelines recommend depression screening for adolescents, despite the lack of direct evidence for harms and benefits of screening at this age. The USPSTF’s most recent guideline published in 2016 recommended routine screening for major depressive disorder in adolescents ages 12 to 18 and not children ages 11 or younger [39]. Similarly, the GLAD-PC guidelines published in 2018 gave a “very strong” recommendation that adolescent patients, 12 years and older, be screened yearly for depression in primary care [40]. However, both guidelines rely on indirect evidence to support their recommendations, such as studies on the psychometric properties of screening tools, including their sensitivity and specificity for identifying individuals with depression, as well as the feasibility, effectiveness, and harms of receiving treatment as opposed to screening [40, 42]. A lack of direct evidence for these outcomes in children was cited by the USPSTF for their decision not to recommend screening in youth younger than 12 years [49]. In their recommendation to screen for depression for adolescents, GLAD-PC argued that the lack of trials on benefits or harms of screening for adolescents was “less relevant” given evidence for the validity of screening tools and feasibility and efficacy of implementing treatment [40]. The lack of RCTs on depression screening has raised concerns about the reliance on indirect evidence to inform guidelines [33]. The USPSTF have updated their 2016 guideline on screening for depression [68], but their updated systematic review, completed in July 2021 with a search update on December 2021, found no RCTs that provided direct evidence for the benefits or harms of depression screening for children or adolescents [69]. The current findings suggest that there remains a lack of evidence to support recommendations to screen for depression in children and adolescents in primary care settings.

In contrast to these guidelines, other organizations like the National Institute for Clinical Excellence (NICE) in the UK do not recommend routine depression screening for youth. Instead, they recommend watchful waiting for those who begin to report symptoms of depression and training for professionals around identifying and evaluating risk factors [41, 70]. Similarly, the UK National Screening Committee does not recommend screening for depression in adults and has no recommendation for screening in children and adolescents [71]. 

### Limitations of the current review

The reviews conducted followed a rigorous protocol, including peer review evaluation of the search strategies, gray literature searching, and an update of a previously published systematic review to avoid research waste and reduce duplication of effort. Although there is a small potential for missing relevant studies published in languages other than English and French, we believe that our inclusion criteria would not have included the two potentially relevant publications in other languages. One study focused on psychopathology screening in adult medical school students [72], while the other was an overview of routine screening and prevention programs for 6- to 18-year-old youth for supporting Austrian recommendations [73]. Therefore, we believe that our reviews have adequately captured relevant evidence on depression screening in children and adolescents or the lack of it.

### Implications for research

More research is needed to determine the effectiveness of depression screening, particularly in primary care settings, and the outcomes of screening should be examined using high-quality study designs [33]. An ideal study to evaluate the benefits and harms of depression screening in children and adolescents would enroll a population of individuals up to and including 17 years of age who are seeking care in primary care or non-mental health clinic settings. Participants should be randomly assigned to one of two groups: an experimental group that undergoes depression screening by a healthcare practitioner or a control group that receives no screening for depression. The screening tool should have a pre-defined cutoff score and be validated for the specific age group. Relevant extensions of the Consolidated Standards of Reporting Trials (CONSORT) statement [74] should be utilized when reporting randomized trials. Conducting well-designed RCTs such as these can provide direct evidence for the benefits or harms of screening for depression in primary care settings.

When designing future trials to evaluate depression screening in children and adolescents, it is important to assess critical outcomes such as symptoms of depression or a diagnosis of MDD before and after screening (evaluated using a validated diagnostic interview such as the KSADS), health-related quality of life, suicidality (including ideation, plan, attempt, or completion), social functioning (e.g., relationship quality with a romantic partner, peers, family, and work), and lifestyle behavior (such as substance use). Additionally, other important outcomes to consider include false-positive results, overdiagnosis, overtreatment, harms of treatment or labeling, school performance, and lost time at work or school. To ensure that future trials evaluate and report outcomes relevant to depression screening in youth, trials may benefit from developing a core set of outcomes aligned with those promoted by the Core Outcomes Measures in Effectiveness Trials (COMET) initiative. By doing so, we can improve our understanding of the benefits or harms of depression screening in children and adolescents and ultimately improve their mental health outcomes.

Researchers have started investigating the perspectives of young people on depression screening. Thabrew et al. [62] evaluated the acceptability of electronic and face-to-face screening instruments among adolescents and found that some participants were hesitant about screening in both conditions, and not all reported feeling safe answering the questions. Another pre-registered systematic review aims to identify barriers and facilitators to adolescent depression screening in primary care settings [75]. This review once completed, could provide valuable insights into the perspectives of young people on depression screening, which may have implications for its potential benefits or harms. As our understanding of the impact of depression screening on young people is limited, studies on this topic can help inform and contextualize research on screening outcomes.

## Conclusion

Our systematic review of the literature did not yield any evidence on the benefits or harms of screening for depression in children and adolescents. As a result, we are unable to draw conclusions about whether screening has an effect on the outcomes of interest. Our findings underscore the need for further research in this area to inform clinical practice and policy decisions. The uncertainty surrounding the benefits or harms of depression screening in children and adolescents in primary care or non-mental health clinic settings highlights the importance of carefully considering the potential risks and benefits of any proposed screening programs. It is crucially important that future studies are well-conducted and adequately reported, including randomized controlled trials that evaluate screening versus no screening in this population. Given the significant public health burden of depression in children and adolescents, it is essential that we continue to investigate effective ways to identify and manage this condition.

### Supplementary Information

**Additional file 1.** DSM-5 and ICD-10 definition of major depressive episode.

** Additional file 2.** PRISMA 2020 checklist.

** Additional file 3.** Completed PRESS form.

** Additional file 4.** Database search strategies.

** Additional file 5.** List of websites searched (grey literature search).

** Additional file 6.** List of excluded studies with reasons (randomized controlled trials). 

** Additional file 7.** List of excluded studies with reasons (non-randomized controlled studies).

** Additional file 8.** List of potentially relevant ongoing studies.

** Additional file 9.** Stakeholder feedback.

## Data Availability

Not applicable.

## Abbreviations

CI: Confidence interval
DSM: Diagnostic and Statistical Manual of Mental Disorders
ERSC: Evidence Review and Synthesis Centre
GLAD-PC: Guidelines for Adolescent Depression in Primary Care
GRADE: Grading of Recommendations Assessment, Development and Evaluation
ICD: International Classification of Diseases
KQ: Key question
MDD: Major depressive disorder
MDE: Major depressive episodes
USPSTF: United States Preventive Services Task Force
