# Supplementary material for: Screening for depression in children and adolescents in primary care or non-mental health settings: a systematic review update
Source: Syst Rev. 2024 Jan 31;13:48. doi: 10.1186/s13643-023-02447-3 (PMC10829174; doi:10.1186/s13643-023-02447-3)
Supplement: Supplementary file 3 — Additional file 3. Completed PRESS form. [file 13643_2023_2447_MOESM3_ESM.docx]

## Additional file 3: Completed PRESS form

*PRESS Guideline* 2015— Search Submission & Peer Review Assessment

Reference: McGowan J, Sampson M, Salzwedel DM, Cogo E, Foerster V, Lefebvre C. PRESS Peer Review of Electronic Search Strategies: 2015 guideline statement. *J Clin Epidemiol* 2016;75:40-6. Available: http://www.jclinepi.com/article/S0895-4356(16)00058-5/pdf.

**Search submission: This section to be filled in by the searcher**

Searcher: Becky Skidmore Email: bskidmore@rogers.com

Date submitted: 2019 Sep 8 Date requested by: 2019 Sep 12

| 1. **Systematic Review Title** |  |
| --- | --- |

Screening for depression in children and adolescents

| 1. **This search strategy is …** |
| --- |

| X | My PRIMARY (core) database strategy — First time submitting a strategy for search question and database |
| --- | --- |
|  | My PRIMARY (core) strategy — Follow-up review NOT the first time submitting a strategy for search question and database. If this is a response to peer review, itemize the changes made to the review suggestions |
|  | SECONDARY search strategy— First time submitting a strategy for search question and database |
|  | SECONDARY search strategy — NOT the first time submitting a strategy for search question and database. If  this is a response to peer review, itemize the changes made to the review suggestions |

| 1. **Database** (e.g., MEDLINE, CINAHL) *[mandatory]* |
| --- |

MEDLINE

| 1. **Interface** (e.g., Ovid, EbscoHost…) *[mandatory]* |
| --- |

Ovid

| 1. **Research Question** (Describe the purpose of the search)  *[mandatory]* |
| --- |

**KQ1**: What are the benefits and harms of screening for depression in children (6 to 11 years old) and adolescents (12 to 17 years old) in primary care or other non-mental health clinic settings?

**KQ1a**: What are the benefits and harms of screening for depression in children (6 to 11 years old) and adolescents (12 to 17 years old) in primary care or other non-mental health clinic settings for patients targeted because they have characteristics that may suggest elevated risk of depression?

| 1. **PICO Format** Outline the PICOs for your question — i.e., Patient, Intervention, Comparison, Outcome, and Study Design — as applicable |
| --- |

| **P** | Children (6 to 11 years old) and adolescents (12 to 17 years old)  KQ1: Patients who are up to and including 17 years of age.  KQ1a: Patients who are up to and including 17 years of age selected for screening because they have characteristics* that may suggest elevated risk of depression​.  For both KQs, caregivers may respond to screening questions on behalf of children. Onset of adolescence will be considered at age 12.  Setting: Primary care or non-mental health clinic settings such as medical specialist clinics, schools or recreational/community settings, and online settings (e.g., online depression screening) |
| --- | --- |
| **I / Exposure** | Screening tools that use a single question, a small set of questions, or a screening questionnaire (validated or non-validated) with a pre-defined cut-off score to identify patients who may have depression, but who have not reported their symptoms to healthcare providers or who have otherwise not been identified as possibly depressed by healthcare providers. |
| **C** | No depression screening. |
| **O** | Critical:  Symptoms of depression (continuous or dichotomous) or diagnosis of MDD (using a validated diagnostic interview)  Health-related quality of life (validated tools)  Suicidality (suicide ideation, plan, attempt or completion)  Social function (e.g., partner, peer, work and family relationships)  Impact on lifestyle behaviour (e.g., substance abuse)  Important:  School performance  Lost time at work/school  False-positive result (i.e., positive screen in absence of depressive disorder), overdiagnosis, or overtreatment  Labelling  Harms of treatment |
| **S** | Randomized controlled trials (RCTs), including cluster-randomized trials. |

| 1. **Inclusion Criteria** (List criteria such as age groups, study designs, etc., to be included) *[optional]*   **This search strategy is …** |
| --- |

January 2017 to present

| 1. **Exclusion Criteria** (List criteria such as study designs, date limits, etc., to be excluded) **[optional]** |
| --- |

Animal-only and opinion pieces

| 1. **Was a search filter applied?** Yes |
| --- |

**If YES, which one(s) (e.g., Cochrane RCT filter, PubMed Clinical Queries filter)? Provide the source if this is a published filter.** *[mandatory if YES to previous question* — *textbox]*

Amended 2008 Cochrane HSSS, sensitivity- and precision-maximizing version

| 1. **Notes or comments you feel would be useful for the peer reviewer**  *[optional]* |
| --- |

Original strategies included instruments/scales – these have since been removed:

“…have been asked to run the search without the screening tool names in it. One of the members is an expert in this area and is pretty adamant that it introduces inefficiencies and that we would not be criticized for not including them.”

| 1. **Please copy and paste your search strategy here, exactly as run, including the number of hits per line. [mandatory]** |
| --- |

Database: Ovid MEDLINE(R) ALL <1946 to September 06, 2019>

Search Strategy:

--------------------------------------------------------------------------------

1 exp Depressive Disorder/ (105006)

2 Depression/ (111367)

3 depress*.tw,kf. (439182)

4 dysthym*.tw,kf. (3109)

5 blues.tw,kf. (1723)

6 melanchol*.tw,kf. (2973)

7 MDD.tw,kf. (12009)

8 or/1-7 [GENERAL DEPRESSION] (481808)

9 Mass Screening/ (98864)

10 (screen* or detect*).tw,kf. (2759757)

11 (identif* or recogni*).ti. (361541)

12 ((early or earlier or earliest) adj5 (identif* or recogni*)).tw,kf. (64785)

13 (case finding? or casefinding?).tw,kf. (5060)

14 or/9-13 [GENERAL SCREENING] (3104769)

15 8 and 14 (50093)

16 Adolescent/ (1954186)

17 Child/ (1631763)

18 Minors/ (2516)

19 (boy or boys or girl or girls or child* or school-age* or adolescen* or teen or teens or teenage* or youth or youths or highschool* or high-school* or juvenil* or pubescen* or under?age*).tw,kf. (1726182)

20 p?ediatric*.tw,kf. (340506)

21 or/16-20 [CHILD/ADOLESCENT POPULATION] (3443588)

22 15 and 21 [SCREENING - DEPRESSION - CHILD/ADOLESCENTS] (11505)

23 exp Animals/ not Humans/ (4615945)

24 22 not 23 [ANIMAL-ONLY REMOVED] (11382)

25 (comment or editorial or news or newspaper article).pt. (1346037)

26 (letter not (randomized controlled trial and letter)).pt. (1036273)

27 24 not (25 or 26) [OPINION PIECES REMOVED] (11314)

28 (controlled clinical trial or randomized controlled trial or pragmatic clinical trial or equivalence trial).pt. (577847)

29 clinical trials as topic/ (188234)

30 exp Randomized Controlled Trials as Topic/ (128933)

31 (randomi#ation? or randomi#ed or randomly or RCT or placebo*).tw,kf. (930310)

32 ((singl* or doubl* or trebl* or tripl*) adj (mask* or blind* or dumm*)).tw,kf. (166257)

33 trial.ti. (204315)

34 or/28-33 (1345454)

35 27 and 34 [RCTs] (1277)

36 (2017* or 2018* or 2019*).dt. (3373127)

37 35 and 36 [UPDATE PERIOD] (230)

***************************

**Peer review assessment: this section to be filled in by the reviewer**

|  | Reviewer: Kaitryn Campbell | Email: kcamlolo668@gmail.com | Date completed: 9 Sep 2019 |
| --- | --- | --- | --- |
|  |  |  |  |

Do you wish to be acknowledged? (If yes, the review team will be advised to add an acknowledgement to any publications related to this work). No – unless your organization requires it

The suggested acknowledgement is “We thank Xxxxx Yyyyyy, MLIS, AHIP (xxxxx Health Sciences Library, University of xxxxxx) for peer review of the MEDLINE search strategy.” [please edit to indicate your name, postnomials and institutional affiliation as you would like them presented].

|  | **1. TRANSLATION** |  | | |  |
| --- | --- | --- | --- | --- | --- |
| A -­‐No revisions | X |  |  |  |  |
| B -­‐ Revision(s) suggested |  |  |  |  |  |
| C -­‐ Revision(s) required |  |  |  |  |  |

If “B” or “C,” please provide an explanation or example:

**2. BOOLEAN AND PROXIMITY OPERATORS**

| A -­‐No revisions | X |
| --- | --- |
| B -­‐ Revision(s) suggested |  |
| C -­‐ Revision(s) required |  |

If “B” or “C,” please provide an explanation or example:

**3. SUBJECT HEADINGS**

| A -­‐No revisions | X |
| --- | --- |
| B -­‐ Revision(s) suggested |  |
| C -­‐ Revision(s) required |  |

If “B” or “C,” please provide an explanation or example:

**4. TEXT WORD SEARCHING**

| A -­‐No revisions | X |
| --- | --- |
| B -­‐ Revision(s)suggested |  |
| C -­‐ Revision(s) required |  |

If “B” or “C,” please provide an explanation or example:

**5. SPELLING, SYNTAX, AND LINE NUMBERS**

| A -­‐No revisions | X |
| --- | --- |
| B -­‐ Revision(s)suggested |  |
| C -­‐ Revision(s) required |  |

If “B” or “C,” please provide an explanation or example:

**6. LIMITS AND FILTERS**

| A -­‐No revisions | X |
| --- | --- |
| B -­‐ Revision(s) suggested |  |
| C -­‐ Revision(s) required |  |

If “B” or “C,” please provide an explanation or example:

OVERALL EVALUATION (Note: If one or more “revision required” is noted above, the response below must be “revisions required”.)

| A -­‐No revisions | X |
| --- | --- |
| B -­‐ Revision(s) suggested |  |
| C -­‐ Revision(s) required |  |

Additional comments:

Nicely done, no errors or omissions detected. I noted that you’re using Create Date (DT) as the year limit field. Recently, I’ve been using Entry Date (ED) OR’d with the year limit (for Medline only--ED not helpful for Embase b/c of the record reload issue). Thoughts?
