# Supplementary material for: Screening for depression in children and adolescents in primary care or non-mental health settings: a systematic review update
Source: Syst Rev. 2024 Jan 31;13:48. doi: 10.1186/s13643-023-02447-3 (PMC10829174; doi:10.1186/s13643-023-02447-3)
Supplement: Supplementary file 4 — Additional file 4. Database search strategies. [file 13643_2023_2447_MOESM4_ESM.docx]

## Additional file 4: Database search strategies

### RCT search strategies

2021 Feb 19 – Updated from 2019 Nov 4

Ovid Multifile

Database: Embase Classic+Embase <1947 to 2021 February 17>, Ovid MEDLINE(R) ALL <1946 to February 17, 2021>, APA PsycInfo <1806 to February Week 2 2021>, EBM Reviews - Cochrane Central Register of Controlled Trials <January 2021>

Search Strategy:

--------------------------------------------------------------------------------

1 exp Depressive Disorder/ (633226)

2 Depression/ (553755)

3 depress*.tw,kf. (1565738)

4 dysthym*.tw,kf. (12132)

5 blues.tw,kf. (5113)

6 melanchol*.tw,kf. (12805)

7 MDD.tw,kf. (50577)

8 or/1-7 [GENERAL DEPRESSION] (1790848)

9 Mass Screening/ (171323)

10 (screen* or detect*).tw,kf. (7492103)

11 (identif* or recogni*).ti. (952534)

12 ((early or earlier or earliest) adj5 (identif* or recogni*)).tw,kf. (207770)

13 (case finding? or casefinding?).tw,kf. (14851)

14 or/9-13 [GENERAL SCREENING] (8406870)

15 8 and 14 (188172)

16 Adolescent/ (3891844)

17 Child/ (3831063)

18 Minors/ (3307)

19 (boy or boys or girl or girls or child* or school-age* or adolescen* or teen or teens or teenage* or youth or youths or highschool* or high-school* or juvenil* or pubescen* or under?age*).tw,kf. (5558960)

20 p?ediatric*.tw,kf. (1051870)

21 or/16-20 [CHILD/ADOLESCENT POPULATION] (8985312)

22 15 and 21 [SCREENING - DEPRESSION - CHILD/ADOLESCENTS] (38386)

23 exp Animals/ not Humans/ (19341365)

24 22 not 23 [ANIMAL-ONLY REMOVED] (29779)

25 (comment or editorial or news or newspaper article).pt. (2177634)

26 (letter not (randomized controlled trial and letter)).pt. (2303844)

27 24 not (25 or 26) [OPINION PIECES REMOVED] (29647)

28 (controlled clinical trial or randomized controlled trial or pragmatic clinical trial or equivalence trial).pt. (1211858)

29 clinical trials as topic/ (309642)

30 exp Randomized Controlled Trials as Topic/ (349221)

31 (randomi#ation? or randomi#ed or randomly or RCT or placebo*).tw,kf. (3781844)

32 ((singl* or doubl* or trebl* or tripl*) adj (mask* or blind* or dumm*)).tw,kf. (746751)

33 trial.ti. (943103)

34 or/28-33 (4725129)

35 27 and 34 [RCTs] (4331)

36 (201911* or 201912* or 2020* or 2021*).dt. (1997609)

37 35 and 36 (160)

38 limit 35 to yr="2017-current" (1549)

39 (201911* or 201912* or 2020* or 2021*).ed. (1315287)

40 38 and 39 (139)

41 37 or 40 [UPDATE PERIOD] (233)

42 41 use medall [MEDLINE RECORDS] (233)

43 exp Depressive Disorder/ (633226)

44 depress*.tw,kw. (1579410)

45 dysthym*.tw,kw. (12493)

46 blues.tw,kw. (5135)

47 melanchol*.tw,kw. (12923)

48 MDD.tw,kw. (50751)

49 or/43-48 [GENERAL DEPRESSION] (1776681)

50 screening/ (304629)

51 mass screening/ (171323)

52 screening test/ (81257)

53 (screen* or detect*).tw,kw. (7506465)

54 (identif* or recogni*).ti. (952534)

55 ((early or earlier or earliest) adj5 (identif* or recogni*)).tw,kw. (207896)

56 (case finding? or casefinding?).tw,kw. (15010)

57 or/50-56 (8452654)

58 depression assessment/ (2168)

59 49 and 57 (189954)

60 58 or 59 [DEPRESSION & SCREENING/ASSESSMENT] (191652)

61 adolescent/ (3891844)

62 child/ (3831063)

63 school child/ (381428)

64 "minor (person)"/ (686)

65 (boy or boys or girl or girls or child* or school-age* or adolescen* or teen or teens or teenage* or youth or youths or highschool* or high-school* or juvenil* or pubescen* or under?age*).tw,kw. (5586488)

66 p?ediatric*.tw,kw. (1081541)

67 or/61-66 [CHILD/ADOLESCENT POPULATION] (9037787)

68 60 and 67 [DEPRESSION - SCREENING - CHILD/ADOLESCENT POPULATION] (39144)

69 exp animal/ or exp animal experimentation/ or exp animal model/ or exp animal experiment/ or nonhuman/ or exp vertebrate/ (55669622)

70 exp human/ or exp human experimentation/ or exp human experiment/ (43074605)

71 69 not 70 (12596853)

72 68 not 71 [ANIMAL-ONLY REMOVED] (38666)

73 editorial.pt. (1248834)

74 letter.pt. not (randomized controlled trial/ and letter.pt.) (2303527)

75 72 not (73 or 74) [OPINION PIECES REMOVED] (38495)

76 randomized controlled trial/ or controlled clinical trial/ (1454681)

77 "clinical trial (topic)"/ (111199)

78 exp "controlled clinical trial (topic)"/ (205119)

79 (randomi#ation? or randomi#ed or randomly or RCT or placebo*).tw,kw. (3844140)

80 ((singl* or doubl* or trebl* or tripl*) adj (mask* or blind* or dumm*)).tw,kw. (775166)

81 trial.ti. (943103)

82 or/76-81 (4717008)

83 75 and 82 [RCTs] (5323)

84 (201911* or 201912* or 2020* or 2021*).dc. (2833319)

85 83 and 84 [UPDATE PERIOD] (236)

86 85 use emczd [EMBASE RECORDS] (236)

87 "Depression (Emotion)"/ (162769)

88 exp Major Depression/ (203830)

89 depress*.tw. (1559162)

90 dysthym*.tw. (12099)

91 blues.tw. (5102)

92 melanchol*.tw. (12766)

93 MDD.tw. (50430)

94 or/87-93 [GENERAL DEPRESSION] (1611387)

95 Screening/ (304629)

96 exp Screening Tests/ (35377)

97 exp Health Screening/ (276099)

98 (screen* or detect*).tw. (7482123)

99 (identif* or recogni*).ti. (952534)

100 ((early or earlier or earliest) adj5 (identif* or recogni*)).tw. (207652)

101 (case finding? or casefinding?).tw. (14759)

102 or/95-101 [GENERAL SCREENING] (8493113)

103 94 and 102 [DEPRESSION - SCREENING] (177786)

104 (boy or boys or girl or girls or child* or school-age* or adolescen* or teen or teens or teenage* or youth or youths or highschool* or high-school* or juvenil* or pubescen* or under?age*).tw. (5506740)

105 p?ediatric*.tw. (1034118)

106 104 or 105 (5895880)

107 103 and 106 (28507)

108 limit 103 to (180 school age <age 6 to 12 yrs> or 200 adolescence <age 13 to 17 yrs>) [Limit not valid in Embase,Ovid MEDLINE(R),Ovid MEDLINE(R) Daily Update,Ovid MEDLINE(R) In-Process,Ovid MEDLINE(R) Publisher,CCTR; records were retained] (153398)

109 107 or 108 [DEPRESSION - SCREENING -CHILD/ADOLESCENT POPULATION] (156452)

110 Clinical Trials/ (109246)

111 (randomi#ation? or randomi#ed or randomly or RCT or placebo*).tw. (3779157)

112 ((singl* or doubl* or trebl* or tripl*) adj (mask* or blind* or dumm*)).tw. (746627)

113 trial.ti. (943103)

114 or/110-113 (4144058)

115 109 and 114 [RCTs] (22750)

116 (201910* or 201911* or 201912* or 2020* or 2021*).up. (37313218)

117 115 and 116 [UPDATE PERIOD] (12613)

118 117 use medall,emczd,cctr (12550)

119 117 not 118 [PSYCINFO RECORDS] (63)

120 exp Depressive Disorder/ (633226)

121 Depression/ (553755)

122 depress*.ti,ab,kw. (1570771)

123 dysthym*.ti,ab,kw. (12303)

124 blues.ti,ab,kw. (5057)

125 melanchol*.ti,ab,kw. (12677)

126 MDD.ti,ab,kw. (50655)

127 or/120-126 [GENERAL DEPRESSION] (1792694)

128 Mass Screening/ (171323)

129 (screen* or detect*).ti,ab,kw. (7502182)

130 (identif* or recogni*).ti. (952534)

131 ((early or earlier or earliest) adj5 (identif* or recogni*)).ti,ab,kw. (207594)

132 (case finding? or casefinding?).ti,ab,kw. (14982)

133 or/128-132 [GENERAL SCREENING] (8416493)

134 127 and 133 (188827)

135 Adolescent/ (3891844)

136 Child/ (3831063)

137 Minors/ (3307)

138 (boy or boys or girl or girls or child* or school-age* or adolescen* or teen or teens or teenage* or youth or youths or highschool* or high-school* or juvenil* or pubescen* or under?age*).ti,ab,kw. (5555489)

139 p?ediatric*.ti,ab,kw. (1077498)

140 or/135-139 [CHILD/ADOLESCENT POPULATION] (8983393)

141 134 and 140 [SCREENING - DEPRESSION - CHILD/ADOLESCENTS] (38438)

142 (201910* or 201911* or 201912* or 2020* or 2021*).up. (37313218)

143 141 and 142 [UPDATE PERIOD] (18029)

144 143 use cctr [CENTRAL RECORDS] (1604)

145 42 or 86 or 119 or 144 [ALL DATABASES] (2136)

146 remove duplicates from 145 (1850) [TOTAL UNIQUE RECORDS]

147 146 use medall [MEDLINE UNIQUE RECORDS - UPDATE PERIOD] (221)

148 146 use emczd [EMBASE UNIQUE RECORDS - UPDATE PERIOD] (105)

149 146 use cctr (1502)

150 146 not (147 or 148 or 149) [PSYCINFO UNIQUE RECORDS - UPDATE PERIOD] (22)

151 146 use cctr [CENTRAL UNIQUE RECORDS - UPDATE PERIOD] (1502)

***************************

CINAHL

| # | Query | Limiters/Expanders | Last Run Via | Results |
| --- | --- | --- | --- | --- |
| S30 | S28 AND S29 | Expanders - Apply related words; Apply equivalent subjects  Search modes - Boolean/Phrase | Interface - EBSCOhost Research Databases  Search Screen - Advanced Search  Database - CINAHL | 69 |
| S29 | EM 201911-2021 | Expanders - Apply related words; Apply equivalent subjects  Search modes - Boolean/Phrase | Interface - EBSCOhost Research Databases  Search Screen - Advanced Search  Database - CINAHL | 470,386 |
| S28 | S21 AND S27 | Expanders - Apply related words  Search modes - Boolean/Phrase | Interface - EBSCOhost Research Databases  Search Screen - Advanced Search  Database - CINAHL | 571 |
| S27 | S22 OR S23 OR S24 OR S25 OR S26 | Expanders - Apply related words  Search modes - Boolean/Phrase | Interface - EBSCOhost Research Databases  Search Screen - Advanced Search  Database - CINAHL | 1,089,248 |
| S26 | TI ( pediatric* or paediatric* ) OR AB ( pediatric* or paediatric* ) | Expanders - Apply related words  Search modes - Boolean/Phrase | Interface - EBSCOhost Research Databases  Search Screen - Advanced Search  Database - CINAHL | 142,308 |
| S25 | TI ( boy or boys or girl or girls or child* or (school w0 age*) or adolescen* or teen or teens or teenage* or (teen W0 age*) or youth or youths or highschool* or (high W0 school*) or juvenil* or pubescen* or underage* or (under W0 age*) ) OR AB ( boy or boys or girl or girls or child* or (school w0 age*) or adolescen* or teen or teens or teenage* or (teen W0 age*) or youth or youths or highschool* or (high W0 school*) or juvenil* or pubescen* or underage* or (under W0 age*) ) | Expanders - Apply related words  Search modes - Boolean/Phrase | Interface - EBSCOhost Research Databases  Search Screen - Advanced Search  Database - CINAHL | 656,570 |
| S24 | (MH "Minors (Legal)") | Expanders - Apply related words  Search modes - Boolean/Phrase | Interface - EBSCOhost Research Databases  Search Screen - Advanced Search  Database - CINAHL | 726 |
| S23 | (MH "Child") | Expanders - Apply related words  Search modes - Boolean/Phrase | Interface - EBSCOhost Research Databases  Search Screen - Advanced Search  Database - CINAHL | 471,280 |
| S22 | (MH "Adolescence+") | Expanders - Apply related words  Search modes - Boolean/Phrase | Interface - EBSCOhost Research Databases  Search Screen - Advanced Search  Database - CINAHL | 541,038 |
| S21 | S14 AND S20 | Expanders - Apply related words  Search modes - Boolean/Phrase | Interface - EBSCOhost Research Databases  Search Screen - Advanced Search  Database - CINAHL | 2,551 |
| S20 | S15 OR S16 OR S17 OR S18 OR S19 | Expanders - Apply related words  Search modes - Boolean/Phrase | Interface - EBSCOhost Research Databases  Search Screen - Advanced Search  Database - CINAHL | 398,377 |
| S19 | TI trial | Expanders - Apply related words  Search modes - Boolean/Phrase | Interface - EBSCOhost Research Databases  Search Screen - Advanced Search  Database - CINAHL | 114,499 |
| S18 | TI ( ((singl* or doubl* or trebl* or tripl*) N1 (mask* or blind* or dumm*)) ) OR AB ( ((singl* or doubl* or trebl* or tripl*) N1 (mask* or blind* or dumm*)) ) | Expanders - Apply related words  Search modes - Boolean/Phrase | Interface - EBSCOhost Research Databases  Search Screen - Advanced Search  Database - CINAHL | 50,860 |
| S17 | TI ( randomi?ed or randomly or RCT or RCTs or placebo* ) OR AB ( randomi?ed or randomly or RCT or RCTs or placebo* ) | Expanders - Apply related words  Search modes - Boolean/Phrase | Interface - EBSCOhost Research Databases  Search Screen - Advanced Search  Database - CINAHL | 336,630 |
| S16 | (MH "Double-Blind Studies") OR (MH "Single-Blind Studies") OR (MH "Triple-Blind Studies") | Expanders - Apply related words  Search modes - Boolean/Phrase | Interface - EBSCOhost Research Databases  Search Screen - Advanced Search  Database - CINAHL | 64,471 |
| S15 | (MH "Randomized Controlled Trials") | Expanders - Apply related words  Search modes - Boolean/Phrase | Interface - EBSCOhost Research Databases  Search Screen - Advanced Search  Database - CINAHL | 112,870 |
| S14 | S7 AND S13 | Expanders - Apply related words  Search modes - Boolean/Phrase | Interface - EBSCOhost Research Databases  Search Screen - Advanced Search  Database - CINAHL | 22,069 |
| S13 | S8 OR S9 OR S10 OR S11 OR S12 | Expanders - Apply related words  Search modes - Boolean/Phrase | Interface - EBSCOhost Research Databases  Search Screen - Advanced Search  Database - CINAHL | 473,369 |
| S12 | TI ( "case finding" or "case findings" or casefinding or casefindings ) OR AB ( "case finding" or "case findings" or casefinding or casefindings ) | Expanders - Apply related words  Search modes - Boolean/Phrase | Interface - EBSCOhost Research Databases  Search Screen - Advanced Search  Database - CINAHL | 1,586 |
| S11 | TI ( ((early or earlier or earliest) N5 (identif* or recogni*)) ) OR AB ( ((early or earlier or earliest) N5 (identif* or recogni*)) ) | Expanders - Apply related words  Search modes - Boolean/Phrase | Interface - EBSCOhost Research Databases  Search Screen - Advanced Search  Database - CINAHL | 22,922 |
| S10 | TI (identif* or recogni*) | Expanders - Apply related words  Search modes - Boolean/Phrase | Interface - EBSCOhost Research Databases  Search Screen - Advanced Search  Database - CINAHL | 63,127 |
| S9 | TI ( (screen* or detect*) ) OR AB ( (screen* or detect*) ) | Expanders - Apply related words  Search modes - Boolean/Phrase | Interface - EBSCOhost Research Databases  Search Screen - Advanced Search  Database - CINAHL | 387,358 |
| S8 | (MH "Health Screening") | Expanders - Apply related words  Search modes - Boolean/Phrase | Interface - EBSCOhost Research Databases  Search Screen - Advanced Search  Database - CINAHL | 48,380 |
| S7 | S1 OR S2 OR S3 OR S4 OR S5 OR S6 | Expanders - Apply related words  Search modes - Boolean/Phrase | Interface - EBSCOhost Research Databases  Search Screen - Advanced Search  Database - CINAHL | 191,165 |
| S6 | TI MDD OR AB MDD | Expanders - Apply related words  Search modes - Boolean/Phrase | Interface - EBSCOhost Research Databases  Search Screen - Advanced Search  Database - CINAHL | 3,998 |
| S5 | TI melanchol* OR AB melanchol* | Expanders - Apply related words  Search modes - Boolean/Phrase | Interface - EBSCOhost Research Databases  Search Screen - Advanced Search  Database - CINAHL | 504 |
| S4 | TI blues OR AB blues | Expanders - Apply related words  Search modes - Boolean/Phrase | Interface - EBSCOhost Research Databases  Search Screen - Advanced Search  Database - CINAHL | 9,582 |
| S3 | TI dysthym* OR AB dysthym* | Expanders - Apply related words  Search modes - Boolean/Phrase | Interface - EBSCOhost Research Databases  Search Screen - Advanced Search  Database - CINAHL | 803 |
| S2 | TI depress* OR AB depress* | Expanders - Apply related words  Search modes - Boolean/Phrase | Interface - EBSCOhost Research Databases  Search Screen - Advanced Search  Database - CINAHL | 152,680 |
| S1 | (MH "Depression+") | Expanders - Apply related words  Search modes - Boolean/Phrase | Interface - EBSCOhost Research Databases  Search Screen - Advanced Search  Database - CINAHL | 116,624 |

### Non-randomized controlled studies search strategies

Depression – Adolescents – nRCTs

Update

2021 Feb 22

*Addendum: Recovers 2021 records not ID’d in 2021 Feb 14 search*

Ovid Multifile

Database: Embase Classic+Embase <1947 to 2021 February 19>, Ovid MEDLINE(R) ALL <1946 to February 19, 2021>, APA PsycInfo <1806 to February Week 3 2021>

Search Strategy:

--------------------------------------------------------------------------------

1 exp Depressive Disorder/ (621600)

2 Depression/ (542039)

3 depress*.tw,kf. (1482317)

4 dysthym*.tw,kf. (11443)

5 blues.tw,kf. (4953)

6 melanchol*.tw,kf. (12440)

7 MDD.tw,kf. (46557)

8 or/1-7 [GENERAL DEPRESSION] (1705388)

9 Mass Screening/ (168223)

10 (screen* or detect*).tw,kf. (7326319)

11 (identif* or recogni*).ti. (944612)

12 ((early or earlier or earliest) adj5 (identif* or recogni*)).tw,kf. (203465)

13 (case finding? or casefinding?).tw,kf. (14367)

14 or/9-13 [GENERAL SCREENING] (8231550)

15 8 and 14 (178475)

16 Adolescent/ (3788619)

17 Child/ (3785318)

18 Minors/ (3300)

19 (boy or boys or girl or girls or child* or school-age* or adolescen* or teen or teens or teenage* or youth or youths or highschool* or high-school* or juvenil* or pubescen* or under?age*).tw,kf. (5393948)

20 p?ediatric*.tw,kf. (1015323)

21 or/16-20 [CHILD/ADOLESCENT POPULATION] (8727500)

22 15 and 21 [SCREENING - DEPRESSION - CHILD/ADOLESCENTS] (36475)

23 exp Animals/ not Humans/ (19386887)

24 22 not 23 [ANIMAL-ONLY REMOVED] (27812)

25 (comment or editorial or news or newspaper article).pt. (2171939)

26 (letter not (randomized controlled trial and letter)).pt. (2300318)

27 24 not (25 or 26) [OPINION PIECES REMOVED] (27680)

28 controlled clinical trial.pt. (94078)

29 Controlled Clinical Trials as Topic/ (15762)

30 (control* adj2 trial).tw,kf. (428538)

31 Non-Randomized Controlled Trials as Topic/ (12360)

32 (nonrandom* or non-random* or quasi-random* or quasi-experiment*).tw,kf. (155174)

33 (nRCT or nonRCT or non-RCT).tw,kf. (934)

34 Controlled Before-After Studies/ (225701)

35 (control* adj3 ("before and after" or "before after")).tw,kf. (11491)

36 Control Groups/ (125899)

37 (control* adj2 group?).tw,kf. (1399530)

38 trial.ti. (600321)

39 Comparative Study.pt. (1883211)

40 ((comparative or comparison) adj (study or studies)).tw,kf. (288036)

41 or/28-40 [STUDY DESIGN FILTER] (4475027)

42 27 and 41 (3187)

43 (2015* or 2016* or 2017* or 2018* or 2019* or 2020* or 2021*).dt. (7705426)

44 (2015* or 2016* or 2017* or 2018* or 2019* or 2020* or 2021*).dp. (10072768)

45 42 and 43 (619)

46 42 and 44 (891)

47 45 or 46 [DATE LIMITS APPLIED] (894)

48 47 use medall [MEDLINE RECORDS] (638)

49 exp Depressive Disorder/ (621600)

50 depress*.tw,kw. (1491553)

51 dysthym*.tw,kw. (11553)

52 blues.tw,kw. (4975)

53 melanchol*.tw,kw. (12540)

54 MDD.tw,kw. (46732)

55 or/49-54 [GENERAL DEPRESSION] (1687860)

56 screening/ (304806)

57 mass screening/ (168223)

58 screening test/ (81356)

59 (screen* or detect*).tw,kw. (7337841)

60 (identif* or recogni*).ti. (944612)

61 ((early or earlier or earliest) adj5 (identif* or recogni*)).tw,kw. (203592)

62 (case finding? or casefinding?).tw,kw. (14515)

63 or/56-62 (8274574)

64 depression assessment/ (2172)

65 55 and 63 (179874)

66 64 or 65 [DEPRESSION & SCREENING/ASSESSMENT] (181575)

67 adolescent/ (3788619)

68 child/ (3785318)

69 school child/ (382005)

70 "minor (person)"/ (687)

71 (boy or boys or girl or girls or child* or school-age* or adolescen* or teen or teens or teenage* or youth or youths or highschool* or high-school* or juvenil* or pubescen* or under?age*).tw,kw. (5399208)

72 p?ediatric*.tw,kw. (1043956)

73 or/67-72 [CHILD/ADOLESCENT POPULATION] (8763637)

74 66 and 73 [DEPRESSION - SCREENING - CHILD/ADOLESCENT POPULATION] (37119)

75 exp animal/ or exp animal experimentation/ or exp animal model/ or exp animal experiment/ or nonhuman/ or exp vertebrate/ (55111200)

76 exp human/ or exp human experimentation/ or exp human experiment/ (42507844)

77 75 not 76 (12605193)

78 74 not 77 [ANIMAL-ONLY REMOVED] (36641)

79 editorial.pt. (1247209)

80 letter.pt. not (randomized controlled trial/ and letter.pt.) (2294611)

81 78 not (79 or 80) [OPINION PIECES REMOVED] (36469)

82 controlled clinical trial/ (564387)

83 "controlled clinical trial (topic)"/ (11496)

84 (control* adj2 trial).tw,kw. (432747)

85 (nonrandom* or non-random* or quasi-random* or quasi-experiment*).tw,kw. (155461)

86 (nRCT or nonRCT or non-RCT).tw,kw. (936)

87 (control* adj3 ("before and after" or "before after")).tw,kw. (11495)

88 control group/ (125899)

89 (control* adj2 group?).tw,kw. (1399292)

90 trial.ti. (600321)

91 comparative study/ (2811937)

92 ((comparative or comparison) adj (study or studies)).tw,kw. (286477)

93 or/82-92 [STUDY DESIGN FILTER] (5368845)

94 81 and 93 (4447)

95 (2015* or 2016* or 2017* or 2018* or 2019* or 2020* or 2021*).dc. (11209315)

96 (2015* or 2016* or 2017* or 2018* or 2019* or 2020* or 2021*).dp. (10072768)

97 94 and 95 (1040)

98 94 and 96 (970)

99 97 or 98 [DATE LIMITS APPLIED] (1876)

100 99 use emczd [EMBASE RECORDS] (1040)

101 "Depression (Emotion)"/ (150514)

102 exp Major Depression/ (204135)

103 depress*.tw. (1475775)

104 dysthym*.tw. (11409)

105 blues.tw. (4942)

106 melanchol*.tw. (12402)

107 MDD.tw. (46414)

108 or/101-107 [GENERAL DEPRESSION] (1526301)

109 Screening/ (304806)

110 exp Screening Tests/ (34205)

111 exp Health Screening/ (276405)

112 (screen* or detect*).tw. (7316485)

113 (identif* or recogni*).ti. (944612)

114 ((early or earlier or earliest) adj5 (identif* or recogni*)).tw. (203349)

115 (case finding? or casefinding?).tw. (14277)

116 or/109-115 [GENERAL SCREENING] (8318405)

117 108 and 116 [DEPRESSION - SCREENING] (168145)

118 (boy or boys or girl or girls or child* or school-age* or adolescen* or teen or teens or teenage* or youth or youths or highschool* or high-school* or juvenil* or pubescen* or under?age*).tw. (5341845)

119 p?ediatric*.tw. (997785)

120 118 or 119 (5719729)

121 117 and 120 (26989)

122 limit 117 to (180 school age <age 6 to 12 yrs> or 200 adolescence <age 13 to 17 yrs>) [Limit not valid in Embase,Ovid MEDLINE(R),Ovid MEDLINE(R) Daily Update,Ovid MEDLINE(R) PubMed not MEDLINE,Ovid MEDLINE(R) In-Process,Ovid MEDLINE(R) Publisher; records were retained] (143704)

123 121 or 122 [DEPRESSION - SCREENING -CHILD/ADOLESCENT POPULATION] (146766)

124 (control* adj2 trial).tw. (424405)

125 (nonrandom* or non-random* or quasi-random* or quasi-experiment*).tw. (154958)

126 (nRCT or nonRCT or non-RCT).tw. (932)

127 (control* adj3 ("before and after" or "before after")).tw. (11478)

128 (control* adj2 group?).tw. (1399157)

129 Experiment Controls/ (926)

130 trial.ti. (600321)

131 ((comparative or comparison) adj (study or studies)).tw. (283134)

132 or/124-131 [STUDY DESIGN FILTER] (2458960)

133 123 and 132 (12425)

134 (2015* or 2016* or 2017* or 2018* or 2019* or 2020* or 2021*).up. (71361158)

135 (2015* or 2016* or 2017* or 2018* or 2019* or 2020* or 2021*).dp. (10072768)

136 133 and 134 (11339)

137 133 and 135 (2954)

138 136 or 137 [DATE LIMITS APPLIED] (11342)

139 138 use medall,emczd (11112)

140 138 not 139 [PSYCINFO RECORDS] (230)

141 48 or 100 or 140 [ALL DATABASES] (1908)

142 remove duplicates from 141 (1327)

143 142 use medall [MEDLINE RECORDS] (635)

144 2021*.dt. (227265)

145 143 and 144 [MEDLINE UNIQUE RECORDS - 2021 UPDATE PERIOD] (10)

146 142 use emczd [EMBASE RECORDS] (627)

147 2021*.dc. (391682)

148 146 and 147 [EMBASE UNIQUE RECORDS - 2021 UPDATE PERIOD] (17)

149 142 not (143 or 146) [PSYCINFO RECORDS] (65)

150 2021*.up. (1760088)

151 149 and 150 [PSYCINFO UNIQUE RECORDS - 2021 UPDATE PERIOD] (4)

152 145 or 148 or 151 [TOTAL UNIQUE RECORDS - ALL DATABASES - 2021 UPDATE PERIOD] (31)

***************************

CINAHL

| # | Query | Limiters/Expanders | Last Run Via | Results |
| --- | --- | --- | --- | --- |
| S39 | S37 AND S38 | Expanders - Apply related words; Apply equivalent subjects  Search modes - Boolean/Phrase | Interface - EBSCOhost Research Databases  Search Screen - Advanced Search  Database - CINAHL | 38 |
| S38 | EM 2021- | Expanders - Apply related words; Apply equivalent subjects  Search modes - Boolean/Phrase | Interface - EBSCOhost Research Databases  Search Screen - Advanced Search  Database - CINAHL | 54,451 |
| S37 | S35 AND S36 | Expanders - Apply related words; Apply equivalent subjects  Search modes - Boolean/Phrase | Interface - EBSCOhost Research Databases  Search Screen - Advanced Search  Database - CINAHL | 700 |
| S36 |  | Limiters - Published Date: 20150101-20211231  Expanders - Apply related words; Apply equivalent subjects  Search modes - Boolean/Phrase | Interface - EBSCOhost Research Databases  Search Screen - Advanced Search  Database - CINAHL | 2,687,846 |
| S35 | S21 AND S34 | Expanders - Apply related words; Apply equivalent subjects  Search modes - Boolean/Phrase | Interface - EBSCOhost Research Databases  Search Screen - Advanced Search  Database - CINAHL | 1,085 |
| S34 | S22 OR S23 OR S24 OR S25 OR S26 OR S27 OR S28 OR S29 OR S30 OR S31 OR S32 OR S33 | Expanders - Apply related words; Apply equivalent subjects  Search modes - Boolean/Phrase | Interface - EBSCOhost Research Databases  Search Screen - Advanced Search  Database - CINAHL | 725,247 |
| S33 | TI ( (comparative or comparison) W0 (study or studies) ) OR AB ( (comparative or comparison) W0 (study or studies) ) | Expanders - Apply related words; Apply equivalent subjects  Search modes - Boolean/Phrase | Interface - EBSCOhost Research Databases  Search Screen - Advanced Search  Database - CINAHL | 17,204 |
| S32 | (MH "Comparative Studies") | Expanders - Apply related words; Apply equivalent subjects  Search modes - Boolean/Phrase | Interface - EBSCOhost Research Databases  Search Screen - Advanced Search  Database - CINAHL | 360,696 |
| S31 | TI trial | Expanders - Apply related words; Apply equivalent subjects  Search modes - Boolean/Phrase | Interface - EBSCOhost Research Databases  Search Screen - Advanced Search  Database - CINAHL | 114,588 |
| S30 | TI control* N2 group# OR AB control* N2 group# | Expanders - Apply related words; Apply equivalent subjects  Search modes - Boolean/Phrase | Interface - EBSCOhost Research Databases  Search Screen - Advanced Search  Database - CINAHL | 123,067 |
| S29 | (MH "Control Group") | Expanders - Apply related words; Apply equivalent subjects  Search modes - Boolean/Phrase | Interface - EBSCOhost Research Databases  Search Screen - Advanced Search  Database - CINAHL | 12,423 |
| S28 | TI ( control* N3 ("before and after" or "before after") ) OR AB ( control* N3 ("before and after" or "before after") ) | Expanders - Apply related words; Apply equivalent subjects  Search modes - Boolean/Phrase | Interface - EBSCOhost Research Databases  Search Screen - Advanced Search  Database - CINAHL | 1,826 |
| S27 | (MH "Controlled Before-After Studies") | Expanders - Apply related words; Apply equivalent subjects  Search modes - Boolean/Phrase | Interface - EBSCOhost Research Databases  Search Screen - Advanced Search  Database - CINAHL | 196 |
| S26 | TI ( nRCT or nonRCT or "non-RCT" ) OR AB ( nRCT or nonRCT or "non-RCT" ) | Expanders - Apply related words; Apply equivalent subjects  Search modes - Boolean/Phrase | Interface - EBSCOhost Research Databases  Search Screen - Advanced Search  Database - CINAHL | 173 |
| S25 | TI ( nonrandom* or (non W0 random*) or (quasi W0 random*) or (quasi W0 experiment*) ) OR AB ( nonrandom* or (non W0 random*) or (quasi W0 random*) or (quasi W0 experiment*) ) | Expanders - Apply related words; Apply equivalent subjects  Search modes - Boolean/Phrase | Interface - EBSCOhost Research Databases  Search Screen - Advanced Search  Database - CINAHL | 23,272 |
| S24 | (MH "Nonrandomized Trials") | Expanders - Apply related words; Apply equivalent subjects  Search modes - Boolean/Phrase | Interface - EBSCOhost Research Databases  Search Screen - Advanced Search  Database - CINAHL | 608 |
| S23 | TI control* N2 trial OR AB control* N2 trial | Expanders - Apply related words; Apply equivalent subjects  Search modes - Boolean/Phrase | Interface - EBSCOhost Research Databases  Search Screen - Advanced Search  Database - CINAHL | 133,380 |
| S22 | (MH "Clinical Trials") | Expanders - Apply related words; Apply equivalent subjects  Search modes - Boolean/Phrase | Interface - EBSCOhost Research Databases  Search Screen - Advanced Search  Database - CINAHL | 174,637 |
| S21 | S14 AND S20 | Expanders - Apply related words; Apply equivalent subjects  Search modes - Boolean/Phrase | Interface - EBSCOhost Research Databases  Search Screen - Advanced Search  Database - CINAHL | 5,877 |
| S20 | S15 OR S16 OR S17 OR S18 OR S19 | Expanders - Apply related words; Apply equivalent subjects  Search modes - Boolean/Phrase | Interface - EBSCOhost Research Databases  Search Screen - Advanced Search  Database - CINAHL | 1,089,629 |
| S19 | TI ( pediatric* or paediatric* ) OR AB ( pediatric* or paediatric* ) | Expanders - Apply related words; Apply equivalent subjects  Search modes - Boolean/Phrase | Interface - EBSCOhost Research Databases  Search Screen - Advanced Search  Database - CINAHL | 142,401 |
| S18 | TI ( boy or boys or girl or girls or child* or (school w0 age*) or adolescen* or teen or teens or teenage* or (teen W0 age*) or youth or youths or highschool* or (high W0 school*) or juvenil* or pubescen* or underage* or (under W0 age*) ) OR AB ( boy or boys or girl or girls or child* or (school w0 age*) or adolescen* or teen or teens or teenage* or (teen W0 age*) or youth or youths or highschool* or (high W0 school*) or juvenil* or pubescen* or underage* or (under W0 age*) ) | Expanders - Apply related words; Apply equivalent subjects  Search modes - Boolean/Phrase | Interface - EBSCOhost Research Databases  Search Screen - Advanced Search  Database - CINAHL | 656,872 |
| S17 | (MH "Minors (Legal)") | Expanders - Apply related words; Apply equivalent subjects  Search modes - Boolean/Phrase | Interface - EBSCOhost Research Databases  Search Screen - Advanced Search  Database - CINAHL | 726 |
| S16 | (MH "Child") | Expanders - Apply related words; Apply equivalent subjects  Search modes - Boolean/Phrase | Interface - EBSCOhost Research Databases  Search Screen - Advanced Search  Database - CINAHL | 471,380 |
| S15 | (MH "Adolescence+") | Expanders - Apply related words; Apply equivalent subjects  Search modes - Boolean/Phrase | Interface - EBSCOhost Research Databases  Search Screen - Advanced Search  Database - CINAHL | 541,122 |
| S14 | S7 AND S13 | Expanders - Apply related words; Apply equivalent subjects  Search modes - Boolean/Phrase | Interface - EBSCOhost Research Databases  Search Screen - Advanced Search  Database - CINAHL | 22,083 |
| S13 | S8 OR S9 OR S10 OR S11 OR S12 | Expanders - Apply related words; Apply equivalent subjects  Search modes - Boolean/Phrase | Interface - EBSCOhost Research Databases  Search Screen - Advanced Search  Database - CINAHL | 473,569 |
| S12 | TI ( "case finding" or "case findings" or casefinding or casefindings ) OR AB ( "case finding" or "case findings" or casefinding or casefindings ) | Expanders - Apply related words; Apply equivalent subjects  Search modes - Boolean/Phrase | Interface - EBSCOhost Research Databases  Search Screen - Advanced Search  Database - CINAHL | 1,587 |
| S11 | TI ( (early or earlier or earliest) N5 (identif* or recogni*) ) OR AB ( (early or earlier or earliest) N5 (identif* or recogni*) ) | Expanders - Apply related words; Apply equivalent subjects  Search modes - Boolean/Phrase | Interface - EBSCOhost Research Databases  Search Screen - Advanced Search  Database - CINAHL | 22,929 |
| S10 | TI (identif* or recogni*) | Expanders - Apply related words; Apply equivalent subjects  Search modes - Boolean/Phrase | Interface - EBSCOhost Research Databases  Search Screen - Advanced Search  Database - CINAHL | 63,161 |
| S9 | TI ( screen* or detect* ) OR AB ( screen* or detect* ) | Expanders - Apply related words; Apply equivalent subjects  Search modes - Boolean/Phrase | Interface - EBSCOhost Research Databases  Search Screen - Advanced Search  Database - CINAHL | 387,521 |
| S8 | (MH "Health Screening") | Expanders - Apply related words; Apply equivalent subjects  Search modes - Boolean/Phrase | Interface - EBSCOhost Research Databases  Search Screen - Advanced Search  Database - CINAHL | 48,400 |
| S7 | S1 OR S2 OR S3 OR S4 OR S5 OR S6 | Expanders - Apply related words; Apply equivalent subjects  Search modes - Boolean/Phrase | Interface - EBSCOhost Research Databases  Search Screen - Advanced Search  Database - CINAHL | 191,238 |
| S6 | TI MDD OR AB MDD | Expanders - Apply related words; Apply equivalent subjects  Search modes - Boolean/Phrase | Interface - EBSCOhost Research Databases  Search Screen - Advanced Search  Database - CINAHL | 3,999 |
| S5 | TI melanchol* OR AB melanchol* | Expanders - Apply related words; Apply equivalent subjects  Search modes - Boolean/Phrase | Interface - EBSCOhost Research Databases  Search Screen - Advanced Search  Database - CINAHL | 504 |
| S4 | TI blues OR AB blues | Expanders - Apply related words; Apply equivalent subjects  Search modes - Boolean/Phrase | Interface - EBSCOhost Research Databases  Search Screen - Advanced Search  Database - CINAHL | 9,583 |
| S3 | TI dysthym* OR AB dysthym* | Expanders - Apply related words; Apply equivalent subjects  Search modes - Boolean/Phrase | Interface - EBSCOhost Research Databases  Search Screen - Advanced Search  Database - CINAHL | 803 |
| S2 | TI depress* OR AB depress* | Expanders - Apply related words; Apply equivalent subjects  Search modes - Boolean/Phrase | Interface - EBSCOhost Research Databases  Search Screen - Advanced Search  Database - CINAHL | 152,748 |
| S1 | (MH "Depression+") | Expanders - Apply related words; Apply equivalent subjects  Search modes - Boolean/Phrase | Interface - EBSCOhost Research Databases  Search Screen - Advanced Search  Database - CINAHL | 116,669 |

2021 Feb 14 – Updated from 2020 Sep 27

Ovid Multifile

Database: Embase Classic+Embase <1947 to 2021 February 12>, Ovid MEDLINE(R) ALL <1946 to February 12, 2021>, APA PsycInfo <1806 to February Week 2 2021>

Search Strategy:

--------------------------------------------------------------------------------

1 exp Depressive Disorder/ (620390)

2 Depression/ (540793)

3 depress*.tw,kf. (1482767)

4 dysthym*.tw,kf. (11444)

5 blues.tw,kf. (4958)

6 melanchol*.tw,kf. (12445)

7 MDD.tw,kf. (46684)

8 or/1-7 [GENERAL DEPRESSION] (1705573)

9 Mass Screening/ (168016)

10 (screen* or detect*).tw,kf. (7329609)

11 (identif* or recogni*).ti. (945354)

12 ((early or earlier or earliest) adj5 (identif* or recogni*)).tw,kf. (203486)

13 (case finding? or casefinding?).tw,kf. (14380)

14 or/9-13 [GENERAL SCREENING] (8235390)

15 8 and 14 (178481)

16 Adolescent/ (3784185)

17 Child/ (3780093)

18 Minors/ (3297)

19 (boy or boys or girl or girls or child* or school-age* or adolescen* or teen or teens or teenage* or youth or youths or highschool* or high-school* or juvenil* or pubescen* or under?age*).tw,kf. (5394853)

20 p?ediatric*.tw,kf. (1015455)

21 or/16-20 [CHILD/ADOLESCENT POPULATION] (8725932)

22 15 and 21 [SCREENING - DEPRESSION - CHILD/ADOLESCENTS] (36446)

23 exp Animals/ not Humans/ (19338447)

24 22 not 23 [ANIMAL-ONLY REMOVED] (27839)

25 (comment or editorial or news or newspaper article).pt. (2173448)

26 (letter not (randomized controlled trial and letter)).pt. (2300487)

27 24 not (25 or 26) [OPINION PIECES REMOVED] (27707)

28 controlled clinical trial.pt. (94063)

29 Controlled Clinical Trials as Topic/ (15739)

30 (control* adj2 trial).tw,kf. (429148)

31 Non-Randomized Controlled Trials as Topic/ (12331)

32 (nonrandom* or non-random* or quasi-random* or quasi-experiment*).tw,kf. (155295)

33 (nRCT or nonRCT or non-RCT).tw,kf. (931)

34 Controlled Before-After Studies/ (225298)

35 (control* adj3 ("before and after" or "before after")).tw,kf. (11497)

36 Control Groups/ (125887)

37 (control* adj2 group?).tw,kf. (1399645)

38 trial.ti. (601263)

39 Comparative Study.pt. (1882534)

40 ((comparative or comparison) adj (study or studies)).tw,kf. (288043)

41 or/28-40 [STUDY DESIGN FILTER] (4475253)

42 27 and 41 (3194)

43 (2015* or 2016* or 2017* or 2018* or 2019* or 2020*).dt. (7593870)

44 (2015* or 2016* or 2017* or 2018* or 2019* or 2020*).dp. (9821999)

45 42 and 43 (619)

46 42 and 44 (886)

47 45 or 46 [DATE LIMITS APPLIED] (895)

48 47 use medall [MEDLINE RECORDS] (639)

49 exp Depressive Disorder/ (620390)

50 depress*.tw,kw. (1491968)

51 dysthym*.tw,kw. (11554)

52 blues.tw,kw. (4980)

53 melanchol*.tw,kw. (12545)

54 MDD.tw,kw. (46858)

55 or/49-54 [GENERAL DEPRESSION] (1688031)

56 screening/ (304554)

57 mass screening/ (168016)

58 screening test/ (81237)

59 (screen* or detect*).tw,kw. (7341009)

60 (identif* or recogni*).ti. (945354)

61 ((early or earlier or earliest) adj5 (identif* or recogni*)).tw,kw. (203610)

62 (case finding? or casefinding?).tw,kw. (14528)

63 or/56-62 (8278248)

64 depression assessment/ (2166)

65 55 and 63 (179873)

66 64 or 65 [DEPRESSION & SCREENING/ASSESSMENT] (181569)

67 adolescent/ (3784185)

68 child/ (3780093)

69 school child/ (381268)

70 "minor (person)"/ (686)

71 (boy or boys or girl or girls or child* or school-age* or adolescen* or teen or teens or teenage* or youth or youths or highschool* or high-school* or juvenil* or pubescen* or under?age*).tw,kw. (5400041)

72 p?ediatric*.tw,kw. (1044004)

73 or/67-72 [CHILD/ADOLESCENT POPULATION] (8761979)

74 66 and 73 [DEPRESSION - SCREENING - CHILD/ADOLESCENT POPULATION] (37088)

75 exp animal/ or exp animal experimentation/ or exp animal model/ or exp animal experiment/ or nonhuman/ or exp vertebrate/ (55041619)

76 exp human/ or exp human experimentation/ or exp human experiment/ (42450169)

77 75 not 76 (12593286)

78 74 not 77 [ANIMAL-ONLY REMOVED] (36610)

79 editorial.pt. (1247555)

80 letter.pt. not (randomized controlled trial/ and letter.pt.) (2294790)

81 78 not (79 or 80) [OPINION PIECES REMOVED] (36439)

82 controlled clinical trial/ (564010)

83 "controlled clinical trial (topic)"/ (11473)

84 (control* adj2 trial).tw,kw. (433292)

85 (nonrandom* or non-random* or quasi-random* or quasi-experiment*).tw,kw. (155581)

86 (nRCT or nonRCT or non-RCT).tw,kw. (933)

87 (control* adj3 ("before and after" or "before after")).tw,kw. (11501)

88 control group/ (125887)

89 (control* adj2 group?).tw,kw. (1399408)

90 trial.ti. (601263)

91 comparative study/ (2810302)

92 ((comparative or comparison) adj (study or studies)).tw,kw. (286478)

93 or/82-92 [STUDY DESIGN FILTER] (5368374)

94 81 and 93 (4447)

95 (2015* or 2016* or 2017* or 2018* or 2019* or 2020*).dc. (10820071)

96 (2015* or 2016* or 2017* or 2018* or 2019* or 2020*).dp. (9821999)

97 94 and 95 (1006)

98 94 and 96 (964)

99 97 or 98 [DATE LIMITS APPLIED] (1840)

100 99 use emczd [EMBASE RECORDS] (1009)

101 "Depression (Emotion)"/ (149957)

102 exp Major Depression/ (203796)

103 depress*.tw. (1476203)

104 dysthym*.tw. (11411)

105 blues.tw. (4947)

106 melanchol*.tw. (12406)

107 MDD.tw. (46536)

108 or/101-107 [GENERAL DEPRESSION] (1526707)

109 Screening/ (304554)

110 exp Screening Tests/ (34080)

111 exp Health Screening/ (275965)

112 (screen* or detect*).tw. (7319655)

113 (identif* or recogni*).ti. (945354)

114 ((early or earlier or earliest) adj5 (identif* or recogni*)).tw. (203368)

115 (case finding? or casefinding?).tw. (14288)

116 or/109-115 [GENERAL SCREENING] (8321951)

117 108 and 116 [DEPRESSION - SCREENING] (168164)

118 (boy or boys or girl or girls or child* or school-age* or adolescen* or teen or teens or teenage* or youth or youths or highschool* or high-school* or juvenil* or pubescen* or under?age*).tw. (5342661)

119 p?ediatric*.tw. (997745)

120 118 or 119 (5720580)

121 117 and 120 (26979)

122 limit 117 to (180 school age <age 6 to 12 yrs> or 200 adolescence <age 13 to 17 yrs>) [Limit not valid in Embase,Ovid MEDLINE(R),Ovid MEDLINE(R) Daily Update,Ovid MEDLINE(R) In-Process,Ovid MEDLINE(R) Publisher; records were retained] (143776)

123 121 or 122 [DEPRESSION - SCREENING -CHILD/ADOLESCENT POPULATION] (146830)

124 (control* adj2 trial).tw. (424961)

125 (nonrandom* or non-random* or quasi-random* or quasi-experiment*).tw. (155075)

126 (nRCT or nonRCT or non-RCT).tw. (929)

127 (control* adj3 ("before and after" or "before after")).tw. (11484)

128 (control* adj2 group?).tw. (1399273)

129 Experiment Controls/ (925)

130 trial.ti. (601263)

131 ((comparative or comparison) adj (study or studies)).tw. (283138)

132 or/124-131 [STUDY DESIGN FILTER] (2460141)

133 123 and 132 (12452)

134 (2015* or 2016* or 2017* or 2018* or 2019* or 2020*).up. (70213341)

135 (2015* or 2016* or 2017* or 2018* or 2019* or 2020*).dp. (9821999)

136 133 and 134 (11003)

137 133 and 135 (2908)

138 136 or 137 [DATE LIMITS APPLIED] (11148)

139 138 use medall,emczd (10918)

140 138 not 139 [PSYCINFO RECORDS] (230)

141 48 or 100 or 140 [ALL DATABASES] (1878)

142 remove duplicates from 141 (1302)

143 142 use medall [MEDLINE RECORDS] (624)

144 (2020092* or 202010* or 202011* or 202012* or 2021*).dt. (637994)

145 143 and 144 [MEDLINE UNIQUE RECORDS - UPDATE PERIOD] (21)

146 142 use emczd [EMBASE RECORDS] (612)

147 (2020092* or 202010* or 202011* or 202012* or 2021*).dc. (1155579)

148 146 and 147 [EMBASE UNIQUE RECORDS - UPDATE PERIOD] (38)

149 142 not (143 or 146) [PSYCINFO RECORDS] (66)

150 (202009* or 202010* or 202011* or 202012* or 2021*).up. (4019165)

151 149 and 150 [PSYCINFO UNIQUE RECORDS - UPDATE PERIOD] (6)

152 145 or 148 or 151 [TOTAL UNIQUE RECORDS - ALL DATABASES - UPDATE PERIOD] (65)

***************************

CINAHL

| # | Query | Limiters/Expanders | Last Run Via | Results |
| --- | --- | --- | --- | --- |
| S39 | S37 AND S38 | Expanders - Apply related words; Apply equivalent subjects  Search modes - Boolean/Phrase | Interface - EBSCOhost Research Databases  Search Screen - Advanced Search  Database - CINAHL | 41 |
| S38 | EM 202009- | Expanders - Apply related words; Apply equivalent subjects  Search modes - Boolean/Phrase | Interface - EBSCOhost Research Databases  Search Screen - Advanced Search  Database - CINAHL | 171,951 |
| S37 | S35 AND S36 | Expanders - Apply related words; Apply equivalent subjects  Search modes - Boolean/Phrase | Interface - EBSCOhost Research Databases  Search Screen - Advanced Search  Database - CINAHL | 664 |
| S36 |  | Limiters - Published Date: 20150101-20201231  Expanders - Apply related words; Apply equivalent subjects  Search modes - Boolean/Phrase | Interface - EBSCOhost Research Databases  Search Screen - Advanced Search  Database - CINAHL | 2,624,646 |
| S35 | S21 AND S34 | Expanders - Apply related words; Apply equivalent subjects  Search modes - Boolean/Phrase | Interface - EBSCOhost Research Databases  Search Screen - Advanced Search  Database - CINAHL | 1,061 |
| S34 | S22 OR S23 OR S24 OR S25 OR S26 OR S27 OR S28 OR S29 OR S30 OR S31 OR S32 OR S33 | Expanders - Apply related words; Apply equivalent subjects  Search modes - Boolean/Phrase | Interface - EBSCOhost Research Databases  Search Screen - Advanced Search  Database - CINAHL | 722,886 |
| S33 | TI ( (comparative or comparison) W0 (study or studies) ) OR AB ( (comparative or comparison) W0 (study or studies) ) | Expanders - Apply related words; Apply equivalent subjects  Search modes - Boolean/Phrase | Interface - EBSCOhost Research Databases  Search Screen - Advanced Search  Database - CINAHL | 17,173 |
| S32 | (MH "Comparative Studies") | Expanders - Apply related words; Apply equivalent subjects  Search modes - Boolean/Phrase | Interface - EBSCOhost Research Databases  Search Screen - Advanced Search  Database - CINAHL | 358,662 |
| S31 | TI trial | Expanders - Apply related words; Apply equivalent subjects  Search modes - Boolean/Phrase | Interface - EBSCOhost Research Databases  Search Screen - Advanced Search  Database - CINAHL | 114,342 |
| S30 | TI control* N2 group# OR AB control* N2 group# | Expanders - Apply related words; Apply equivalent subjects  Search modes - Boolean/Phrase | Interface - EBSCOhost Research Databases  Search Screen - Advanced Search  Database - CINAHL | 122,863 |
| S29 | (MH "Control Group") | Expanders - Apply related words; Apply equivalent subjects  Search modes - Boolean/Phrase | Interface - EBSCOhost Research Databases  Search Screen - Advanced Search  Database - CINAHL | 12,412 |
| S28 | TI ( control* N3 ("before and after" or "before after") ) OR AB ( control* N3 ("before and after" or "before after") ) | Expanders - Apply related words; Apply equivalent subjects  Search modes - Boolean/Phrase | Interface - EBSCOhost Research Databases  Search Screen - Advanced Search  Database - CINAHL | 1,826 |
| S27 | (MH "Controlled Before-After Studies") | Expanders - Apply related words; Apply equivalent subjects  Search modes - Boolean/Phrase | Interface - EBSCOhost Research Databases  Search Screen - Advanced Search  Database - CINAHL | 195 |
| S26 | TI ( nRCT or nonRCT or "non-RCT" ) OR AB ( nRCT or nonRCT or "non-RCT" ) | Expanders - Apply related words; Apply equivalent subjects  Search modes - Boolean/Phrase | Interface - EBSCOhost Research Databases  Search Screen - Advanced Search  Database - CINAHL | 172 |
| S25 | TI ( nonrandom* or (non W0 random*) or (quasi W0 random*) or (quasi W0 experiment*) ) OR AB ( nonrandom* or (non W0 random*) or (quasi W0 random*) or (quasi W0 experiment*) ) | Expanders - Apply related words; Apply equivalent subjects  Search modes - Boolean/Phrase | Interface - EBSCOhost Research Databases  Search Screen - Advanced Search  Database - CINAHL | 23,223 |
| S24 | (MH "Nonrandomized Trials") | Expanders - Apply related words; Apply equivalent subjects  Search modes - Boolean/Phrase | Interface - EBSCOhost Research Databases  Search Screen - Advanced Search  Database - CINAHL | 605 |
| S23 | TI control* N2 trial OR AB control* N2 trial | Expanders - Apply related words; Apply equivalent subjects  Search modes - Boolean/Phrase | Interface - EBSCOhost Research Databases  Search Screen - Advanced Search  Database - CINAHL | 133,121 |
| S22 | (MH "Clinical Trials") | Expanders - Apply related words; Apply equivalent subjects  Search modes - Boolean/Phrase | Interface - EBSCOhost Research Databases  Search Screen - Advanced Search  Database - CINAHL | 174,449 |
| S21 | S14 AND S20 | Expanders - Apply related words; Apply equivalent subjects  Search modes - Boolean/Phrase | Interface - EBSCOhost Research Databases  Search Screen - Advanced Search  Database - CINAHL | 5,863 |
| S20 | S15 OR S16 OR S17 OR S18 OR S19 | Expanders - Apply related words; Apply equivalent subjects  Search modes - Boolean/Phrase | Interface - EBSCOhost Research Databases  Search Screen - Advanced Search  Database - CINAHL | 1,087,812 |
| S19 | TI ( pediatric* or paediatric* ) OR AB ( pediatric* or paediatric* ) | Expanders - Apply related words; Apply equivalent subjects  Search modes - Boolean/Phrase | Interface - EBSCOhost Research Databases  Search Screen - Advanced Search  Database - CINAHL | 142,123 |
| S18 | TI ( boy or boys or girl or girls or child* or (school w0 age*) or adolescen* or teen or teens or teenage* or (teen W0 age*) or youth or youths or highschool* or (high W0 school*) or juvenil* or pubescen* or underage* or (under W0 age*) ) OR AB ( boy or boys or girl or girls or child* or (school w0 age*) or adolescen* or teen or teens or teenage* or (teen W0 age*) or youth or youths or highschool* or (high W0 school*) or juvenil* or pubescen* or underage* or (under W0 age*) ) | Expanders - Apply related words; Apply equivalent subjects  Search modes - Boolean/Phrase | Interface - EBSCOhost Research Databases  Search Screen - Advanced Search  Database - CINAHL | 655,632 |
| S17 | (MH "Minors (Legal)") | Expanders - Apply related words; Apply equivalent subjects  Search modes - Boolean/Phrase | Interface - EBSCOhost Research Databases  Search Screen - Advanced Search  Database - CINAHL | 725 |
| S16 | (MH "Child") | Expanders - Apply related words; Apply equivalent subjects  Search modes - Boolean/Phrase | Interface - EBSCOhost Research Databases  Search Screen - Advanced Search  Database - CINAHL | 470,575 |
| S15 | (MH "Adolescence+") | Expanders - Apply related words; Apply equivalent subjects  Search modes - Boolean/Phrase | Interface - EBSCOhost Research Databases  Search Screen - Advanced Search  Database - CINAHL | 540,115 |
| S14 | S7 AND S13 | Expanders - Apply related words; Apply equivalent subjects  Search modes - Boolean/Phrase | Interface - EBSCOhost Research Databases  Search Screen - Advanced Search  Database - CINAHL | 22,037 |
| S13 | S8 OR S9 OR S10 OR S11 OR S12 | Expanders - Apply related words; Apply equivalent subjects  Search modes - Boolean/Phrase | Interface - EBSCOhost Research Databases  Search Screen - Advanced Search  Database - CINAHL | 472,536 |
| S12 | TI ( "case finding" or "case findings" or casefinding or casefindings ) OR AB ( "case finding" or "case findings" or casefinding or casefindings ) | Expanders - Apply related words; Apply equivalent subjects  Search modes - Boolean/Phrase | Interface - EBSCOhost Research Databases  Search Screen - Advanced Search  Database - CINAHL | 1,586 |
| S11 | TI ( (early or earlier or earliest) N5 (identif* or recogni*) ) OR AB ( (early or earlier or earliest) N5 (identif* or recogni*) ) | Expanders - Apply related words; Apply equivalent subjects  Search modes - Boolean/Phrase | Interface - EBSCOhost Research Databases  Search Screen - Advanced Search  Database - CINAHL | 22,876 |
| S10 | TI (identif* or recogni*) | Expanders - Apply related words; Apply equivalent subjects  Search modes - Boolean/Phrase | Interface - EBSCOhost Research Databases  Search Screen - Advanced Search  Database - CINAHL | 63,004 |
| S9 | TI ( screen* or detect* ) OR AB ( screen* or detect* ) | Expanders - Apply related words; Apply equivalent subjects  Search modes - Boolean/Phrase | Interface - EBSCOhost Research Databases  Search Screen - Advanced Search  Database - CINAHL | 386,677 |
| S8 | (MH "Health Screening") | Expanders - Apply related words; Apply equivalent subjects  Search modes - Boolean/Phrase | Interface - EBSCOhost Research Databases  Search Screen - Advanced Search  Database - CINAHL | 48,302 |
| S7 | S1 OR S2 OR S3 OR S4 OR S5 OR S6 | Expanders - Apply related words; Apply equivalent subjects  Search modes - Boolean/Phrase | Interface - EBSCOhost Research Databases  Search Screen - Advanced Search  Database - CINAHL | 190,843 |
| S6 | TI MDD OR AB MDD | Expanders - Apply related words; Apply equivalent subjects  Search modes - Boolean/Phrase | Interface - EBSCOhost Research Databases  Search Screen - Advanced Search  Database - CINAHL | 3,996 |
| S5 | TI melanchol* OR AB melanchol* | Expanders - Apply related words; Apply equivalent subjects  Search modes - Boolean/Phrase | Interface - EBSCOhost Research Databases  Search Screen - Advanced Search  Database - CINAHL | 502 |
| S4 | TI blues OR AB blues | Expanders - Apply related words; Apply equivalent subjects  Search modes - Boolean/Phrase | Interface - EBSCOhost Research Databases  Search Screen - Advanced Search  Database - CINAHL | 9,572 |
| S3 | TI dysthym* OR AB dysthym* | Expanders - Apply related words; Apply equivalent subjects  Search modes - Boolean/Phrase | Interface - EBSCOhost Research Databases  Search Screen - Advanced Search  Database - CINAHL | 803 |
| S2 | TI depress* OR AB depress* | Expanders - Apply related words; Apply equivalent subjects  Search modes - Boolean/Phrase | Interface - EBSCOhost Research Databases  Search Screen - Advanced Search  Database - CINAHL | 152,394 |
| S1 | (MH "Depression+") | Expanders - Apply related words; Apply equivalent subjects  Search modes - Boolean/Phrase | Interface - EBSCOhost Research Databases  Search Screen - Advanced Search  Database - CINAHL | 116,024 |
|  |  |  |  |  |
