# Supplementary material for: Screening for depression in children and adolescents in primary care or non-mental health settings: a systematic review update
Source: Syst Rev. 2024 Jan 31;13:48. doi: 10.1186/s13643-023-02447-3 (PMC10829174; doi:10.1186/s13643-023-02447-3)
Supplement: Supplementary file 5 — Additional file 5. List of websites searched (grey literature search). [file 13643_2023_2447_MOESM5_ESM.docx]

## Additional file 5: List of websites searched

- American Academy of Child and Adolescent Psychiatry: www.aacap.org
- American Academy of Family Physicians: www.aafp.org
- American Academy of Pediatrics: www.aap.org
- American College of Physicians: www.acponline.org
- American Nurses Association: www.nursingworld.org
- American Psychological Association: www.apa.org
- Anxiety and Depression Association of America: www.adaa.org
- Canadian Academy of Child and Adolescent Psychiatry: www.cacap-acpea.org
- Canadian Mental Health Association: www.cmha.ca
- Canadian Nurses Association: www.cna-aiic.ca
- Canadian Paediatric Society: www.cps.ca
- Canadian Psychiatric Association: www.cpa-apc.org
- Centre for Addiction and Mental Health: www.camh.ca
- Child Mind Institute: www.childmind.org
- College of Family Physicians of Canada: www.cfp.ca
