# Supplementary material for: Screening for depression in children and adolescents in primary care or non-mental health settings: a systematic review update
Source: Syst Rev. 2024 Jan 31;13:48. doi: 10.1186/s13643-023-02447-3 (PMC10829174; doi:10.1186/s13643-023-02447-3)
Supplement: Supplementary file 6 — Additional file 6. List of excluded studies with reasons (randomized controlled trials). [file 13643_2023_2447_MOESM6_ESM.docx]

## Additional file 6: List of excluded studies with reasons (RCT)

### Adult population (n=21)

1. ACTRN12619001433190 (2019) Perinatal Identification, Referral and Integrated Management for Improving Depression: the PIRIMID Study. who.int/trialsearch/Trial2.aspx?TrialID=ACTRN12619001433190. 2019.:
2. assessment of depression and mood during pregnancy: protocol of a feasibility study. BMJ open 2017. 7 (5) e014469-.
3. Carlin E, Blondell SJ, Cadet-James Y, et al (2019) Study protocol: a clinical trial for improving mental health screening for Aboriginal and Torres Strait Islander pregnant women and mothers of young children using the Kimberley Mum’s Mood Scale. BMC public health 19:1521, 2019
4. CTRI/2020/10/028770 (2020) Effect of delivery of an Integrated intervention package during Pre-pregnancy, Pregnancy and Early Childhood on infant growth markers in the first 6 months of life. who.int/trialsearch/Trial2.aspx?TrialID=CTRI/2020/10/028770. 2020.:
5. Du, Na, Yu, Kexin, Ye, Yan, and Chen, Shulin. Validity study of Patient Health Questionnaire-9 items for Internet screening in depression among Chinese university students. Asia-Pacific psychiatry : official journal of the Pacific Rim College of Psychiatrists 2017. 9 (3) -.
6. ISRCTN42298046. The effectiveness of screening for postpartum depression in child health care. http://www.who.int/trialsearch/Trial2.aspx?TrialID=ISRCTN42298046 2016. () –
7. Jin, Haomiao and Wu, Shinyi. Screening Depression and Related Conditions via Text Messaging Versus Interview Assessment: Protocol for a Randomized Study. JMIR research protocols 2019. 8 (3) e12392-.
8. Kendig, S., Keats, J. P., Camille, Hoffman M., Kay, L. B., Miller, E. S., Simas, T. A. M., Frieder, A., Hackley, B., Indman, P., Raines, C., Semenuk, K., Wisner, K. L., and Lemieux, L. A.. Consensus bundle on maternal mental health perinatal depression and anxiety. Obstetrics and Gynecology 2017. 129 (3) 422-430.
9. Kingston, Dawn, Biringer, Anne, Veldhuyzen van Zanten, Sander, Giallo, Rebecca, McDonald, Sarah, MacQueen, Glenda, Vermeyden, Lydia, and Austin, Marie Paule. Pregnant Women's Perceptions of the Risks and Benefits of Disclosure During Web-Based Mental Health E-Screening Versus Paper-Based Screening: Randomized Controlled Trial. JMIR mental health 2017. 4 (4) e42-.
10. Marcano Belisario, Jose Salvador, Doherty, Kevin, O'Donoghue, John, Ramchandani, Paul, Majeed, Azeem, Doherty, Gavin, Morrison, Cecily, and Car, Josip. A bespoke mobile application for the longitudinal
11. Marley JV, Kotz J.. Validity and acceptability of Kimberley Mum's mood scale to screen for perinatal anxiety and depression in remote aboriginal health care settings. PloS one 2017. 12 (1) (no pagination) -.
12. McKean, Michelle, Caughey, Aaron B., Yuracko McKean, Melanie A., Cabana, Michael D., and Flaherman, Valerie J.. Postpartum Depression: When Should Health Care Providers Identify Those at Risk?. Clinical pediatrics 2018. 57 (6) 689-693.
13. NCT00819702. A Safe Environment for Every Kid (SEEK): a Model for Primary Care. https://clinicaltrials.gov/show/NCT00819702 2009. () -.
14. NCT01901796 (2013) Integrated Maternal Psychosocial Assessment to Care Trial (IMPACT) Full Trial. gov/show/NCT01901796. 2013.:
15. PACTR201303000485383. Developing a screening tool for postnatal Depression in Zimbabwe. http://www.who.int/trialsearch/Trial2.aspx?TrialID=PACTR201303000485383 2013. () .
16. Panattoni, Laura, Hurlimann, Lily, Wilson, Caroline, Durbin, Meg, and Tai-Seale, Ming. Workflow standardization of a novel team care model to improve chronic care: a quasi-experimental study. BMC health services research 2017. 17 (1) 286-.
17. Poleshuck E, Wittink M, Crean HF, Juskiewicz I, Bell E, Harrington A, Cerulli C (2020) A Comparative Effectiveness Trial of Two Patient-Centered Interventions for Women with Unmet Social Needs: personalized Support for Progress and Enhanced Screening and Referral. 29:242–252
18. Rona, Roberto J., Burdett, Howard, Khondoker, Mizanur, Chesnokov, Melanie, Green, Kevin, Pernet, David, Jones, Norman, Greenberg, Neil, Wessely, Simon, and Fear, Nicola T.. Post-deployment screening for mental disorders and tailored advice about help-seeking in the UK military: a cluster randomised controlled trial. Lancet (London, England) 2017. 389 (10077) 1410-1423.
19. Sun Y, Fu Z, Bo Q, Mao Z, Ma X, Wang C. The reliability and validity of PHQ-9 in patients with major depressive disorder in psychiatric hospital. BMC psychiatry. 2020;20(1).
20. Tachibana Y, Koizumi N, Mikami M, Shikada K, Yamashita S, Shimizu M, Machida K, Ito H (2020) An integrated community mental healthcare program to reduce suicidal ideation and improve maternal mental health during the postnatal period: The findings from the Nagano trial. BMC psychiatry 20:
21. Wajid, Abdul, van Zanten, Sander Veldhuyzen, Mughal, Muhammad Kashif, Biringer, Anne, Austin, Marie Paule, Vermeyden, Lydia, and Kingston, Dawn. Adversity in childhood and depression in pregnancy. Archives of women's mental health 2019. () -.

### Does not meet the depression screening trial criteria (n=6)

1. Guo, Sisi, Kim, Joanna J., Bear, Laurel, and Lau, Anna S.. Does Depression Screening in Schools Reduce Adolescent Racial/Ethnic Disparities in Accessing Treatment?. Journal of clinical child and adolescent psychology : the official journal for the Society of Clinical Child and Adolescent Psychology, American Psychological Association, Division 53 2017. 46 (4) 523-536.
2. Mahoney, Nicholas, Gladstone, Tracy, DeFrino, Daniela, Stinson, Allison, Nidetz, Jennifer, Canel, Jason, Ching, Eumene, Berry, Anita, Cantorna, James, Fogel, Joshua, Eder, Milton, Bolotin, Megan, and Van Voorhees, Benjamin W.. Prevention of Adolescent Depression in Primary Care: Barriers and Relational Work Solutions. Californian journal of health promotion 2017. 15 (2) 1-12.
3. Mirzaie, Parvin, Nazarian, Lida, and Zare, Hossein. Validation of the Maria Kovacs Children's Depression Inventory to Assess Depression among School Children in Afghanistan. Journal of Midwifery & Reproductive Health 2019. 7 (3) 1742-1748.
4. Rinke ML, Bundy DG, Stein REK, O’Donnell HC, Heo M, Sangvai S, Lilienfeld H, Singh H (2019) Increasing Recognition and Diagnosis of Adolescent Depression: Project RedDE: A Cluster Randomized Trial. Pediatr Qual Saf 4:e217, 2019-e217, 2Oct
5. Sterling, Stacy, Kline-Simon, Andrea H., Weisner, Constance, Jones, Ashley, and Satre, Derek D.. Pediatrician and Behavioral Clinician-Delivered Screening, Brief Intervention and Referral to Treatment: Substance Use and Depression Outcomes. The Journal of adolescent health: official publication of the Society for Adolescent Medicine 2018. 62 (4) 390-396.
6. Thabrew H, D’Silva S, Darragh M, Goldfinch M, Meads J, Goodyear-Smith F (2019) Comparison of YouthCHAT, an Electronic Composite Psychosocial Screener, With a Clinician Interview Assessment for Young People: Randomized Controlled Trial. Journal of medical Internet research 21:e13911

### Not relevant to screening (n=16)

1. Bansa, Melishia, Brown, Darryl, DeFrino, Daniela, Mahoney, Nicholas, Saulsberry, Alexandria, Marko-Holguin, Monika, Fogel, Joshua, Gladstone, Tracy R. G., and Van Voorhees, Benjamin W.. A Little Effort Can Withstand the Hardship: Fielding an Internet-Based Intervention to Prevent Depression among Urban Racial/Ethnic Minority Adolescents in a Primary Care Setting. Journal of the National Medical Association 2018. 110 (2) 130-142.
2. Barrera M, Alexander S, Atenafu EG, Chung J, Hancock K, Solomon A, et al. Psychosocial screening and mental health in pediatric cancer: A randomized controlled trial. Health Psychol. 2020;39(5):381–90.
3. Blossom JB, Adrian MC, Stoep AV, McCauley E. Mechanisms of Change in the Prevention of Depression: An Indicated School-Based Prevention Trial at the Transition to High School. Journal of the American Academy of Child and Adolescent Psychiatry. 2020;59(4):541–51.
4. Byatt, Nancy, Moore Simas, Tiffany A., Biebel, Kathleen, Sankaran, Padma, Pbert, Lori, Weinreb, Linda, Ziedonis, Douglas, and Allison, Jeroan. PRogram In Support of Moms (PRISM): a pilot group randomized controlled trial of two approaches to improving depression among perinatal women. Journal of psychosomatic obstetrics and gynaecology 2018. 39 (4) 297-306.
5. de Jonge-Heesen KWJ, Rasing SPA, Vermulst AA, Scholte RHJ, van Ettekoven KM, Engels RCME, et al. Randomized control trial testing the effectiveness of implemented depression prevention in high-risk adolescents. BMC medicine. 2020;18(1):188, 2020.
6. Depression (Children and Adolescents): Exercise. Joanna Briggs Institute 2019. () -.
7. Do R, Lee S, Kim J-S, Cho M, Shin H, Jang M, et al. Effectiveness and dissemination of computer-based cognitive behavioral therapy for depressed adolescents: Effective and accessible to whom? Journal of affective disorders. 2021;282:885–93.
8. Ede, Moses Onyemaechi, Igbo, Janet N., Eseadi, Chiedu, Ede, Kelechi R., Ezegbe, Bernedeth N., Ede, Augustina O., Ezurike, Chukwuemeka, Onwuka, Gloria T., and Ali, Rifkatu B.. Effect of group cognitive behavioural therapy on depressive symptoms in a sample of college adolescents in nigeria. Journal of Rational-Emotive & Cognitive-Behavior Therapy 2019. () No-Specified.
9. Felder, Jennifer N., Epel, Elissa, Lewis, Jessica B., Cunningham, Shayna D., Tobin, Jonathan N., Rising, Sharon Schindler, Thomas, Melanie, and Ickovics, Jeannette R.. Depressive symptoms and gestational length among pregnant adolescents: Cluster randomized control trial of CenteringPregnancy plus group prenatal care. Journal of consulting and clinical psychology 2017. 85 (6) 574-584.
10. Haddad, Mark, Pinfold, Vanessa, Ford, Tamsin, Walsh, Brendan, and Tylee, Andre. The effect of a training programme on school nurses' knowledge, attitudes, and depression recognition skills: The QUEST cluster randomised controlled trial. International journal of nursing studies 2018. 83 () 1-10.
11. Knapp AA, Feldner M, Allan NP, Schmidt NB, Keough ME, Leen-Feldner EW. Test of an Anxiety Sensitivity Amelioration Program for at-risk youth (ASAP-Y). Behaviour research and therapy. 2020;126:103544.
12. Parthasarathy S, Kline-Simon AH, Jones A, Hartman L, Saba K, Weisner C, et al. Three-Year Outcomes After Brief Treatment of Substance Use and Mood Symptoms. Pediatrics. 2021;147(1).
13. Schleider JL, Dobias M, Fassler J, Shroff A, Pati S. Promoting treatment access following pediatric primary care depression screening: Randomized trial of web-based, single-session interventions for parents and youths. Journal of the American Academy of Child and Adolescent Psychiatry. 2020;59(6):770–3.
14. Sebastianski, Meghan, Gates, Michelle, Gates, Allison, Nuspl, Megan, Bialy, Liza M., Featherstone, Robin M., Breault, Lorraine, Mason-Lai, Ping, and Hartling, Lisa. Evidence available for patient-identified priorities in depression research: results of 11 rapid responses. BMJ open 2019. 9 (6) e026847-.
15. Smith, Justin D., Berkel, Cady, Hails, Katherine A., Dishion, Thomas J., Shaw, Daniel S., and Wilson, Melvin N.. Predictors of Participation in the Family Check-Up Program: a Randomized Trial of Yearly Services from Age 2 to 10 Years. Prevention science : the official journal of the Society for Prevention Research 2018. 19 (5) 652-662.
16. Van Buren, Dorothy J., Wilfley, Denise E., Marcus, Marsha D., Anderson, Barbara, Abramson, Natalie Walders, Berkowitz, Robert, Ievers-Landis, Carolyn, Trief, Paula, Yasuda, Patrice, Hirst, Kathryn, and TODAY Study Group. Depressive symptoms and glycemic control in youth with type 2 diabetes participating in the TODAY clinical trial. Diabetes research and clinical practice 2018. 135 () 85-87.

### Protocol, study registration (n=42)

1. ACTRN12619000914167. Evaluation of the effectiveness of Coping with Accident REactions (CARE) screen and-treat early intervention for improving recovery from trauma reactions in young injured children and their parents. http://www.who.int/trialsearch/Trial2.aspx?TrialID=ACTRN12619000914167 2019. () –
2. AM Kilbourne, SN Smith, SY Choi, E Koschmann, C Liebrecht, A Rusch, JL Abelson, D Eisenberg, JA Himle, K Fitzgerald, D Almirall. Adaptive School-based Implementation of CBT (ASIC): clustered-SMART for building an optimized adaptive implementation intervention to improve uptake of mental health interventions in schools.. 2018. 13 (1) -.
3. Baldofski, Sabrina, Kohls, Elisabeth, Bauer, Stephanie, Becker, Katja, Bilic, Sally, Eschenbeck, Heike, Kaess, Michael, Moessner, Markus, Salize, Hans Joachim, Diestelkamp, Silke, Vos, Elke, Rummel-Kluge, Christine, and ProHEAD consortium. Efficacy and cost-effectiveness of two online interventions for children and adolescents at risk for depression (E.motion trial): study protocol for a randomized controlled trial within the ProHEAD consortium. Trials 2019. 20 (1) 53-.
4. Beck A, LeBlanc JC, Morissette K, Hamel C, Skidmore B, Colquhoun H, et al. Screening for depression in children and adolescents: a protocol for a systematic review update. Systematic reviews. 2021;10(1):24.
5. DRKS00012504. Deutsches Register Klinischer Studien (German Clinical Trials Register). The CARE for CAYA program - Comprehensive Assessments and Related interventions to Enhance long-term outcome in Children, Adolescents and Young Adults (CAYAs). 2018; https://www.drks.de/drks_web/navigate.do?navigationId=trial.HTML&TRIAL_ID=DRKS00012504.
6. DRKS00017453. Improving mental health care for unaccompanied young refugees through a stepped care approach. 2019;who.int/trialsearch/Trial2.aspx?TrialID=DRKS00017453. 2019.
7. Gijzen, Mandy W. M., Creemers, Daan H. M., Rasing, Sanne P. A., Smit, Filip, and Engels, Rutger C. M. E.. Evaluation of a multimodal school-based depression and suicide prevention program among Dutch adolescents: design of a cluster-randomized controlled trial. BMC psychiatry 2018. 18 (1) 124-.
8. Hansson, Kristian, Johansson, Bjorn Axel, Andersson, Claes, Rastam, Maria, and Eberhard, Sophia. Issues in Child and Adolescent Inpatient Assessment and Evaluation After Discharge: Protocol for App Development and a Randomized Controlled Trial. JMIR research protocols 2018. 7 (11) e10121-.
9. ISRCTN34770541. A manualised preventive counselling program for children of parents with cancer. http://www.who.int/trialsearch/Trial2.aspx?TrialID=ISRCTN34770541 2009. () -.
10. Korczak DJ, Finkelstein Y, Barwick M, Chaim G, Cleverley K, Henderson J, et al. A suicide prevention strategy for youth presenting to the emergency department with suicide related behaviour: protocol for a randomized controlled trial. BMC psychiatry. 2020;20(1):20, 2020.
11. Loades, M. Universal school-based mental health programmes for the prevention of anxiety and depression amongst children and adolescent learners in low- and middle-income (LMIC) settings: a systematic review. Prospero 2019. () -.
12. NCT00280319. Study of Treatments for Depression Among Displaced Adolescents in Northern Uganda. 2006;gov/show/NCT00280319. 2006.
13. NCT00282776. Identification and Therapy of Postpartum Depression. https://clinicaltrials.gov/show/NCT00282776 2006. () -.
14. NCT00338806. Early Detection and Prevention of Mood Disorders in Children of Parents With Bipolar Disorder. https://clinicaltrials.gov/show/NCT00338806 2006. () -.
15. NCT00505440. Trial of Automated Risk Appraisal for Adolescents. 2007;gov/show/NCT00505440. 2007.
16. NCT00507299. A Safe Environment for Every Kid (SEEK) I. 2007;gov/show/NCT00507299. 2007.
17. NCT00891631. Primary Care iSBIRT to Reduce Serious Teen Health Risks. https://clinicaltrials.gov/show/NCT00891631 2009. () -.
18. NCT01220635. Middle School Matters Study. https://clinicaltrials.gov/show/NCT01220635 2010. () -.
19. NCT01228890. Primary Care Internet-Based Depression Prevention for Adolescents (CATCH-IT). https://clinicaltrials.gov/show/NCT01228890 2010. () -.
20. NCT01464619. Enhancing Parenting for Depressed Caregivers. https://clinicaltrials.gov/show/NCT01464619 2011. () -.
21. NCT02169960. Comprehensive Program for Youth Mental Health. 2014;gov/show/NCT02169960. 2014.
22. NCT02760004. PRogram In Support of Moms: an Innovative Stepped-Care Approach for Obstetrics and Gynecology Clinics. https://clinicaltrials.gov/show/NCT02760004 2016. () -.
23. NCT02798354. Reducing Diagnostic Errors in Primary Care Pediatrics (Project RedDE). https://clinicaltrials.gov/show/NCT02798354 2016. () -.
24. NCT02938598. Motivating Our Mothers 2. https://clinicaltrials.gov/show/NCT02938598 2016. () -.
25. NCT03388606. Characterization and Treatment of Adolescent Depression. https://clinicaltrials.gov/show/NCT03388606 2017. () -.
26. NCT03552900. Evaluating a Mobile App for Students Seeking Care for Depression and Anxiety at Harvard University Health Services. https://clinicaltrials.gov/show/NCT03552900 2018. () -.
27. NCT03779477. Effect of Coping With Stress Program to Depression, Anxiety, Brain Functions in Adolescent at High-Risk for Depression. https://clinicaltrials.gov/show/NCT03779477 2018. () -.
28. NCT03932760. Telehealth Group Intervention for Perinatal Depressive Symptoms. https://clinicaltrials.gov/show/NCT03932760 2019. () -.
29. NCT04069091. Implementation of Prevention and Intervention of Maternal Perinatal Depression to Strengthen Maternal and Child Health. https://clinicaltrials.gov/show/NCT04069091 2019. () -.
30. NCT04132856. Mapping Psychosocial Screening to Services for Children With Cancer. 2019;gov/show/NCT04132856. 2019.
31. NCT04489485. Online System for Identifying and Addressing Teen Depression in Primary Care. 2020;gov/show/NCT04489485. 2020.
32. NCT04522453. E-MhGAP Intervention Guide in Low- and Middle-income Countries: proof-of-concept for Impact and Acceptability. 2020;gov/show/NCT04522453. 2020.
33. NCT04522453. E-MhGAP Intervention Guide in Low- and Middle-income Countries: proof-of-concept for Impact and Acceptability. 2020;gov/show/NCT04522453. 2020.
34. NCT04587661. Designing an Implementation Strategy for Delivering Routine Mental Health Screening and Treatment. 2020;gov/show/NCT04587661. 2020.
35. NCT04598958. A Multidisciplinary “Integrated Management Team to Improve Maternal-Child Outcomes (IMPROVE)” Intervention to Improve Maternal and Child Outcomes in Lesotho. 2020;gov/show/NCT04598958. 2020.
36. NCT04646369 (2020) Screening Wizard- Phase 2. gov/show/NCT04646369. 2020.:
37. NL7719. Strong teens and resilient minds: depression and Suicide Prevention in Higher Vocational Education. http://www.who.int/trialsearch/Trial2.aspx?TrialID=NL7719 2019.
38. NTR4850. Online prevention of anxiety and depression in adolescents - Phase II. http://www.who.int/trialsearch/Trial2.aspx?TrialID=NTR4850 2014. () -.
39. Rosner R, Sachser C, Hornfeck F, Kilian R, Kindler H, Muche R, et al. Improving mental health care for unaccompanied young refugees through a stepped-care approach versus usual care+: study protocol of a cluster randomized controlled hybrid effectiveness implementation trial. Trials. 2020;21(1):1013, 2020.
40. Screening Wizard, Component 1 of iCHART (Integrated Care to Help At-Risk Teens)-Feasibility/Pilot Phase. www.clinicaltrials.gov 2019.
41. Sekhar, Deepa L., Pattison, Krista L., Confair, Alexandra, Molinari, Alissa, Schaefer, Eric W., Waxmonsky, James G., Walker-Harding, Leslie R., Rosen, Perri, and Kraschnewski, Jennifer L.. Effectiveness of Universal School-Based Screening vs Targeted Screening for Major Depressive Disorder Among Adolescents: A Trial Protocol for the Screening in High Schools to Identify, Evaluate, and Lower Depression (SHIELD) Randomized Clinical Trial. JAMA network open 2019. 2 (11) e1914427-.
42. Thabrew, Hiran, Corter, Arden, Goodyear-Smith, Felicity, and Goldfinch, Mary. Randomized Trial Comparing the Electronic Composite Psychosocial Screener YouthCHAT With a Clinician-Interview Assessment for Young People: A Study Protocol. JMIR research protocols 2017. 6 (7) e135-.

### Published before 2017 (n=81)

1. Ahlen, Johan, Lenhard, Fabian, and Ghaderi, Ata. Universal Prevention for Anxiety and Depressive Symptoms in Children: A Meta-analysis of Randomized and Cluster-Randomized Trials. Journal of Primary Prevention 2015. 36 (6) 387-403.
2. Allenby, A.. The application of computer touch-screen technology in screening for psychosocial distress in an ambulatory oncology setting. European journal of cancer care 2002. 11 (4) 245-.
3. Angold, A.. Psychiatric diagnostic interviews for children and adolescents: a comparative study. Journal of the American Academy of Child and Adolescent Psychiatry 2012. 51 (5) 506-.
4. Bauman LJ, Silver EJ Draimin BH Hudis. Children of mothers with HIV/AIDS: unmet needs for mental health services. Pediatrics 2007. 120 (5) e1141-.
5. Beck CT, Gable RK. Comparative analysis of the performance of the Postpartum Depression Screening Scale with two other depression instruments. Nursing research 2001. 50 (4) 242-.
6. Birmingham MC, Chou KJ Crain EF. Screening for postpartum depression in a pediatric emergency department. Pediatric emergency care 2011. 27 (9) 795-.
7. Blackmore ER, Carroll J.. The use of the Antenatal Psychosocial Health Assessment (ALPHA) tool in the detection of psychosocial risk factors for postpartum depression: a randomized controlled trial. Journal d'obstetrique et gynecologie du Canada : JOGC [Journal of obstetrics and gynaecology Canada : JOGC] 2006. 28 (10) 873-.
8. Bolton, P.. Interventions for depression symptoms among adolescent survivors of war and displacement in northern Uganda: a randomized controlled trial. JAMA 2007. 298 (5) 519-.
9. Bush JS, Ownby DR Waller JL Tingen MS. Risk factors for depression in rural adolescents with asthma. Journal of allergy and clinical immunology 2015. 135 (2) AB244-.
10. Calear AL, Christensen H.. Systematic review of school-based prevention and early intervention programs for depression. Journal of adolescence 2010. 33 (3) 429-.
11. Calear AL, Christensen H.. The Y-Worri Project: study protocol for a randomised controlled trial. Trials 2013. 14 () 76-.
12. Carroll AE, Biondich P.. A randomized controlled trial of screening for maternal depression with a clinical decision support system. Journal of the american medical informatics association : JAMIA 2013. 20 (2) 311-.
13. Carroll JC, Reid AJ Biringer. Effectiveness of the Antenatal Psychosocial Health Assessment (ALPHA) form in detecting psychosocial concerns: a randomized controlled trial. CMAJ : Canadian Medical Association journal 2005. 173 (3) 253-.
14. Connelly CD, Hazen AL Baker-Ericzen MJ Landsverk. Is screening for depression in the perinatal period enough? The co-occurrence of depression, substance abuse, and intimate partner violence in culturally diverse pregnant women. Journal of Women's Health 2013. 22 (10) 844-.
15. Crawford MJ, Thana L.. Impact of screening for risk of suicide: randomised controlled trial. British journal of psychiatry 2011. 198 (5) 379-.
16. Ebesutani, C.. The Revised Child Anxiety and Depression Scale-Short Version: scale reduction via exploratory bifactor modeling of the broad anxiety factor. Psychological assessment 2012. 24 (4) 833-.
17. Eisen JC, Marko Holguin. Pilot study of implementation of an internet-based depression prevention intervention (CATCH-IT) for adolescents in 12 US primary care practices: clinical and management/organizational behavioral perspectives. Primary care companion to the journal of clinical psychiatry 2013. 15 (6) -.
18. Esbensen AJ, Rojahn J.. Reliability and validity of an assessment instrument for anxiety, depression, and mood among individuals with mental retardation. Journal of autism and developmental disorders 2003. 33 (6) 617-.
19. Feigelman S, Dubowitz. Training pediatric residents in a primary care clinic to help address psychosocial problems and prevent child maltreatment. Academic pediatrics 2011. 11 (6) 474-.
20. Fraser JA, Armstrong KL Morris JP Dadds MR. Home visiting intervention for vulnerable families with newborns: follow-up results of a randomized controlled trial. Child abuse & neglect 2000. 24 (11) 1399-.
21. Gale S, Harlow BL. Postpartum mood disorders: a review of clinical and epidemiological factors. Journal of psychosomatic obstetrics and gynaecology 2003. 24 (4) 257-.
22. Garcia E, Joseph. Pediatric-based intervention to motivate mothers to seek follow-up for depression screens: the Motivating Our Mothers (MOM) trial. Academic pediatrics 2015. 15 (3 // 1K23MH101157-01A1 (NCATS) *National Center for Advancing Translational Sciences* // 1K23MH101157-01A1 (NIH) *National Center for Advancing Translational Sciences* // UL1 TR000002 (NCATS) *National Center for Advancing Translational Sciences* // UL1 TR000002 (NIH) *National Center for Advancing Translational Sciences*) 311-.
23. Gega, L.. Screening people with anxiety/depression for suitability for guided self-help. Cognitive behaviour therapy 2005. 34 (1) 16-.
24. Gladstone TG, Marko Holguin. An internet-based adolescent depression preventive intervention: study protocol for a randomized control trial. Trials 2015. 16 () 203-.
25. Goetz DM, Rand K.. Screening for depression in caregivers of childrens with cystic fibrosis. Pediatric Pulmonology 2016. 51 Suppl 45 () 456-.
26. Gonzalez GM, Costello CR La Tourette TR Joyce LK Valenzuela. Bilingual telephone-assisted computerized speech-recognition assessment: is a voice-activated computer program a culturally and linguistically appropriate tool for screening depression in English and Spanish?. Cultural diversity and mental health 1997. 3 (2) 93-.
27. Gould MS, Marrocco FA Kleinman. Evaluating iatrogenic risk of youth suicide screening programs: a randomized controlled trial. JAMA 2005. 293 (13) 1635-.
28. Hallfors, D.. Feasibility of screening adolescents for suicide risk in "real-world" high school settings. American journal of public health 2006. 96 (2) 282-.
29. Haugen, W.. Identifying depression among adolescents using three key questions: a validation study in primary care. British journal of general practice 2016. 66 (643) e65-.
30. Hegarty, K.. Screening and counselling in the primary care setting for women who have experienced intimate partner violence (WEAVE): a cluster randomised controlled trial. Lancet (London, England) 2013. 382 (9888) 249-.
31. Hendrick, V.. Treatment of postnatal depression. British medical journal 2003. 327 (7422) 1003-.
32. Hoek, W.. Randomized controlled trial of primary care physician motivational interviewing versus brief advice to engage adolescents with an Internet-based depression prevention intervention: 6-month outcomes and predictors of improvement. Translational research 2011. 158 (6) 315-.
33. Hoodin, F.. Impact of psychological screening on routine outpatient care of hematopoietic cell transplantation survivors. Biology of blood and marrow transplantation 2013. 19 (10) 1493-.
34. Horigian VE, Weems CF Robbins MS Feaster DJ Ucha. Reductions in anxiety and depression symptoms in youth receiving substance use treatment. The american journal on addictions 2013. 22 (4) 329-.
35. Iheanacho, Theddeus, Stefanovics, Elina, Rosenheck, Robert, Kapadia, Daniel, Ezeanolue, Chinenye O., Obiefune, Michael, Patel, Dina, Ezeanolue, Echezona E., Osuji, Alice A., Ogidi, Amaka G., and Ike, Anulika. Attitudes and beliefs about mental illness among church-based lay health workers: experience from a prevention of mother-to-child HIV transmission trial in Nigeria. International Journal of Culture & Mental Health 2016. 9 (1) 1-13.
36. Jardri, R.. Predictive validation study of the Edinburgh Postnatal Depression Scale in the first week after delivery and risk analysis for postnatal depression. Journal of affective disorders 2006. 93 (1-3) 169-.
37. Kahn, J.-P.. Important variables when screening for students at suicidal risk: findings from the French cohort of the SEYLE study. International journal of environmental research and public health 2015. 12 (10) 12277-.
38. Kastello JC, Jacobsen KH Gaffney KF Kodadek MP Bullock LC Sharps PW. Self-Rated Mental Health: screening for Depression and Posttraumatic Stress Disorder Among Women Exposed to Perinatal Intimate Partner Violence. Journal of psychosocial nursing and mental health services 2015. 53 (11) 32-.
39. Katon, W.. Anxiety and depression screening for youth in a primary care population. Ambulatory pediatrics 2008. 8 (3) 182-.
40. Kerker BD, Chor KHB Hoagwood KE Radigan. Detection and treatment of mental health issues by pediatric PCPs in New York state: an evaluation of project TEACH. Psychiatric services (washington, D.C.) 2015. 66 (4 // P30MH090322 (NIMH) *National Institute of Mental Health*) 430-.
41. King CA, Hill RM Wynne HA Cunningham RM. Adolescent suicide risk screening: the effect of communication about type of follow-up on adolescents' screening responses. Journal of Clinical Child and Adolescent Psychology 2012. 41 (4) 508-.
42. Kingston, D.. Study protocol for a randomized, controlled, superiority trial comparing the clinical and cost- effectiveness of integrated online mental health assessment-referral-care in pregnancy to usual prenatal care on prenatal and postnatal mental health and infant health and development: the Integrated Maternal Psychosocial Assessment to Care Trial (IMPACT). Trials 2014. 15 () 72-.
43. Larsen AR, Engsbro AL Bytzer. Screening instruments for anxiety and depression in patients with irritable bowel syndrome are ambiguous. Danish medical journal 2014. 61 (2) A4785-.
44. Lee, Eleanor W., Denison, Fiona C., Hor, Kahyee, and Reynolds, Rebecca M.. Web-based interventions for prevention and treatment of perinatal mood disorders: a systematic review. BMC Pregnancy & Childbirth 2-29-2016. 16 () 1-8.
45. Leng JC, Changrani J.. Detection of depression with different interpreting methods among Chinese and Latino primary care patients: a randomized controlled trial. Journal of immigrant and minority health 2010. 12 (2) 234-.
46. Leung SS, Leung C.. Outcome of a postnatal depression screening programme using the Edinburgh Postnatal Depression Scale: a randomized controlled trial. Journal of public health (oxford, england) 2011. 33 (2) 292-.
47. Lin, H., Zhou, S., Zhang, D., and Huang, L.. Evaluation of a nurse-led management program to complement the treatment of adolescent acute lymphoblastic leukemia patients. Applied nursing research : ANR 2016. 32 () e1-e5.
48. Lumley, J.. PRISM (Program of Resources, Information and Support for Mothers) Protocol for a community-randomised trial. BMC public health 2003. 3 () 36-.
49. Matthey, S.. Assessing for psychosocial morbidity in pregnant women. Canadian medical association journal 2005. 173 (3) 267-.
50. Miller FG, Wendler D.. When do the federal regulations allow placebo-controlled trials in children?. Journal of pediatrics 2003. 142 (2) 102-.
51. Moor S, Maguire. Improving the recognition of depression in adolescence: can we teach the teachers?. Journal of adolescence 2007. 30 (1) 81-.
52. Nauta MH, Festen H.. Preventing mood and anxiety disorders in youth: a multi-centre RCT in the high risk offspring of depressed and anxious patients. BMC psychiatry 2012. 12 (1) -.
53. Niemi, M.. Community-based intervention for depression management at the primary care level in Ha Nam Province, Vietnam: a cluster-randomised controlled trial. Tropical medicine & international health 2016. 21 (5) 654-.
54. Olson AL, Dietrich AJ Prazar. Two approaches to maternal depression screening during well child visits. Journal of developmental and behavioral pediatrics 2005. 26 (3) 169-.
55. Paradis HA, Sandler M.. Building healthy children: evidence-based home visitation integrated with pediatric medical homes. Pediatrics 2013. 132 (SUPPL.2) S174-.
56. Peindl KS, Wisner KL Hanusa BH. Identifying depression in the first postpartum year: guidelines for office-based screening and referral. Journal of affective disorders 2004. 80 (1) 37-.
57. Puertas, G.. Are visual measures of mood superior to questionnaire measures in non-Western settings?. Social psychiatry and psychiatric epidemiology 2004. 39 (8) 662-.
58. Rasmussen, A.. Development and validation of a Haitian Creole screening instrument for depression. Transcultural psychiatry 2015. 52 (1) 33-.
59. Richards, Derek, Richardson, Thomas, Timulak, Ladislav, Vigano, Noemi, Mooney, Jacqueline, Doherty, Gavin, Hayes, Claire, and Sharry, John. Predictors of depression severity in a treatment-seeking sample. International Journal of Clinical and Health Psychology 2016. 16 (3) 221-229.
60. Richards, Katie, Marko-Holguin, Monika, Fogel, Joshua, Anker, Lauren, Ronayne, James, and Van Voorhees, Benjamin W.. RANDOMIZED CLINICAL TRIAL OF AN INTERNET-BASED INTERVENTION TO PREVENT ADOLESCENT DEPRESSION IN A PRIMARY CARE SETTING (CATCH-IT): 2.5-YEAR OUTCOMES. Journal of evidence-based psychotherapies 2016. 16 (2) 113-134.
61. Roy-Byrne PP, Katon W.. Panic disorder in primary care: biopsychosocial differences between recognized and unrecognized patients. General hospital psychiatry 2000. 22 (6) 405-.
62. Rush AJ, Trivedi MH Carmody TJ Ibrahim HM Markowitz JC Keitner GI Kornstein SG Arnow. Self-reported depressive symptom measures: sensitivity to detecting change in a randomized, controlled trial of chronically depressed, nonpsychotic outpatients. Neuropsychopharmacology 2005. 30 (2) 405-.
63. Salgado PCB, Nogueira MH Yasuda CL Cendes. Screening symptoms of depression and suicidal ideation in people with epilepsy using the Beck depression inventory. Journal of epilepsy and clinical neurophysiology 2012. 18 (3) 85-.
64. Salokangas RK, Poutanen O.. Detection, diagnosis and treatment of depression by primary care physicians. Duodecim; laaketieteellinen aikakauskirja 1996. 112 (4) 265-. [Finnish]
65. Samuel VM, Colville GA Goodwin S Ryninks. The value of screening parents for their risk of developing psychological symptoms after PICU: a feasibility study evaluating a pediatric intensive care follow-up clinic. Pediatric critical care medicine 2015. 16 (9) 808-.
66. Schrader, G.. Effect of psychiatry liaison with general practitioners on depression severity in recently hospitalised cardiac patients: a randomised controlled trial. Medical journal of Australia 2005. 182 (6) 272-.
67. Schubiner, H.. The clinical utility of the Safe Times Questionnaire. Journal of Adolescent Health 1994. 15 (5) 374-.
68. Seekles, W.. Stepped care for depression and anxiety: from primary care to specialized mental health care: a randomised controlled trial testing the effectiveness of a stepped care program among primary care patients with mood or anxiety disorders. BMC health services research 2009. 9 () 90-.
69. Stallard, P.. Protocol for a randomised controlled trial of a school based cognitive behaviour therapy (CBT) intervention to prevent depression in high risk adolescents (PROMISE). Trials 2010. 11 () 114-.
70. Stamp GE, Williams AS Crowther CA. Predicting postnatal depression among pregnant women. Birth (Berkeley, Calif.) 1996. 23 (4) 218-.
71. Stein, G.. The retrospective diagnosis of postnatal depression by questionnaire. J-psychosom-res 1992. 36 (1) 67-.
72. Stevens, J.. Trial of computerized screening for adolescent behavioral concerns. Pediatrics 2008. 121 (6) 1099-.
73. Tait RJ, French DJ Hulse GK. Validity and psychometric properties of the General Health Questionnaire-12 in young Australian adolescents. Australian and New Zealand Journal of Psychiatry 2003. 37 (3) 374-.
74. Teissedre, F.. A study of the Edinburgh Postnatal Depression Scale (EPDS) on 859 mothers: detection of mothers at risk for postpartum depression. Encephale 2004. 30 (4) 376-.
75. Valtonen, M.. Enhancing sense of coherence via early intervention among depressed occupational health care clients. Nordic journal of psychiatry 2015. 69 (7) 515-
76. Venkatesh KK, Zlotnick C.. Accuracy of brief screening tools for identifying postpartum depression among adolescent mothers. Pediatrics 2014. 133 (1) e45-.
77. Weobong, B.. The comparative validity of screening scales for postnatal common mental disorder in Kintampo, Ghana. Journal of affective disorders 2009. 113 (1-2) 109-.
78. Wickberg, B.. Counselling of postnatal depression: a controlled study on a population based Swedish sample. Journal of affective disorders 1996. 39 (3) 209-.
79. Wright DR, Haaland WL Ludman E McCauley. The Costs and Cost-effectiveness of Collaborative Care for Adolescents With Depression in Primary Care Settings: a Randomized Clinical Trial. JAMA pediatrics 2016. 170 (11) 1048-.
80. Zhang DX, Lewis G.. Prevention of anxiety and depression in Chinese: a randomized clinical trial testing the effectiveness of a stepped care program in primary care. Journal of affective disorders 2014. 169 () 212-.
81. Zimmermann, T.. Effectiveness of a primary care based complex intervention to promote self-management in patients presenting psychiatric symptoms: study protocol of a cluster-randomized controlled trial. BMC psychiatry 2014. 14 () 2-.

### Study design (n=26)

1. Abo Elasrar M, Hany Elrassas H, Adel Thabet R, Seifeldin Abdeen M, Eldeen Nouby Mohamed Elazab A. Obesity and diabetic control as predictors for depression in adolescents with type 1 diabetes mellitus. Vulnerable Children and Youth Studies2020, ppNo Pagination Specified. 2020;
2. Abstracts from Research Forums Presented at the American College of Nurse-Midwives' 62nd Annual Meeting. Journal of midwifery & women's health 2017. 62 (5) -.
3. Barrera, M., Solomon, A., Chung, J., Alexander, S., Mills, D., Shama, W., and Hancock, K.. Psychosocial screening implementation and mental health outcomes in the patients, caregivers and siblings. Pediatric Blood and Cancer 2018. 65 (Supplement 2) S563-.
4. Byatt N, Brenckle L, Sankaran P, Biebel K, Weinreb L, Allison J, et al. Improving perinatal depression care in obstetric settings: PRogram in Support of Moms (PRISM). Archives of women’s mental health. 2019;Conference:(5):702.
5. Costantine MM, Miller ES. 224 The association between perinatal depressive symptoms and child neurodevelopment. American Journal of Obstetrics and Gynecology. 2021;Conference:(2 Supplement):S148–9.
6. Desjardins, L., Hancock, K., Alexander, S., Mills, D., Shama, W., De, Souza C., Gupta, A., and Szatmari, P.. Mapping psychosocial screening to resources: A pilot intervention study. Pediatric Blood and Cancer 2018. 65 (Supplement 2) S8-.
7. Diez-Gomez A, Perez-Albeniz A, Ortuno-Sierra J, Fonseca-Pedrero E. SENTIA: An Adolescent Suicidal Behavior Assessment Scale. Psicothema. 2020;32(3):382–9.
8. Harder, Valerie S., Barry, Sara E., French, Sarah, Consigli, Alyssa B., and Frankowski, Barbara L.. Improving Adolescent Depression Screening in Pediatric Primary Care. Academic pediatrics 2019. 19 (8) 925-933.
9. Jarbin H, Ivarsson T, Andersson M, Bergman H, Skarphedinsson G. Screening efficiency of the Mood and Feelings Questionnaire (MFQ) and Short Mood and Feelings Questionnaire (SMFQ) in Swedish help seeking outpatients. PloS one. 2020;15(3):e0230623, 2020.
10. Jones LS, Anderson E, Loades M, Barnes R, Crawley E. Can linguistic analysis be used to identify whether adolescents with a chronic illness are depressed? 2020;27(2):179–92.
11. Khesht-Masjedi, M. F. and Omar, Z.. Development of anxiety and depression inventory for secondary school students in Iran (A & D inventory). Iranian Journal of Psychiatry and Behavioral Sciences 2017. 11 (1) e3698-.
12. Lesage, A., Amor, L. B., Conrod, P., Geoffroy, M.-C., Kackzorowski, J., Moga, C., Mombo, N., Renaud, J., Vasiliadis, H.-M., Mongodin, A., and Gheorghiu, I.. A review of best practices in fivemental disorders in youth. International Journal of Technology Assessment in Health Care 2017. 33 (Supplement 1) 237-.
13. Lois, R.. Integrated behavioral health programs in subspecialty pediatrics. Journal of the American Academy of Child and Adolescent Psychiatry 2017. 56 (10) S17-.
14. Materu J, Kuringe E, Nyato D, Galishi A, Mwanamsangu A, Katebalila M, et al. The psychometric properties of PHQ-4 anxiety and depression screening scale among out of school adolescent girls and young women in Tanzania: a cross-sectional study. BMC psychiatry. 2020;20(1):321, 2020.
15. Sterling, S. A., Kline-Simon, A., Jones, A., Brumder-Ross, T., and Weisner, C.. Outcomes from a trial of screening, brief intervention and referral to treatment for adolescents in pediatric primary care: Implications for adolescent girls. Alcoholism: Clinical and Experimental Research 2017. 41 (Supplement 1) 336A-.
16. Wijesekera, K.. Innovative behavioral health screening and resilience building intervention for pediatric heart transplant youth and families: the focus-pedsht program. Pediatric transplantation 2019. 23 () -.
17. Bekman, S., John-Larkin, C., Paul, J. J., Millar, A., and Frankel, K.. Update on Screening, Referring, and Treating the Behavioral, Social, and Mental Health Problems of Very Young Children. Current Treatment Options in Pediatrics 2017. 3 (1) 15-31.
18. Brent DA. 2.5 Novel approaches to the assessment and treatment of suicidal adolescents. Journal of the American Academy of Child and Adolescent Psychiatry. 2020;Conference:(10 Supplement):S125–6.
19. Cheung, A. Guidelines for Adolescent Depression in Primary Care (GLAD-PC) Toolkit. The REACH Institute 2019. () -.
20. Correction (Journal of the American Academy of Child & Adolescent Psychiatry (2020) 59(6) (770-773), (S0890856720302197), (10.1016/j.jaac.2020.01.025)). Journal of the American Academy of Child and Adolescent Psychiatry. 2020;59(12):1408–10.
21. Depression in children and young people: Young people: identification and management. National Institute for Health and Care Excellence 2019. () -.
22. Goodyear-Smith, Felicity, Martel, Rhiannon, Darragh, Margot, Warren, Jim, Thabrew, Hiran, and Clark, Terryann C.. Screening for risky behaviour and mental health in young people: the YouthCHAT programme. Public health reviews 2017. 38 () 20-.
23. Hankin BL. Screening for and personalizing prevention of adolescent depression. Current Directions in Psychological Science. 2020;29(4):327–32.
24. RA Zuckerbrot, A Cheung, PS Jensen, REK Stein, D Laraque, . Guidelines for Adolescent Depression in Primary Care (GLAD-PC): Part I. Practice Preparation, Identification, Assessment, and Initial Management.. 2018. 141 (3) -.
25. Spielvogle, H., McCarty, C. A., and Richardson, L. P.. Brief therapy for anxiety and depression in the pediatric primary care setting: Implications and next steps. JAMA pediatrics 2017. 171 (10) 1006-1007.
26. Thabrew H, D’Silva S, Darragh M, Goldfinch M, Meads J, Goodyear-Smith F. “Comparison of YouthCHAT, an electronic composite psychosocial screener, with a clinician interview assessment for young people: Randomized trial.”: Correction. Journal of medical Internet research. 2020;22(2).

### Systematic, narrative, or literature review (n=10)

1. Conejo-Ceron, Sonia, Moreno-Peral, Patricia, Rodriguez-Morejon, Alberto, Motrico, Emma, Navas-Campana, Desiree, Rigabert, Alina, Martin-Perez, Carlos, Rodriguez-Bayon, Antonina, Ballesta-Rodriguez, Maria Isabel, de Dios Luna, Juan, Garcia-Campayo, Javier, Roca, Miquel, and Bellon, Juan Angel. Effectiveness of psychological and educational interventions to prevent depression in primary care: A systematic review and meta-analysis. Annals of family medicine 2017. 15 (3) 262-271.
2. Forte A, Sarli G, Polidori L, Lester D, Pompili M. The Role of New Technologies to Prevent Suicide in Adolescence: A Systematic Review of the Literature. Medicina (Kaunas). 2021;57(2).
3. Kirkland, S. W., Soleimani, A., and Newton, A. S.. Review: The impact of pediatric mental health care provided outpatient, primary care, community and school settings on emergency department use - a systematic review. Child and Adolescent Mental Health 2018. 23 (1) 4-13.
4. Littell JH, Winsvold A.. Functional Family Therapy for families of youth (age 11-18) with behaviour problems. Cochrane database of systematic reviews (online) 2007. (2) -.
5. McVoy M, Fulchiero E, Hardin HK, Neudecker M, Sajatovic M. 9.6 Mental health comorbidity and youth-onset type 2 diabetes: A systematic review of the literature. Journal of the American Academy of Child and Adolescent Psychiatry. 2020;Conference:(10 Supplement):S174.
6. Melbye S, Kessing LV, Bardram JE, Faurholt-Jepsen M. Smartphone-Based Self-Monitoring, Treatment, and Automatically Generated Data in Children, Adolescents, and Young Adults With Psychiatric Disorders: Systematic Review. JMIR mental health. 2020;7(10):e17453, 2020.
7. Nelson HD, Cantor A, Pappas M, Weeks C. Screening for Anxiety in Adolescent and Adult Women: A Systematic Review for the Women’s Preventive Services Initiative. Ann Intern Med. 2020;173(1):29–41.
8. Roseman, Michelle, Saadat, Nazanin, Riehm, Kira E., Kloda, Lorie A., Boruff, Jill, Ickowicz, Abel, Baltzer, Franziska, Katz, Laurence Y., Patten, Scott B., Rousseau, Cecile, and Thombs, Brett D.. Depression Screening and Health Outcomes in Children and Adolescents: A Systematic Review. Canadian journal of psychiatry.Revue canadienne de psychiatrie 2017. 62 (12) 813-817.
9. Thombs, Brett D., Saadat, Nazanin, Riehm, Kira E., Karter, Justin Michael, Vaswani, Akansha, Andrews, Bonnie K., Simons, Peter, and Cosgrove, Lisa. Consistency and sources of divergence in recommendations on screening with questionnaires for presently experienced health problems or symptoms: a comparison of recommendations from the Canadian Task Force on Preventive Health Care, UK National Screening Committee, and US Preventive Services Task Force. BMC medicine 2017. 15 (1) 150-.
10. Werner-Seidler, Aliza, Perry, Yael, Calear, Alison L., Newby, Jill M., and Christensen, Helen. School-based depression and anxiety prevention programs for young people: A systematic review and meta-analysis. Clinical psychology review 2017. 51 () 30-47.
