# Supplementary material for: Screening for depression in children and adolescents in primary care or non-mental health settings: a systematic review update
Source: Syst Rev. 2024 Jan 31;13:48. doi: 10.1186/s13643-023-02447-3 (PMC10829174; doi:10.1186/s13643-023-02447-3)
Supplement: Supplementary file 7 — Additional file 7. List of excluded studies with reasons (non-randomized controlled studies). [file 13643_2023_2447_MOESM7_ESM.docx]

## Additional file 7: List of excluded studies with reasons (non-randomized controlled studies project)

### Adult population (n=11)

1. Akena D, Joska J, Stein DJ. Sensitivity and specificity of the Akena Visual Depression Inventory (AViDI-18) in Kampala (Uganda) and Cape Town (South Africa). British Journal of Psychiatry. 2018;212(5):301–7.
2. Alsaadi T, El Hammasi K, Shahrour TM, Shakra M, Turkawi L, Nasreddine W, et al. Depression and Anxiety among Patients with Epilepsy and Multiple Sclerosis: UAE Comparative Study. Behavioural Neurology. 2015;2015:196373.
3. Chung K, Jeon M-J, Park J, Lee S, Kim CO, Park JY, et al. Development and evaluation of a mobile-optimized daily self-rating depression screening app: A preliminary study. PLOS ONE. 2018;13(6):e0199118.
4. Cohen ML, Holdnack JA, Kisala PA, Tulsky DS. A comparison of PHQ-9 and TBI-QOL depression measures among individuals with traumatic brain injury. Rehabilitation Psychology. 2018;63(3):365–71.
5. Gonzalez-Blanch C, Medrano LA, Munoz-Navarro R, Ruiz-Rodriguez P, Moriana JA, Limonero JT, et al. Factor structure and measurement invariance across various demographic groups and over time for the PHQ-9 in primary care patients in Spain. PLoS ONE [Electronic Resource]. 2018;13(2):e0193356.
6. Gunn J, Wachtler C, Fletcher S, Davidson S, Mihalopoulos C, Palmer V, et al. Target-D: a stratified individually randomized controlled trial of the diamond clinical prediction tool to triage and target treatment for depressive symptoms in general practice: study protocol for a randomized controlled trial. Trials [Electronic Resource]. 2017;18(1):342.
7. Hanna J, Santo JB, Blair M, Smolewska K, Warriner E, Morrow SA. Comparing depression screening tools in persons with multiple sclerosis (MS). Rehabilitation Psychology. 2017;62(1):20–4.
8. Husain N, Chaudhry N, Rhouma A, Sumra A, Tomenson B, Waheed W. Validation of the self-reporting questionnaire (SRQ 20) in British Pakistani and White European population in the United Kingdom. Journal of Affective Disorders. 2016;189:392–6.
9. Jin H, Wu S. Text Messaging as a Screening Tool for Depression and Related Conditions in Underserved, Predominantly Minority Safety Net Primary Care Patients: Validity Study. Journal of Medical Internet Research. 2020;22(3):e17282.
10. Mallen CD, Nicholl BI, Lewis M, Bartlam B, Green D, Jowett S, et al. The effects of implementing a point-of-care electronic template to prompt routine anxiety and depression screening in patients consulting for osteoarthritis (the Primary Care Osteoarthritis Trial): A cluster randomised trial in primary care. PLOS Medicine. 2017;14(4):e1002273.
11. Recklitis, C. J., Blackmon, J. E., & Chang, G. (2020). Screening young adult cancer survivors with the PROMIS Depression Short Form (PROMIS-D-SF): Comparison with a structured clinical diagnostic interview. *Cancer (0008543X)*, *126*(7), 1568–1575. https://doi.org/10.1002/cncr.32689

### Both groups received screening (n=2)

1. Carrozzino D, Marchetti D, Laino D, Minna M, Verrocchio MC, Fulcheri M, et al. Anxiety in adolescent epilepsy. A clinimetric analysis. Nordic Journal of Psychiatry. 2016;70(6):424–9.
2. Harder VS, Barry SE, French S, Consigli AB, Frankowski BL. Improving Adolescent Depression Screening in Pediatric Primary Care. Academic Pediatrics. 2019;19(8):925–33.

### Not relevant to screening (n=22)

1. Barrera M, Solomon A, Chung J, Alexander S, Mills D, Shama W, et al. Psychosocial screening implementation and mental health outcomes in the patients, caregivers and siblings. Pediatric Blood and Cancer. 2018;65 (Supplement 2):S563.
2. Cordeiro ML, Farias AC, Whybrow PC, Felden EPG, Cunha A, da Veiga V Jr, et al. Receiver Operating Characteristic Curve Analysis of Screening Tools for Bipolar Disorder Comorbid With ADHD in Schoolchildren. Journal of Attention Disorders. 2020;24(10):1403–12.
3. Coutinho D, Farias AC, Felden EPG, Cordeiro ML. ADHD Comorbid With Major Depression on Parents and Teachers Perceptions. Journal of Attention Disorders. 2018;1087054718815574.
4. Dembo R, Faber J, Cristiano J, DiClemente RJ, Krupa JM, Wareham J, et al. Psychometric Evaluation of a Brief Depression Measure for Justice-Involved Youths: A Multigroup Comparison. Journal of Child & Adolescent Substance Abuse. 2018;27(3):146–55.
5. Desjardins L, Hancock K, Alexander S, Mills D, Shama W, De Souza C, et al. Mapping psychosocial screening to resources: A pilot intervention study. Pediatric Blood and Cancer. 2018;65 (Supplement 2):S8.
6. Dölitzsch C, Kölch M, Fegert JM, Schmeck K, Schmid M. Ability of the Child Behavior Checklist-Dysregulation Profile and the Youth Self Report-Dysregulation Profile to identify serious psychopathology and association with correlated problems in high-risk children and adolescents. Journal of Affective Disorders. 2016;205:327–34.
7. Duru NS, Civilibal M, Elevli M. Quality of Life and Psychological Screening in Children with Type 1 Diabetes and their Mothers. Experimental and Clinical Endocrinology and Diabetes. 2016;124(2):105–10.
8. Gundogdu U, Fis NP, Eralp EE, Karadag BT. Major depression and psychiatric comorbidity in Turkish children and adolescents with cystic fibrosis. Pediatric Pulmonology. 2019;54(12):1927–35.
9. Heathcote LC, Rabner J, Lebel A, Hernandez JM, Simons LE. Rapid Screening of Risk in Pediatric Headache: Application of the Pediatric Pain Screening Tool. Journal of Pediatric Psychology. 2018;43(3):243–51.
10. Hood, A. M., Reife, I., King, A. A., & White, D. A. (2020). Brief Screening Measures Identify Risk for Psychological Difficulties Among Children with Sickle Cell Disease. *Journal of Clinical Psychology in Medical Settings*, *27*(4), 651–661. https://doi.org/10.1007/s10880-019-09654-y
11. Hurrell RA, Fullwood C, Keys J, Dickson AP, Fishwick J, Whitnall B, et al. Psychosocial screening at paediatric BEEC clinics: a pilot evaluation study. Journal of Pediatric Urology. 2015;11(2):79.e71-76.
12. Jones LS, Anderson E, Loades M, Barnes R, Crawley E. Can linguistic analysis be used to identify whether adolescents with a chronic illness are depressed? Clinical Psychology & Psychotherapy. 2020;27(2):179–92
13. Kahn JP. Suicide prevention and mental health promotion in adolescents: Lessons learned from the SEYLE “Saving and Empowering Young Lives in Europe” program. European Psychiatry. 2014;29(8):573.
14. Madjidova, Y., Sadikova, G., Ergasheva, N., Khusenova, N., & Abdumalyanova, N. (2020). Comparative evaluation of the effectiveness of screening techniques M-CHAT-R and cars. *International Journal of Pharmaceutical Research*, *12*(3), 1742–1748.
15. Malakouti SK, Nojomi M, Poshtmashadi M, Hakim Shooshtari M, Mansouri Moghadam F, Rahimi-Movaghar A, et al. Integrating a suicide prevention program into the primary health care network: a field trial study in Iran. BioMed Research International. 2015;2015:193729.
16. Parthasarathy, S., Kline-Simon, A. H., Jones, A., Hartman, L., Saba, K., Weisner, C., & Sterling, S. (2021). Three-Year Outcomes After Brief Treatment of Substance Use and Mood Symptoms. *Pediatrics*, *147*(1), 1–10. https://doi.org/10.1542/peds.2020-009191
17. Ratcliff MB, Catlin PA, Peugh JL, Siegel RM, Kirk S, Tamm L. Psychosocial Screening Among Youth Seeking Weight Management Treatment. Clinical Pediatrics. 2018;57(3):277–84.
18. Troister T, D’Agata MT, Holden RR. Suicide risk screening: Comparing the Beck Depression Inventory-II, Beck Hopelessness Scale, and Psychache Scale in undergraduates. Psychological Assessment. 2015;27(4):1500–6.
19. Van Den Brink G, Stapersma L, Vlug L, Rizopolous D, Bodelier A, Van Wering H, et al. Prevalence and risk factors for anxiety and depressive symptoms in children, adolescents and young adults with inflammatory bowel disease. Journal of Pediatric Gastroenterology and Nutrition. 2018;66 (Supplement 2):603.
20. van der Geest IMM, Pluijm SMF, van den Heuvel‐Eibrink MM, van Dorp W, van den Heuvel-Eibrink MM. The distress thermometer provides a simple screening tool for selecting distressed childhood cancer survivors. Acta Paediatrica. 2018;107(5):871–4.
21. Vieira MA, Gadelha AA, Moriyama TS, Bressan RA, Bordin IA. Evaluating the effectiveness of a training program that builds teachers’ capability to identify and appropriately refer middle and high school students with mental health problems in Brazil: an exploratory study. BMC Public Health. 2014;14:210.
22. Wiener L, Battles H, Zadeh S, Widemann BC, Pao M. Validity, specificity, feasibility and acceptability of a brief pediatric distress thermometer in outpatient clinics. Psycho Oncology [Internet]. 2015; Available from: http://onlinelibrary.wiley.com/journal/10.1002/(ISSN)1099-1611

### Protocol, study registration (n=11)

1. Beck A. Screening for depression in children and adolescents: a protocol for a systematic review update. PROSPERO. 2020;(CRD42020150373).
2. clinicaltrials.gov. Online System for Identifying and Addressing Teen Depression in Primary Care (Depression).
3. clinicaltrials.gov. Screening of Adolescent Mental Suffering (DESPERADOS).
4. clinicaltrials.gov. Universal vs. Targeted School Screening for Adolescent Major Depressive Disorder.
5. Gijzen MWM, Creemers DHM, Rasing SPA, Smit F, Engels R. Evaluation of a multimodal school-based depression and suicide prevention program among Dutch adolescents: design of a cluster-randomized controlled trial. BMC Psychiatry. 2018;18(1):124.
6. Hiran Thabrew. Psychosocial screening in children and adolescents with chronic physical illness. PROSPERO. 2015;(CRD42015016021).
7. Rashed AlRasheed. Barriers and facilitators to adolescent depression screening in primary care: a systematic review. PROSPERO. 2019;(CRD42019135278).
8. Rosner, R., Sachser, C., Hornfeck, F., Kilian, R., Kindler, H., Muche, R., Muller, L. R. F., Thielemann, J., Waldmann, T., Ziegenhain, U., Unterhitzenberger, J., & Pfeiffer, E. (2020). Improving mental health care for unaccompanied young refugees through a stepped-care approach versus usual care+: Study protocol of a cluster randomized controlled hybrid effectiveness implementation trial. *Trials*, *21*(1), 1013. https://doi.org/10.1186/s13063-020-04922-x
9. Sekhar DL, Pattison KL, Confair A, Molinari A, Schaefer EW, Waxmonsky JG, et al. Effectiveness of Universal School-Based Screening vs Targeted Screening for Major Depressive Disorder Among Adolescents: A Trial Protocol for the Screening in High Schools to Identify, Evaluate, and Lower Depression (SHIELD) Randomized Clinical Trial. JAMA Network Open. 2019;2(11):e1914427.
10. Thabrew H, Corter A, Goodyear-Smith F, Goldfinch M. Randomized Trial Comparing the Electronic Composite Psychosocial Screener YouthCHAT With a Clinician-Interview Assessment for Young People: A Study Protocol. JMIR Research Protocols. 2017;6(7):e135.
11. Wikberg, C., Augustsson, P., Sveinsdottir, G., Craighead, W. E., Arnarson, E. O., Marteinsdottir, I., & Lilja, J. L. (2021). Is the Thoughts and Health programme feasible in the context of Swedish schools? A quasi-experimental controlled trial study protocol. *BMJ Open*, *11*(1), e040374. https://doi.org/10.1136/bmjopen-2020-040374

### Study design (n=56)

1. Barrera M, Alexander S, Atenafu EG, Chung J, Hancock K, Solomon A, et al. Psychosocial screening and mental health in pediatric cancer: A randomized controlled trial. Health Psychology. 2020;39(5):381–90.
2. Barrera, M., Desjardins, L., Hancock, K., Prasad, S., Alexander, S., Shama, W., & Szatmari, P. (2020). Effectiveness of mapping psychosocial screening to resources: A one-year pilot randomized control intervention study. *Pediatric Blood and Cancer. Conference: 52th Congress of the International Society of Paediatric Oncology, SIOP*, *67*(SUPPL 4). https://doi.org/10.1002/pbc.28742
3. Bilginer C, Kandil S. Emotional and behavioral characteristics of childhood depression. Klinik Psikofarmakoloji Bulteni. 2015;1):S11–2.
4. Binagwaho A, Smith Fawzi MC, Agbonyitor M, Nsanzimana S, Karema C, Remera E, et al. Validating the Children’s Depression Inventory in the context of Rwanda. BMC Pediatrics. 2016;16:1–7.
5. Bruyere J, Burns JJ. A comparison between the frequency of problems on a standardized psychosocial screening tool in depressed vs. non-depressed adolescents. Journal of Investigative Medicine. 2020;68 (2):628–9.
6. Chernoff M, Angelidou KN, Williams PL, Brouwers P, Warshaw M, Nachman S, et al. Assessing Psychiatric Symptoms in Youth Affected by HIV: Comparing a Brief Self-Administered Rating Scale with a Structured Diagnostic Interview. Journal of Clinical Psychology in Medical Settings. 2018;25(4):420–8.
7. Dardas LA, Shoqirat N, Xu H, Al-Khayat A, Bani Ata S, Shawashreh A, et al. Comparison of the performance of the Beck Depression Inventory-II and the Center for Epidemiologic Studies-Depression Scale in Arab adolescents. Public Health Nursing. 2019;36(4):564–74.
8. de Jonge-Heesen KWJ, Rasing SPA, Vermulst AA, Scholte RHJ, van Ettekoven KM, Engels R, et al. Randomized control trial testing the effectiveness of implemented depression prevention in high-risk adolescents. BMC Medicine. 2020;18(1):188.
9. Duffus SH, Cooper KL, Agans RP, Jain N. Mental Health and Behavioral Screening in Pediatric Type 1 Diabetes. Diabetes Spectrum. 2019;32(2):171–5.
10. Farley AM, Gallop RJ, Brooks ES, Gerdes M, Bush ML, Young JF. Identification and Management of Adolescent Depression in a Large Pediatric Care Network. Behavioral Pediatrics. 2020;41(2):10.
11. Felder JN, Epel E, Lewis JB, Cunningham SD, Tobin JN, Rising SS, et al. Depressive symptoms and gestational length among pregnant adolescents: Cluster randomized control trial of CenteringPregnancy R plus group prenatal care. Journal of Consulting and Clinical Psychology. 2017;85(6):574–84.
12. Guo S, Kim JJ, Bear L, Lau AS. Does Depression Screening in Schools Reduce Adolescent Racial/Ethnic Disparities in Accessing Treatment? Journal of Clinical Child & Adolescent Psychology. 2017;46(4):523–36.
13. Haugen W, Christensen KS, Haavet OR, Sirpal MK. Identifying depression among adolescents using three key questions: A validation study in primary care. British Journal of General Practice. 2016;66(643):e65–70.
14. Hughes T, Rolling K, Locker A, Cardno A, West R, Marino-Francis F, et al. Unrecognised bipolar disorder among UK primary care patients prescribed antidepressants: an observational study. British Journal of General Practice. 2016;66(643):e71–7.
15. Jang SH, Woo YS, Hong JW, Yoon BH, Hwang TY, Kim MD, et al. Use of a smartphone application to screen for depression and suicide in South Korea. General Hospital Psychiatry. 2017;46:62–7.
16. Jha MK, Grannemann BD, Trombello JM, Clark EW, Eidelman SL, Lawson T, et al. A Structured Approach to Detecting and Treating Depression in Primary Care: VitalSign6 Project. Annals of Family Medicine. 2019;17(4):326–35.
17. Kabra AT, Feustel PJ, Kogan BA. Screening for depression and anxiety in childhood neurogenic bladder dysfunction. Journal of Pediatric Urology. 2015;11(2):75.e71-77.
18. Kahn JP, Tubiana A, Cohen RF, Carli V, Wasserman C, Hoven C, et al. Important Variables When Screening for Students at Suicidal Risk: Findings from the French Cohort of the SEYLE Study. International Journal of Environmental Research & Public Health [Electronic Resource]. 2015;12(10):12277–90.
19. Kellermann TS, Mueller M, Carter EG, Brooks B, Smith G, Kopp OJ, et al. Prediction of specific depressive symptom clusters in youth with epilepsy: The NDDI-E-Y versus Neuro-QOL SF. Epilepsia. 2017;58(8):1370–9.
20. Kelly D, Swan D, Cullen W. The effectiveness of educational interventions to improve the detection and/or treatment of adolescent depression in primary care. Irish Journal of Medical Science. 2017;186 (1 Supplement 1):S20.
21. Kernel Networks Inc. Screening Wizard, Component 1 of iCHART (Integrated Care to Help At-Risk Teens)-Feasibility/Pilot Phase. Case Medical Research. 2019;
22. Khesht-Masjedi MF, Omar Z. Development of anxiety and depression inventory for secondary school students in Iran (A & D inventory). Iranian Journal of Psychiatry and Behavioral Sciences [Internet]. 2017;11(1). Available from: http://cdn.neoscriber.org/cdn/dl/a129d0b8-321d-11e7-b8ec-f3582f24d585
23. Lee WK, Lim D, Lee HA, Park H. Sensation seeking as a potential screening tool for suicidality in adolescence. BMC Public Health. 2016;16(1):92–92.
24. Liu FF, Adrian MC. Is Treatment Working? Detecting Real Change in the Treatment of Child and Adolescent Depression. Journal of the American Academy of Child and Adolescent Psychiatry. 2019;58(12):1157–64.
25. Macalli, M., Côté, S., & Tzourio, C. (2020). Perceived parental support in childhood and adolescence as a tool for mental health screening in students: A longitudinal study in the i-Share cohort. *Journal of Affective Disorders*, *266*, 512–519. https://doi.org/10.1016/j.jad.2020.02.009
26. Maciejewski D, Hillegers M, Penninx B. Offspring of parents with mood disorders: time for more transgenerational research, screening and preventive intervention for this high-risk population. Current Opinion in Psychiatry. 2018;31(4):349–57.
27. Marconi A, Ranum N, Van Orman S, Hanson B, Donovan V, Borenitsch E. Demographic differences in response rates for PHQ9 in a university student population. Journal of American College Health. 2019;67(3):283–9.
28. Martins Cde S, Motta JV, Quevedo LA, Matos MB, Pinheiro KA, Souza LD, et al. Comparison of two instruments to track depression symptoms during pregnancy in a sample of pregnant teenagers in Southern Brazil. Journal of Affective Disorders. 2015;177:95–100.
29. Materu J, Kuringe E, Nyato D, Galishi A, Mwanamsangu A, Katebalila M, et al. The psychometric properties of PHQ-4 anxiety and depression screening scale among out of school adolescent girls and young women in Tanzania: a cross-sectional study. BMC Psychiatry. 2020;20(1):321.
30. Morelli N, Gusman M, Sinclair-McBride K. PHQ-9 Administration in Outpatient Adolescent Psychiatry Services. 2018;69:837–8.
31. Mphahlele, R. M., Pillay, B., & Meyer, A. (2020). Internalising comorbidities in primary school children with attention-deficit hyperactivity disorder (ADHD): Sex and age differences. *J*, *32*(2–3), 119–129. https://doi.org/10.2989/17280583.2020.1848851
32. Province of British Columbia. Establishing Priorities among Effective Clinical Prevention Services in British Columbia: Reference Document and Key Assumptions. 2018.
33. Rasmussen A, Eustache E, Raviola G, Kaiser B, Grelotti DJ, Belkin GS. Development and validation of a Haitian Creole screening instrument for depression. Transcultural Psychiatry. 2015;52(1):33–57.
34. Rinke ML, Bundy DG, Stein REK, O’Donnell HC, Heo M, Sangvai S, et al. Increasing Recognition and Diagnosis of Adolescent Depression: Project RedDE: A Cluster Randomized Trial. Pediatric Quality & Safety. 2019;4(5):e217.
35. Saez-Flores E, Tonarely NA, Barker DH, Quittner AL. Examining the Stability of the Hospital Anxiety and Depression Scale Factor Structure in Adolescents and Young Adults With Cystic Fibrosis: A Confirmatory Factor Analysis. Journal of Pediatric Psychology. 2018;43(6):625–35.
36. Schleider JL, Dobias M, Fassler J, Shroff A, Pati S, Miu NSSSSSWZ. Promoting treatment access following pediatric primary care depression screening: Randomized trial of web-based, single-session interventions for parents and youths. Journal of the American Academy of Child and Adolescent Psychiatry. 2020;59(6):770–3.
37. Schleider, J. L., Dobias, M., Fassler, J., Shroff, A., & Pati, S. (2020). “Promoting treatment access following pediatric primary care depression screening: Randomized trial of web-based, single-session interventions for parents and youths”: Correction. *Journal of the American Academy of Child & Adolescent Psychiatry*, *59*(12), 1408–1410. https://doi.org/10.1016/j.jaac.2020.09.012
38. Schmitt JA, Corathers S, Kichler J. Depression and suicidal ideation in adolescents with type 1 diabetes mellitus. Hormone Research in Paediatrics. 2017;88 (Supplement 1):234–5.
39. Sekhar DL, Ba DM, Liu G, Kraschnewski JL. Major Depressive Disorder Screening Remains Low Even Among Privately Insured Adolescents. Journal of Pediatrics. 2019;204:203–7.
40. Sterling SA, Kline-Simon A, Jones A, Brumder-Ross T, Weisner C. Outcomes from a trial of screening, brief intervention and referral to treatment for adolescents in pediatric primary care: Implications for adolescent girls. Alcoholism: Clinical and Experimental Research. 2017;41 (Supplement 1):336A.
41. Swetland, D. V., Hente, E., Hossain, M. M., Tadesse, D. G., Moore, S. E., Miller, J. L., Filigno, S. S., & Siracusa, C. (2020). Investigating longitudinal trends in anxiety and depression screening in pediatric cystic fibrosis. *Pediatric Pulmonology*, *55 (SUPPL 2)*, 274–275.
42. Thabrew H, D’Silva S, Darragh M, Goldfinch M, Meads J, Goodyear-Smith F. Comparison of YouthCHAT, an Electronic Composite Psychosocial Screener, With a Clinician Interview Assessment for Young People: Randomized Controlled Trial. Journal of Medical Internet Research. 2019;21(12):e13911.
43. Thurman TR, Nice J, Taylor TM, Luckett B. Mitigating depression among orphaned and vulnerable adolescents: a randomized controlled trial of interpersonal psychotherapy for groups in South Africa. Child & Adolescent Mental Health. 2017;22(4):224–31.
44. Tran TD, Kaligis F, Wiguna T, Willenberg L, Nguyen HTM, Luchters S, et al. Screening for depressive and anxiety disorders among adolescents in Indonesia: Formal validation of the centre for epidemiologic studies depression scale - revised and the Kessler psychological distress scale. Journal of Affective Disorders. 2019;246:189–94.
45. Valado T, Tracey J, Goldfinger J, Briggs R. HealthySteps: Transforming the Promise of Pediatric Care. Future of Children. 2019;29(1):99–122.
46. Cheung AH, Zuckerbrot RA, Jensen PS, Laraque D, Stein REK, GLAD-PC STEERING GROUP. Guidelines for Adolescent Depression in Primary Care (GLAD-PC): Part II. Treatment and Ongoing Management. Pediatrics. 2018;141(3):e20174082.
47. Corathers S, Mara CA, Chundi PK, Kichler JC. Depression Screening of Adolescents With Diabetes: 5-Years of Implementation and Outcomes. 2019;58:628–32.
48. Croke LM. Depression in Adolescents: AAP Updates Guidelines on Diagnosis and Treatment. American Family Physician. 2018;98(7):462–3.
49. Depression in children and young people: identification and management. Clinical Pharmacist. 2018;
50. Depression Screening and Follow-Up for Adolescents and Adults. :19.
51. Forman-Hoffman VL, Viswanathan M. Screening for Depression in Pediatric Primary Care. Current Psychiatry Reports. 2018;20(8):62.
52. Kaiser Permanente. Adult & Adolescent Depression Screening, Diagnosis, and Treatment Guideline. 2018.
53. Mackner LM, Whitaker BN, Maddux MH, Thompson S, Hughes-Reid C, Drovetta M, et al. Depression Screening in Pediatric Inflammatory Bowel Disease Clinics: Recommendations and a Toolkit for Implementation. Journal of Pediatric Gastroenterology and Nutrition. 2020;70(1):42–
54. Maurer DM, Raymond TJ, Davis BN. Depression: Screening and diagnosis. American Family Physician. 2018;98(8):508–15.
55. Screening for Depression in Children and Adolescents: Recommendation Statement. 2016;93(6):3.
56. Zuckerbrot RA, Cheung A, Jensen PS, Stein REK, Laraque D, GLAD-PC STEERING GROUP. Guidelines for Adolescent Depression in Primary Care (GLAD-PC): Part I. Practice Preparation, Identification, Assessment, and Initial Management. Pediatrics. 2018;141(3):e20174081.

### Systematic, narrative, or literature review (n=7)

1. Caldwell, D. M., Davies, S. R., Hetrick, S. E., Palmer, J. C., Caro, P., Lopez-Lopez, J. A., Gunnell, D., Kidger, J., Thomas, J., French, C., Stockings, E., Campbell, R., & Welton, N. J. (2020). “School-based interventions to prevent anxiety and depression in children and young people: A systematic review and network meta-analysis”: Correction. *The Lancet Psychiatry*, *7*(9). https://doi.org/10.1016/S2215-0366%2820%2930352-7
2. Nelson HD, Cantor A, Pappas M, Weeks C. Screening for Anxiety in Adolescent and Adult Women: A Systematic Review for the Women’s Preventive Services Initiative. Annals of Internal Medicine. 2020;173(1):29–41.
3. Richardson R, Trépel D, Perry A, Ali S, Duffy S, Gabe R, et al. Screening for psychological and mental health difficulties in young people who offend: a systematic review and decision model. Health Technology Assessment. 2015;19(1):1–128.
4. Roseman M, Kloda LA, Saadat N, Riehm KE, Ickowicz A, Baltzer F, et al. Accuracy of Depression Screening Tools to Detect Major Depression in Children and Adolescents: A Systematic Review. The Canadian Journal of Psychiatry. 2016;61(12):746–57.
5. Roseman M, Saadat N, Riehm KE, Kloda LA, Boruff J, Ickowicz A, et al. Depression Screening and Health Outcomes in Children and Adolescents: A Systematic Review. Canadian Journal of Psychiatry. 2017;62(12):813–7.
6. Thabrew H, McDowell H, Given K, Murrell K. Systematic Review of Screening Instruments for Psychosocial Problems in Children and Adolescents With Long-Term Physical Conditions. Global Pediatric Health. 2017;4:2333794X1769031.
7. Webb MJ, Kauer SD, Ozer EM, Haller DM, Sanci LA. Does screening for and intervening with multiple health compromising behaviours and mental health disorders amongst young people attending primary care improve health outcomes? A systematic review. BMC Family Practice. 2016;17(1):104.
8. Cheung AH, Zuckerbrot RA, Jensen PS, Laraque D, Stein REK, GLAD-PC STEERING GROUP. Guidelines for Adolescent Depression in Primary Care (GLAD-PC): Part II. Treatment and Ongoing Management. Pediatrics. 2018;141(3):e20174082.
9. Corathers S, Mara CA, Chundi PK, Kichler JC. Depression Screening of Adolescents With Diabetes: 5-Years of Implementation and Outcomes. 2019;58:628–32.
10. Croke LM. Depression in Adolescents: AAP Updates Guidelines on Diagnosis and Treatment. American Family Physician. 2018;98(7):462–3.
11. Depression in children and young people: identification and management. Clinical Pharmacist. 2018;
12. Depression Screening and Follow-Up for Adolescents and Adults. :19.
13. Forman-Hoffman VL, Viswanathan M. Screening for Depression in Pediatric Primary Care. Current Psychiatry Reports. 2018;20(8):62.
14. Kaiser Permanente. Adult & Adolescent Depression Screening, Diagnosis, and Treatment Guideline. 2018.
15. Mackner LM, Whitaker BN, Maddux MH, Thompson S, Hughes-Reid C, Drovetta M, et al. Depression Screening in Pediatric Inflammatory Bowel Disease Clinics: Recommendations and a Toolkit for Implementation. Journal of Pediatric Gastroenterology and Nutrition. 2020;70(1):42–
16. Maurer DM, Raymond TJ, Davis BN. Depression: Screening and diagnosis. American Family Physician. 2018;98(8):508–15.
17. Screening for Depression in Children and Adolescents: Recommendation Statement. 2016;93(6):3.
18. Zuckerbrot RA, Cheung A, Jensen PS, Stein REK, Laraque D, GLAD-PC STEERING GROUP. Guidelines for Adolescent Depression in Primary Care (GLAD-PC): Part I. Practice Preparation, Identification, Assessment, and Initial Management. Pediatrics. 2018;141(3):e20174081.

### Other language (n=2)

1. Galvan-Molina JF, Jimenez-Capdeville ME, Hernandez-Mata JM, Arellano-Cano JR. Psychopathology screening in medical school students. Gaceta Medica de México. 2017;153(1):75–87. [Spanish]
2. Winkler R Goetz, G, Schink, J, Reinsperger, I. Screening-/Vorsorgeprogramme für Kinder und Jugendliche von 6 bis 18 Jahren. Ergebnisse zu ausgewählten Ländern, Österreich-Programmen sowie Empfehlungen aus evidenzbasierten Leitlinien. LBI-HTA Projektbericht Nr.: 123. Wien: Ludwig Boltzmann Institut für Health Technology Assessment.; 2019. [Austrian]
