# Supplementary material for: Screening for depression in children and adolescents in primary care or non-mental health settings: a systematic review update
Source: Syst Rev. 2024 Jan 31;13:48. doi: 10.1186/s13643-023-02447-3 (PMC10829174; doi:10.1186/s13643-023-02447-3)
Supplement: Supplementary file 8 — Additional file 8. List of potentially relevant ongoing studies. [file 13643_2023_2447_MOESM8_ESM.docx]

## Additional file 8: List of potentially relevant ongoing studies

| **Trial Identifier** | **Study Title** | **Study Start Date** | **Estimated Study Completion Date** |
| --- | --- | --- | --- |
| NCT03716869  ClinicalTrials.gov | Randomized Controlled Trial of Universal vs. Targeted School Screening for Adolescent Major Depressive Disorder  Protocol: *Sekhar DL, Pattison KL, Confair A, Molinari A, Schaefer EW, Waxmonsky JG, Walker-Harding LR, Rosen P, Kraschnewski JL. Effectiveness of Universal School-Based Screening vs Targeted Screening for Major Depressive Disorder Among Adolescents: A Trial Protocol for the Screening in High Schools to Identify, Evaluate, and Lower Depression (SHIELD) Randomized Clinical Trial. JAMA network open. 2019 Nov 1;2(11):e1914427-.* | July 2018 | December 2020 |
| NL7719  Netherlands Trial Register | Strong teens and resilient minds: Depression and Suicide Prevention in Higher Vocational Education | October 2017 | October 2021 |
| NL6444/NTR6622  Netherlands Trial Register | Strong teens and resilient minds: Depression and Suicide Prevention in Secondary Education  Protocol: *Gijzen MW, Creemers DH, Rasing SP, Smit F, Engels RC. Evaluation of a multimodal school-based depression and suicide prevention program among Dutch adolescents: design of a cluster-randomized controlled trial. BMC psychiatry. 2018 Dec;18(1):124.* | September 2017 | October 2021 |
| DRKS00012504  German Clinical Trials Register | The CARE for CAYA program - Comprehensive Assessments and Related interventions to Enhance long-term outcome in Children, Adolescents and Young Adults (CAYAs) | January 2018 | Not reported  (Recruitment ongoing) |
