# Supplementary material for: Screening for depression in children and adolescents in primary care or non-mental health settings: a systematic review update
Source: Syst Rev. 2024 Jan 31;13:48. doi: 10.1186/s13643-023-02447-3 (PMC10829174; doi:10.1186/s13643-023-02447-3)
Supplement: Supplementary file 9 — Additional file 9. Stakeholder feedback. [file 13643_2023_2447_MOESM9_ESM.docx]

## Additional file 9: Stakeholder feedback

**Document sent to stakeholders**

Thank you again for reviewing the evidence review manuscript: ***Screening for depression in children and adolescents in primary care or non-mental health settings: a systematic review update***. This evidence review focuses on screening for depression among children (6 to 11 years old) and adolescents (12 to 17 years old) in primary care and non-mental health clinic settings. This evidence review will be used to develop guidelines.

**INSTRUCTIONS:**

- Please use this form to provide feedback on the evidence review and return this form no later than **July 14, 2023,** to **taskforce.admin@CTFPHC.onmicrosoft.com**
- If you have any questions related to the review process, contact Melissa Subnath at [**melissa.subnath@phac-aspc.gc.ca**](mailto:melissa.subnath@phac-aspc.gc.ca).
- Please check the appropriate box to answer the questions and elaborate in the space provided if necessary.

| **Question 1** | **Yes** | **No** |
| --- | --- | --- |
| Are the objectives and methods of this evidence review clear? | **​​☐​** | **​​☐​** |
|  | **Comments:** | |
| **Question 2** | **Yes** | **No** |
| Were the results clearly stated? | **​​☐​** | **​​☐​** |
|  | **Comments:** | |
| **Question 3** | **Yes** | **No** |
| Are the conclusions in the review supported by the data that were reviewed? | **​​☐​** | **​​☐​** |
|  | **Comments:** | |
| **Question 4** |  | |
| Do you have any additional comments? |  | |

**Stakeholder feedback**

| **Question** | **Comment** | **Reviewer** |
| --- | --- | --- |
| Question 4: Additional comments | The authors may want to hand check that no RCTs from this recent systematic review meet their prespecified selection criteria**.**  Viswanathan M, Wallace I, Middleton JC, et al. Screening for Depression, Anxiety, and Suicide Risk in Children and Adolescents: An Evidence Review for the U.S. Preventive Services Task Force [Internet]. Rockville (MD): Agency for Healthcare Research and Quality (US); 2022 Oct. (Evidence Synthesis, No. 221.) Available from: <https://www.ncbi.nlm.nih.gov/books/NBK585407/> | Reviewer #1 |
| Question 2: Results | **Comments: Limitations can be more robust.**  Page 22- strong opinion stating no studies gave evidence on reducing depressive symptoms, improving quality of life or improving suicidal risk – when out of the 8 studies that met some inclusion criteria, these areas were not discussed. | Reviewer #2 |
| Question 4: Additional comments | It was very interesting to read the manuscript which presented the information very clearly. It would be valuable to see the outcomes of further research. | Reviewer #3 |
| Questions 1 to 4 | The Objective and Methods are clearly reported with sufficient detail.  The Results are clear. Narrative and tabular reporting are consistent and facilitate understanding.  Strictly speaking, the conclusions are supported by the data. There are no RCT studies that meet the stringent criteria laid out by the study authors. Moreover, there is a need for methodologically rigorous studies to evaluate the harms and benefits of screening measures.  The criteria for study inclusion are very strict. While studies need to be conducted with maximal rigour, the complete absence of studies meeting criteria may be related to the criteria not being realistic for a real-world setting. Not recommending depression screening feels counterintuitive to me as I worry more about Mental Health needs being missed than asking about mood /depression causing harm, particularly in primary care settings. Of note, suicidal behaviour is increasing in young people. Depression and suicidal behaviour are related. Routine suicide risk screening does seem related to identifying more at-risk youth with possibility of provided needed intervention. (Chloe C. Milliman, Patricia A. Dwyer, Judith A. Vessey, Pediatric Suicide Screening: A Review of the Evidence, Journal of Pediatric Nursing, Volume 59, 2021, Pages 1-9, ISSN 0882-5963,  https://doi.org/10.1016/j.pedn.2020.12.011.). The manuscript under review should minimally make reference to these issues. | Reviewer #4 |
| Question 3 & 4 | Makes me more comfortable with not having the time to screen that it will not impact harm. So many screening guidelines leave family physicians feeling guilty if they don’t have the time to do them in this era of collapsing primary care.  Might want to highlight the other benefit of not screening is to not to increase physician/primary care provider burdens with screening measures that is not supported by evidence. | Reviewer #5 |
| Question 4 | Health disparities is barely touched on as a part of the background/burden. The USPSTF published its most recent depression recommendation in 2022 but you cite the 2016 recommendation on page 23 as “the USPSTF’s most recent guideline.” | Reviewer #6 |
| Question 3 | Lack of evidence re benefits and harms of screening for depression = yes page 26 clearly states this | Reviewer #7 |
| Question 4 | The first sentence on page 12 has 2 periods at the end of the sentence. | Reviewer #8 |
| Question 4 | When considering implications for future clinical trials and research, it is important to consider the length of time participants would be followed for in order to accurately measure the critical outcomes being considered for the purpose of this study as these outcomes can change over time. | Reviewer #9 |
| N/A | No comment. | Reviewer #10 |
